# Robust, Causal, and Incremental Approaches to Investigating Linguistic Adaptation

**DOI:** 10.3389/fpsyg.2018.00166

**Published:** 2018-02-21

**Authors:** Seán G. Roberts

**Affiliations:** EXCD Lab, Department of Anthropology and Archaeology, University of Bristol, Bristol, United Kingdom

**Keywords:** adaptation, humidity, tone, vowels, robustness, causal graph

## Abstract

This paper discusses the maximum robustness approach for studying cases of adaptation in language. We live in an age where we have more data on more languages than ever before, and more data to link it with from other domains. This should make it easier to test hypotheses involving adaptation, and also to spot new patterns that might be explained by adaptation. However, there is not much discussion of the overall approach to research in this area. There are outstanding questions about how to formalize theories, what the criteria are for directing research and how to integrate results from different methods into a clear assessment of a hypothesis. This paper addresses some of those issues by suggesting an approach which is causal, incremental and robust. It illustrates the approach with reference to a recent claim that dry environments select against the use of precise contrasts in pitch. Study 1 replicates a previous analysis of the link between humidity and lexical tone with an alternative dataset and finds that it is not robust. Study 2 performs an analysis with a continuous measure of tone and finds no significant correlation. Study 3 addresses a more recent analysis of the link between humidity and vowel use and finds that it is robust, though the effect size is small and the robustness of the measurement of vowel use is low. Methodological robustness of the general theory is addressed by suggesting additional approaches including iterated learning, a historical case study, corpus studies, and studying individual speech.

## 1. Introduction

The goal of evolutionary approaches to linguistics is to explain similarities and differences between languages. As Bickel (2015) might put it, “what's where why?.” The final part of this question—why—is crucial. It requires the demonstration of causal effects including how languages adapt to functional pressures. This is not an easy task. It involves dealing with long causal chains stretching from biology, cognition, and interaction to many different areas of language. It also involves dealing with many possible alternative explanations and the complexities of linguistic history. Some parts of adaptational explanations can be addressed directly with controlled experiments. However, because of the range of timescales involved it is inevitable that some of the steps are addressed with more abstract methods such as modeling, artificial language learning, or historical reconstruction. How can we combine results from such different approaches into coherent evidence for or against a particular theory? Many studies seeking to show adaptation in human language also rely on large-scale global databases. Indeed, we are experiencing a kind of gold rush of cross-cultural statistical studies, where it feels like anyone with a laptop and access to the internet could find the next big discovery in cultural evolution (Ladd et al., 2015). However, there is not much discussion, within the field of evolutionary linguistics at least, of the general strategy for using these new data and methods to address questions of adaptation. This paper presents one general strategy and discusses its advantages. It outlines concrete, explicit steps which help formulate and communicate questions more clearly and arrive at a clearer understanding of the answers. The main point I would like to make is that, when dealing with cross-cultural statistical methods, there is no single smoking gun that will definitively prove a theory correct, nor a single magic bullet that will disprove it entirely.

The strategy that I will advocate has several features. It is causal, incremental and robust. I will call this the *maximum robustness method*. It is useful to contrast this with what might be called the *maximum validity method*. Briefly, the maximum validity method proceeds as follows:

- Set out a series of specific assumptions and claims that your hypothesis makes.
- Collect and code your data according to your assumptions.
- Run the most valid test given your data and assumptions.
- The outcome is the best answer given the assumptions.

That is, it attempts to perform the most relevant and valid test of a specific hypothesis, then accepts the single result as the best possible evaluation. This may be a caricature of a possible approach to science, but I suspect it is probably the default approach in most individual studies in linguistics. One study that exemplifies this is Hammarstrom (2010), which tests whether the adoption of farming practices leads to higher rates of dispersal and so language families with greater numbers of languages. Hammarström collected data on “ALL attested language families” (about 7,000 languages, capitalisation in the original) and quantifies the number of languages within each given explicit assumptions. Each language was classified as having either an agricultural or hunter-gatherer subsistence type. Then a single, bespoke independent samples test was run to determine the result.

Of course, all statistical analyses should aim to be valid. However, given the range of methods and possible measures available now, it is often difficult to identify the single most valid approach. Indeed, Silberzahn and Uhlmann (2015) gave the same dataset and research question to several researchers to analyse independently. The results varied widely in terms of effect size due to differences in the statistical approaches, yet all of them were defensible. Trying to argue for the most valid approach may lead to arguments from authority rather than logic, so another approach is the maximum robustness method:

- Set out a series of general assumptions and claims that your hypothesis makes.
- Run tests with as many specific assumptions and sources of data as possible
- Demonstrate that all tests give qualitatively the same answer, **or** identify similarities in approaches which lead to negative results.
- The outcome is a space of results that suggests how robust the hypothesis is.

As Levins (1966) put it, “Our truth is the intersection of independent lies.” An example of the maximum robustness approach is found in Roberts et al. (2015), which reconsidered the link between future tense and economic variables first proposed by Chen (2013). The correlation in the initial study was strong, but did not account for the effect of shared linguistic history, leaving open the possibility that the correlation was an artefact of Galton's problem. Instead of presenting one methodology which would have provided a single answer (the maximum validity method), Roberts et al. (2015) used nine different statistical methods and two datasets to address the question. They produced a space of results and linked each one to the assumptions of its method. They found that the correlation did appear to be robust, except when the method allowed four key factors: using individual level data as opposed to collapsing within languages; controlling for local economic effects within countries; controlling for cultural descent within language families; and controlling for areal contact. When all these controls were applied the correlation was not significant.

The maximum robustness method also discourages the idea that there is a single best analysis which definitively proves or disproves a theory. The space of results should tell us more than simply that the first paper was flawed: it suggests that collapsing information within languages loses some important aspects of the data, and that all three of the historical processes are at play in human cultural evolution (see also Moran et al., 2012). Furthermore, the ultimate suggestion of the paper was that large-scale, cross-cultural statistics was not the best approach for addressing this question due to the complexities of the confounding factors, and instead future research should concentrate on localized experiments, which are quite feasible in this case (Thoma and Tytus, 2017).

Aspects of both approaches are, of course, part of the ideal scientific method, particularly the careful expression of assumptions from the maximum validity method, and the repeated testing of the maximum robustness method. However, due to limited resources or data, most studies tend to gravitate toward maximum validity. In particular, the complexity of running many different tests in the robustness approach and the difficulty of reconciling conflicting results makes the maximum robustness method difficult to conduct in a single paper. I will argue that the robust approach is worth it, but also probably needs to be combined with an incremental approach in order to be effective. The combination of robust and incremental approaches is particularly useful for studies of cultural evolution where there may be a long chain of causal connections that span many disciplines and a large range of appropriate methodologies to address each link.

The paper is organized as follows. The rest of this section summarizes the hypothesized adaptive link between tone and humidity which will be used as a case study in the rest of the paper. In sections 2–4, the features of the maximum robustness approach are presented. Section 2 shows how causal graphs can be used in a six-step process to map out an explicit expression of a hypothesis, its implications and potential confounds. Section 3 discusses the idea of incremental research and how gradual progress toward support for a hypothesis is the most pragmatic approach. Section 4 discusses the final part of the approach, which is robustness: converging evidence from many angles provides the best way of supporting adaptive hypotheses.

Sections 5–7 apply some of the ideas from the maximum robustness approach to the hypothesis linking tone and humidity. Section 5 attempts to replicate previous analyses with an alternative source of data to ensure their robustness. Section 6 suggests some hypothetical ways that other links in the causal chain linking humidity and tones could be tested. Section 7 summarizes the current state of the hypothesis given the evidence presented here. Section 8 provides a brief conclusion.

### 1.1. Case study: linguistic adaptations to humidity

Everett et al. (2015) suggested that the distribution of languages that use lexical tone across the world could be predicted by humidity. This work will be used as a case study to illustrate the maximum robustness method. The choice is not intended to suggest that it is robust. Indeed, it is because this is a controversial idea that it serves as a good example and would benefit from the maximum robustness approach.

The idea of trying to explain properties of language as being adapted to external climatic influences goes back a long way. As far back as the late eighteenth century, de Rivarol suggested that languages are “melodious and voluptuous in mild climes, harsh and dull under a sad sky” (de Rivarol, 1784), and Lord Monboddo hypothesized a link between laryngeal desiccation, production, and the distribution of particular sounds in language:

“But the total want of P and W may be looked on as the grand literal distinction, between the Scandinavian and the German dialects of the Gothic. And this seems a remarkable instance of the effect of climate upon language; for P and W are the most open of the labial letters; and V is the most shut. The former requires an open mouth: the later may be pronounced with mouth almost closed, which rendered it an acceptable substitute in the cold climate of Scandinavia, where the people delighted as they will delight, in gutturals and dentals. The climate rendered their organs rigid and contracted; and cold made them keep their mouths as much shut as possible.”

(Pinkerton, 1789, p. 19)

These are, of course, too limited (and poetical) to constitute substantial, rigorous evidence for the proposed link, but modern databases and statistical methods allow us to test these hypotheses quantitatively. This has been done for links between climate and phonetics (see Munroe et al., 2009; Maddieson and Coupé, 2015). More recently, Everett et al. (2015, 2016a) reviewed research from laryngology showing that dry air affects the vocal folds, making careful control of pitch difficult. There are many cases of animal signals adapting to environmental conditions such as humidity (e.g., Snell-Rood, 2012). This suggests that human languages would also adapt to the local humidity over long periods of time so that careful control of pitch (e.g., complex lexical tone systems) would be rarer in drier regions. This was tested in a sample of around 3,000 languages and moderate support was found for the hypothesis that complex tone languages were rarer in drier regions.

The more general theory that desiccation affects the vocal folds was recently extended in a paper in this issue to predictions about vowels. Since vowels require more precise control of vocal folds than consonants, they should also be relied upon less in drier regions. Accordingly, Everett (2017) shows that speakers in drier regions use vowels less frequently in their basic vocabulary.

This theory fits within the “distributional typology” approach, which attempts to explain patterns in typological variables as causal effects from functional pressures or historical events using statistical analyses (Bickel, 2015). However, most previous analyses in this vein have concentrated on functional pressures from cognition (e.g., Bickel et al., 2015), rather than physical pressures from the ambient climate. Accordingly, the link between tone and humidity has been criticized on many grounds, both methodological and theoretical (see Everett et al., 2016a and responses: Collins, 2016; de Boer, 2016; Donohue, 2016; Ember, 2016a; Gussenhoven, 2016; Hammarström, 2016; Ladd, 2016; Moran, 2016; Progovac and Ratliff, 2016; Winter and Wedel, 2016). The aim of this paper is not to address those criticisms, but to attempt to use this research to illustrate the maximum robustness method. Section 5 tests the robustness of the claim about tones and section 6 tests the robustness of the claim about vowels.

## 2. Causal graphs

This section presents the six steps for using causal graphs in a maximum robustness approach to research. The first step of this approach (and many others) is to be explicit about the causality of the claims in the hypothesis. This seems like a trivial requirement for any investigation, but is a subtlety difficult challenge (defining causality itself is tricky, and I avoid doing so here, but see Blasi and Roberts, 2017 for a discussion relating to humidity and tone). As every researcher knows, discovering a simple correlation is not the same as proving a causal link. The gold standard for demonstrating causality is a controlled experiment, but often correlations are the first step toward this ultimate goal. More importantly for this paper, behind each study there should be at least a hypothesis about a causal effect, and that is what this section is interested in capturing. One of the clearest methods for defining causal relationships is by using causal graphs (e.g., Pearl, 2000). Nodes represent variables and edges represent casual processes. As well as an investigative methodology in its own right, causal graphs can be used as a tool for helping researchers to think about their hypotheses and to guide the direction of research. For example, the basic causal claim in Everett et al. (2015) is that ambient humidity causes a change in the number of distinctions in tones. However, this leaves out many processes in between and many other possible explanations of a statistical link. Here, I will suggest a number of steps to help arrive at a full causal picture of the domain of the hypothesis.

Step (1) Draw the main causal link between the elements of the hypothesis.

These are usually the measurable variables mentioned in the prose formulation of the hypothesis. In this case:

Ambient Humidity → Fewer tones

**Figure 1:**
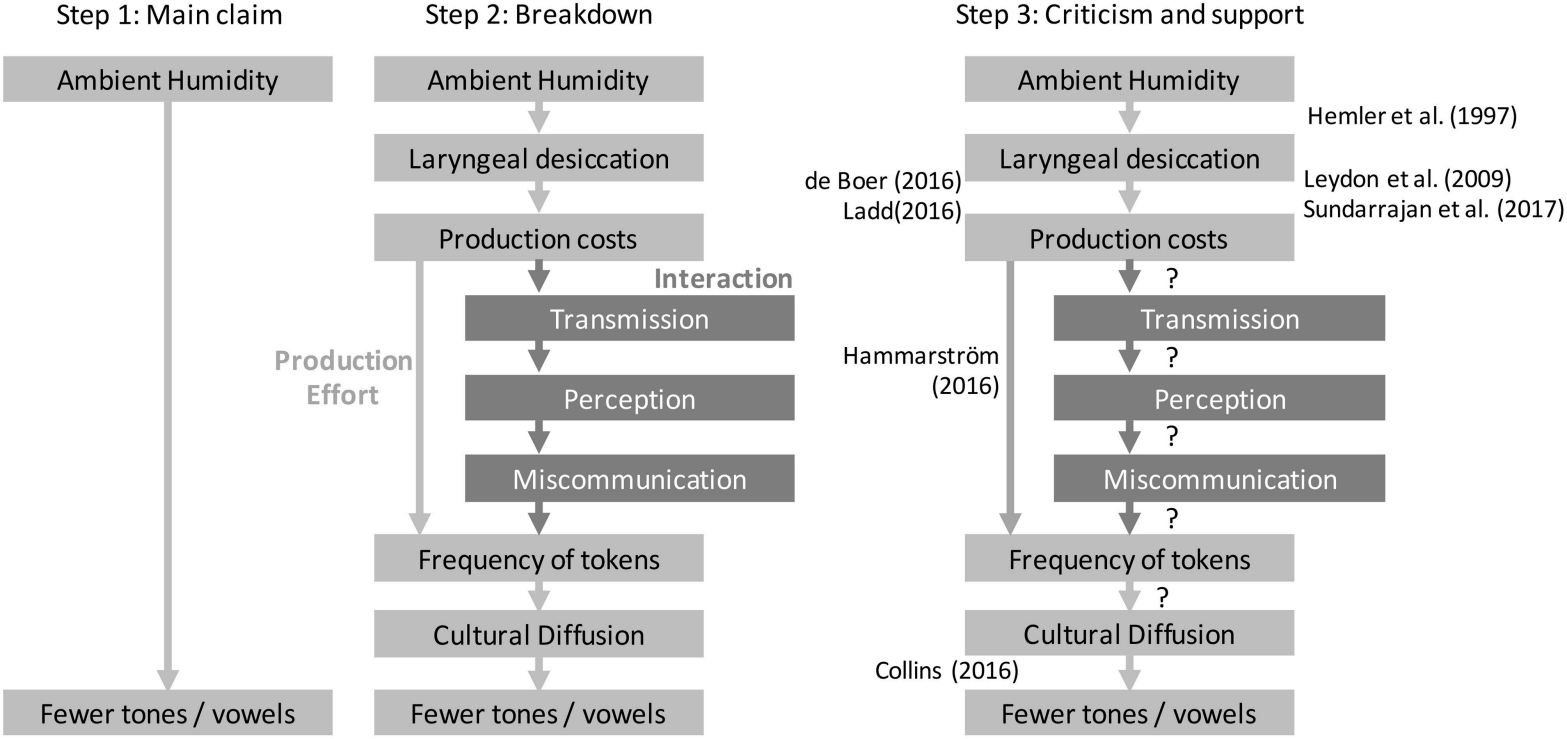
The first three steps in the causal approach. Left: Step 1, starting with the main causal claim. Middle: Step 2, breaking the main claim down into a chain of links. Right: Step 3, assessing criticism and support. Criticisms are listed on the left and supporting evidence is listed on the right. Question marks represent no supporting evidence for the particular link.

This is depicted in Figure 1.

Step (2a) Break down the main causal link into more fine-grained causal links.

The goal is to identify more local links to spell out a more detailed description of the causal process. This involves being more explicit about the physical causality, and often in the case of global statistical studies involving adaptation, about the mechanisms of propagation and diffusion. The result is a chain of causal links. For the case of humidity and tone, one could imagine the chain of causal effects based on production effort:

Ambient Humidity → Laryngeal desiccation → Production costs → Frequency of tokens → Cultural diffusion → Fewer tones

That is, ambient humidity causes desiccation of the larynx and vocal folds, which affects production (finer control of fundamental frequencies requires more production effort). This leads to a change in the frequency of tokens (fewer tokens involving complex pitch). Through cultural diffusion, this could lead to a change in the linguistic system as a whole so that there were fewer distinctions in lexical tone.

Step (2b) Consider alternative causal pathways between the elements of the chain that would also support the hypothesis.

The goal here is to imagine alternative pathways between the main causal variables, or any of the other links already described. In the case of tone and humidity, there is an alternative pathway involving *interaction*: The affects of desiccation on production leads to weaker distinctions being transmitted, this influences perception in the listener, leading to miscommunication, and eventually to a selection pressure against fine tonal distinctions (Figure 1).

Step (3) Asses the current evidence for each causal link.

For each causal link, is there causal evidence that supports it? The best evidence might come from controlled experiments, but may also include causally informed statistical work or theoretical work. For example, there are several experiments that relate to the causal links between humidity and tone (see Everett et al., 2015, 2016a). For example, Hemler et al. (1997) demonstrate that humidity affects vocal fold vibration accuracy. Leydon et al. (2009) and Sundarrajan et al. (2017) demonstrate that vocal fold vibration causes changes in production. At the same time, criticisms of each causal link can be added to suggest negative evidence (Figure 1).

Step (4) Place the causal graph in a wider context.

The next step is more challenging. It requires thinking outside of the narrow focus of the hypothesis into any other possible causal links that might interfere with the main causal pathway. Two types of link in particular should be sought. The first is anything that directly affects the final variable (i.e., number of tones). The second is any series of links that provide an alternative causal link between the two main variables that are not part of the general hypothesis. The goal is to find any causal link going from new variables to the final causal variable that, to put it technically, are not d-separated by the first causal variable. That is, there are plenty of things that affect humidity, but what we are interested in is things that affect humidity and also the final variable, possibly with intermediate steps. It is likely that these links will come from outside of the field of linguistics (Bickel and Nichols, 2006).

One possible alternative pathway is a direct effect of humidity on sound transmission via sound absorption. This link is well-understood at a basic physical level (humid air conducts higher frequencies better, Bass et al., 1984) and many animal communication systems show adaptation to this constraint (Snell-Rood, 2012). It is unclear whether this would cause the same selection pressure as the production effort caused by laryngeal desiccation, but at this point it is worth considering. Another possible pathway is a direct effect of humidity on perception. There is some weak evidence that repeated exposure to dry, cold environments damages the ear in a way that could influence perception (Morgan, 1954). This is an unlikely explanation, but at this point the goal is to list possible causal links, not to evaluate them.

**Figure 2:**
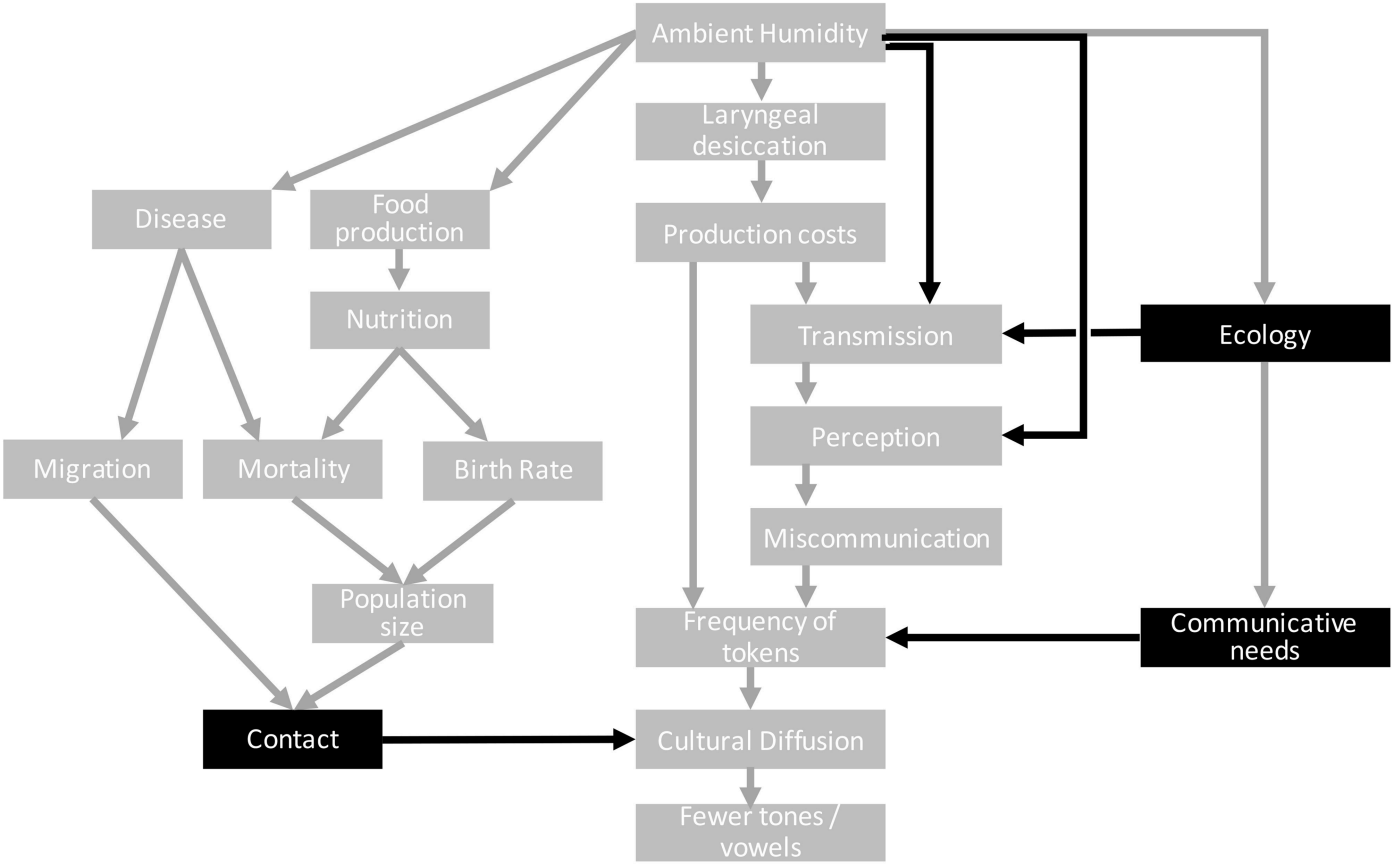
Steps 4 and 5 of the causal approach. Additional nodes represent the wider causal context. Nodes and arrows in black indicate potential confounds that need to be addressed.

An example of a wider context was suggested in Everett et al. (2015), and is redrawn in Figure 2. It includes links from the literature on climate, disease and migration (Michaelowa, 2001; Ember, 2016a). The climate affects various demographic and disease-related variables which contribute to the likelihood of contact between languages and so possibly the eventual borrowing of tones in some climatic regions but not others. This could explain a statistical link between the climate and the distribution of tone that is not part of the core claim of the original hypothesis.

Other alternative pathways include a link between the ecology (density of foliage in the environment) and acoustic transmission. This has been explored as an alternative hypothesis linking the environment and linguistic sounds (Morton, 1975; Fought et al., 2004; Ey and Fischer, 2009; Munroe et al., 2009; Maddieson and Coupé, 2015; Coupé, 2017; Maddieson, under review). The ecology may also influence the kinds of meanings that speakers need to talk about and the semantic, pragmatic and social distinctions that are important to them (Regier et al., 2016), which may affect the frequency of tokens.

Step (4) Identify possible confounds.

Given this wider picture, it should now be possible to identify causal factors that provide alternative causal explanations for a correlation between the two main variables. The causal graph may now be quite complicated, but we can use tools from causal graph theory to focus our attention on relevant potential confounds. For example, the wider causal graph above includes a large number of variables to do with demography and disease. However, the only place where this influences the main causal pathway is through contact. Therefore, if we can somehow control for the influence of contact on diffusion, then it follows that controlling for the demographic and disease variables is redundant. This is a Markov causal condition which is one of the fundamental parts of causal graph theory: variables can only be influenced by directly connected ancestors (this assumes that the causal graph drawn by the researcher is correct). This is an important point when considering control variables. Not every variable which is *correlated* with the main dependent needs to be controlled for, only those with a plausible direct causal influence.

Figure 2 shows the alternative pathways with the variables that interact with the main causal pathway highlighted. There are potentially many more confounding factors (e.g., having the right conditions for tonogenesis), but the point here is to demonstrate how thinking with causal graphs helps to make concrete the claims of a hypothesis and identify possible confounds.

Step (6) Choose the next link to research.

Given the final causal graph, it should now be possible to identify the next best step in the research programme. In the current example, it is clear that the question of historical diffusion and the confound of borrowing needs to be addressed. Beyond that, other suggestions are presented. For example, the interaction, sound absorption and perception pathways all rely on creating problems in miscommunication. Therefore, investigation into those mechanisms might begin with that link. More generally, the production effort pathway requires fewer causal steps, and so might be easier to investigate first. It is also possible that the evaluation of evidence and confounds will suggest that the hypothesis is not worth pursuing at all.

### 2.1. Advantages of the causal approach

Producing a causal graph such as the one above has several advantages for the large-scale statistical studies in linguistics.

#### 2.1.1. Clear communication of the hypothesis

Expressing hypotheses as detailed causal graphs forces researchers to be explicit about their claims. This avoids confusion and focuses criticism on specific issues. Together with an empirical approach, this should lead to more productive debate between researchers, because criticisms can address assumptions and data on particular points, rather than criticising a whole approach or the author themselves. One of the weaknesses of the maximum validity approach is that it relies on the judgement of the authors about what the most valid approach is. If a critic disagrees on the choice of a particular step in the analysis, it is difficult to interpret the value of the result. Figure 1 links some of the criticisms to particular links in the causal chain, indicating where improvement needs to be made.

#### 2.1.2. Identification of strong and weak links in the causal chain

By linking evidence to particular causal links, it should become clear which parts of a hypothesis are well supported and which require more investigation. Regarding humidity and tone, there is already experimental evidence for many of the early steps in the causal chain. There are three broad regions that remain to be tested. The first is the link between production costs and frequency of tokens, either directly or through interaction. The second is the link between frequency and the current distribution of tone systems in the world through cultural diffusion. The third is the potential confounding influence of other factors, particularly borrowing.

#### 2.1.3. Identification of possible confounds

The procedure above encourages an attempt to think of possible confounds and identify where in the causal chain they might apply. In the section above, the Markov causal condition was discussed which means that not all variables involved in alternative accounts necessarily need to be controlled for. This saves time and focusses research on relevant issues. It is worth noting that accounting for alternative influences on the key variables does not always reduce statistical power. In some cases, it may account for other noisy processes and reveal a causal effect in the main causal chain.

#### 2.1.4. Deconstruction of the problem into sub-hypotheses that can be addressed separately with different methods

The first 2 weak areas of the causal chain above may not be amenable to strict experimental control. In particular, the diffusion of linguistic variants is hard to study directly because of the timescales involved. However, the advantage of creating this causal graph is that it breaks the investigation down into smaller links, and each of these links can be investigated in its own right with the most appropriate methods and data. While physical acoustics and laboratory phonetics methods can be applied to the initial parts of the chain, there are more appropriate methods for the later parts including computational modeling (Kandler and Steele, 2008; Gavin et al., 2017), artificial language learning experiments (Tamariz, 2017b), historical corpus analyses and historical computational techniques such as phylogenetic ancestral state reconstruction (Gavin et al., 2013; Honkola et al., 2013).

One clear example of this modular approach is in the recent research into the link between genetics, vocal tract morphology, sound production, and global distributions of sound inventories (Dediu et al., 2017). The hypothesis was expressed as a chain of individual links, where each link was addressed with the most relevant method. For example, the first causal link is between genetic differences and individual differences in vocal tract anatomy, such as the shape of the hard palette. This was investigated with clinical measurements and backed up by evidence form developmental biology (Dediu and Moisik, 2016). Those physical differences have small effects on the effort required to produce particular sounds, causing biases in speech production. This was tested with a computational model of biomechanics and a cross-cultural phonetic learning experiment (Moisik and Dediu, 2017). The biases are amplified by cultural evolution into phonetic differences at the population level. This was tested by using the biomechanical model as an agent in an iterated agent based model and testing the effect of multiple generations of diffusion (Janssen et al., 2016). This predicts that physical differences cause the patterns of phonological inventories that we see in the world, which was tested on a database of worldwide phonology (Dediu et al., 2017).

#### 2.1.5. Guidance for incremental approaches

The causal graph, together with the evaluation of current evidence and potential confounds, should suggest the next steps for testing the hypothesis. Researcher resources are limited, and not all avenues can be explored. This method helps identify the most pragmatic way forward. This aids an incremental approach, which is discussed below.

## 3. Incremental research

I argue that research into cultural evolution should be incremental in three senses. First, it should build upon existing theories, typologies and knowledge from linguistics and other fields, rather than use new approximations that fit the data or model. This is not entirely straightforward to assess. For example, for many historical and descriptive linguists, the link between the physical climate and phonology was new and apparently motivated by spotting a pattern in the world. However, from a background in laryngology, acoustic physics or animal communication, the theory is a logical progression of some well-known phenomena.

Secondly, there is no need for every paper to prove the theory in its entirety. Instead, it is best to see a theory as a causal chain with many links, and researchers can investigate one link at a time. Each link may be best addressed by different methods and data (see above). Indeed, with recent advances in digital data curation, it is now possible to constantly update data and analyses. For example, PHOIBLE (Moran et al., 2014) and Glottolog (Hammarström et al., 2017) are constantly updated through github (see https://github.com/phoible/dev and https://github.com/clld/glottolog). We need no longer see a paper as the definitive last word on a dataset.

Finally, research might move from correlational to causal evidence in stages. Realistically, researchers will start with links that are easier to demonstrate given current data and advance toward more definitive, carefully controlled evidence. For example:

1. Demonstrate a synchronic relationship.
2. Demonstrate a diachronic relationship.
3. Demonstrate experimental evidence.

In parallel, researchers should attempt to elicit and disprove alternative explanations. Given the complexity of working between multiple fields, this will also be an incremental and interactive task. For example, based on criticisms and suggestions by Hammarström (2016) of the statistical methodology in the original paper on humidity and tone, Everett et al. (2016b) improved the method and re-ran the statistics (see below). It may be much easier to demonstrate confounds in a study than to correct for those confounds, which might mean that the possible criticisms of a hypothesis might develop much more quickly than the positive evidence for the hypothesis. One example of this comes from work in Collins (2016), which includes a computational simulation of a confounding mechanism (the diffusion of tone through local borrowing) before a simulation of the climatic hypothesis was developed. Given the slow progress of studies with new methods, it would be rash to dismiss (or fully accept) the original idea on the basis of a single study, and the incremental method advises patience on the part of researchers.

Indeed, one way to see early correlational studies is as “feasibility studies.”

## 4. Robustness in cross-cultural statistical research

This section discusses different kinds of robustness and how they relate to cross-linguistic analyses. Types of robustness discussed include measurement robustness, structural robustness, representational robustness, methodological robustness, estimation robustness, and robustness against *ad hoc* hypotheses. The section ends with a short summary of how the causal thinking, incrementality, and robustness can be combined to form the maximum robustness approach.

Robustness is a term used in many areas of research, but particularly in the use of computational and statistical modeling (Levins, 1966; Weisberg, 2006; Weisberg and Reisman, 2008; Wimsatt, 2012). Robust results are ones that hold under a range of assumptions. Seeking robustness is desirable when a models makes assumptions about various processes and quantities that cannot be confirmed in the real world. Weisberg and Reisman (2008) discuss different kinds of robustness based on different kinds of assumptions: structural robustness relates to assumptions about the causal structure of a model and parameter robustness relates to the range of model parameters under which a result holds. Macro economics studies often test the stability of an estimate of the strength of a relationship between two variables in a regression when adding a range of alternative control variables (Leamer, 1985). There is also some discussion about whether robustness provides proof of a causal relationship as opposed to a mere correlation, though Woodward (2006) is doubtful that this is logically sound.

In cross-cultural statistical analyses, there are many different kinds of assumptions that could affect a result, and so many types of robustness which might be desirable. The sections below discuss some of them, moving from well-established types such as measurement robustness, structural robustness, and representational robustness to a discussion of some types of robustness that apply particularly to theories of cultural adaptation in linguistics (methodological robustness, estimation robustness, robustness against *ad hoc* hypotheses).

### 4.1. Measurement robustness

Woodward (2006) discusses *measurement robustness*: the measurement of a variable is robust if different independent methods or measurement events agree. Most psycholinguistic studies that involve manual coding often assess reliability by comparing the judgements of multiple independent coders (e.g., using Cohen's κ, Cohen, 1968). Often in large-scale statistical analyses, we assume that the measurement of the variables is accurate and unbiased, but without trying to confirm this. Because quantifying aspects of a linguistic system is not an entirely objective process, it is likely that there are biases in measurement based on factors such as the theoretical background of the linguist (Moran, 2012, 2016; Easton et al., 2015). However, testing reliability is difficult due to the scarcity of multiple, independent sources for global linguistic data and the difficulty of finding proficient coders for some languages (though fluency is not always needed, see Dingemanse and Enfield, 2015). Additional independent measures are not possible for extinct languages with only one source. However, for some variables there are independent measures. For example, there are at least two databases counting the number of tones in a language (PHOIBLE, Moran et al., 2014, and the ANU phonotactics database, Donohue et al., 2013, see also Allison et al., 2006). Section 5.2.1 tests the robustness across these databases.

Beyond checking that the measures correlate, it is also important to test whether the measures are systematically biased for a particular language family or area, or according to the main dependent variable, which is also done below. This issue also applies to typological interpretation of primary sources. For example, the Glottobank project (http://glottobank.org/) is constructing a typological database of language structures based on primary materials such as grammar descriptions. The reliability of codings from multiple coders was measured.

It is likely that measurement robustness will be better for more concrete, lower-level features than for high-level categories. The “multivariate typology” approach suggests that high-level typological categories often do not capture the full similarities and differences between languages, and instead encourages linguists to break down abstract distinctions into “maximally fine-grained features” (see Bickel, 2010, 2011; Bickel et al., 2014). For example, instead of classifying a language as having “SVO” word order, that category can be broken down into different features that encode the word order in different contexts. Studies have shown that it is possible to do this to distinguish between dialects (Spruit, 2006) and for other domains such as for phonology (Macklin-Cordes and Round, 2015). Probabilistic typologies go one step further by coding the probability or frequency with which a particular construction is observed, building in an inherent measure of uncertainty (Bickel et al., 2009).

The maximum robustness approach differs from the maximum validity approach with regards to the importance of measurement robustness. The maximum validity aims to cover as many languages as possible with a particular typology, prioritising collecting data on currently uncoded languages. The maximum robustness approach instead advocates obtaining independent measures of currently coded languages.

It also makes sense to test whether results are robust when using alternative datasets. That is, does the correlation between humidity and tone hold in both the ANU database and the PHOIBLE database? Since the datasets might not overlap entirely, this is not exactly measurement robustness, but the same principles apply—the more often a correlation is replicated over different sources of data, the more certain we can be that the correlation is meaningful. The sections below test whether this is the case for humidity and tone. The measurements of humidity are also not guaranteed to be totally valid, since they are based on climate models for which there are alternatives, but we do not address this in this paper.

Studies that control for linguistic history also make assumptions about the historical relatedness of languages, most basically which language family a language belongs to. The Glottolog database (Hammarström et al., 2017) is emerging as the leading authority on this, and is particularly useful because it has an explicit set of assumptions behind its classification. However, other classifications exist, and some studies run statistical tests using alternative classifications to check that the result remains similar (e.g., Torreira et al., 2014). Cross-linguistic analyses also make assumptions about the identity of languages. For example, identifier codes are used to link data between databases (ISO code, Glottocodes). Different sources can disagree about the identification of a particular variety, or have errors in matching. Identifying errors and robustness is difficult, but one approach might be to cross-reference the identifier codes with independent measures of their geolocation. Some languages are spoken over large areas and there are justified disagreements on where to place point locations, but the majority of languages are small and well represented by a point. For example, when matching up languages in the ANU phonotactics database with those in the PHOIBLE database, the distance between the stated geographic coordinates is below 500 km for over 95% of languages (2% of languages differed by more than 1,000 km, which might represent problems).

### 4.2. Structural robustness

Most of the robustness tests in macro economics papers relate to whether the main result of interest still holds under a range of controls for potential confounding factors, what is referred to as a *sensitivity analysis*. This kind of robustness is most closely related to structural robustness (Weisberg and Reisman, 2008), since it relates to the structure of the statistical model. Identifying the relevant control variables is not easy. Procedures such as systematic literature reviews help to identify potential confounding variables in a systematic manner (see Bero et al., 1998; Khan et al., 2001; Liberati et al., 2009). The causal graph approach above aims to help this process, particularly in identifying variables that do not need to be controlled for. This process is also becoming easier with the rise of meta-databases of statistical results such as *Metalab* (Lewis et al., 2015) and the *Explaining Human Cultures* database (Ember, 2016b). An database of causal links in evolutionary linguistics is currently in development (Roberts, 2018).

Minimally in cross-linguistic research a control for historical influence is needed. For example, it is now standard to use a language's family as a random effect in regression models, and many papers use geographic areas as a control for horizontal contact. In order to apply certain controls, it may be necessary to implement different methods. Table 1 summarizes the tests done on the correlation between future tense and economic decisions in Roberts et al. (2015). Inclusion of different control variables affects whether the correlation is significant, suggesting that it is an artefact of historical processes (see also Mavisakalyan and Weber, 2017 for a wider review of studies).

**Table 1:** Summary of the statistical tests in Roberts et al. (2015) relating future tense to economic choices.

**Test**	**Data aggregated?**	**Controls**			**Correlation significant?**
		**Language family**	**Geographic area**	**Country**	
Mixed effects model	No	Yes	Yes	Yes	No
Regression on matched samples	No	Yes	No	Yes	Yes
Serendipity test	No	Yes	No	Yes	Yes
Independent samples	Yes	Yes	No	No	Yes
Partial Mantel test	Yes	Yes	Yes	No	Yes
Partial Stratified Mantel test	Yes	Yes	Yes	No	No
Geographic autocorrelation	Yes	No	Yes	No	Yes
Phylogenetic Generalized Least Squares	Yes	Yes	No	No	Yes
PGLS within families	Yes	Yes	No	No	No

Further options in statistical analyses could affect a result. For example, in mixed effects modeling there are different approaches to testing for significance, including comparing the overall fit of nested models (with either “forwards” or “backwards” comparison) or looking at the estimations of a coefficient within a full model (see Roberts et al., 2015 for a comparison). There is little agreement on these, and best practices appear to differ by discipline. Even the most sensible random effects structure is often debated (Barr et al., 2013; Bates et al., 2015).

Woodward (2006) and Hoover and Perez (2004) note that ensuring structural robustness is a hard problem, especially since results may be sensitive not just to the set of control variables, but to the particular combination of control variables, causing an exponential explosion of possible control models. Barth and Kapatsinski (2017) suggest that this is a real problem for linguistics because aspects of language are highly redundant and inter-related. Instead of committing to one “best” model for the final results, Barth and Kapatsinski (2017) suggest a “multimodel inference” approach, which assesses the hypothesized relationship in a wide range of models.

Another option which is becoming more tractable is to give an unbiased statistical model free reign to pick and choose the particular variables that it tests in order to explain the variation in a target variable. There are some methods from machine learning that provide this kind of option. For example, Slonimska and Roberts (2016) predicted that /w/ and /h/ sounds at the start of a turn would be a good predictor that the next turn would be a question in a corpus of English conversation (because many interrogative words start with /w,h/). Instead of testing the proportion of questions that begin with /w,h/ vs. ones that do not, Slonimska and Roberts (2016) allowed a decision tree algorithm to divide the full set of phonemes in English in any combination that best predicted the distribution of questions. In line with the author's predictions, and in spite of a large number of other possibilities, the tree found that separating turns beginning with /w,h/ from the rest was an efficient way of identifying questions. This essentially provides an unbiased (or at least sociologically unbiased) approach to the hypothesis and expands the space of alternative hypotheses considered, without facing a combinatorial explosion.

### 4.3. Methodological robustness

Weisberg and Reisman (2008) discuss the notion of *structural robustness* in modeling: if the same core components cause the same result across a range of alternative models, then the results are robustly due to those core components. Irvine et al. (2013) extend this notion to include the ability to compare results from models and lab experiments: abstract computational models allow precise specification and transparency, but the representation of cognition may not be realistic. In contrast, lab experiments with human participants use realistic cognition (real human brains), but the precise mechanisms are not transparent. However, if the same results are observed across the two methods, then we can conclude that the core causal components that are shared between the models are robustly responsible for the result. We can extend this further to apply to a wider range of methods, what might be called *methodological robustness*: if the same result is obtained from a wide range of methodological approaches (models, lab experiments, corpus studies, etc.), then we can be increasingly certain that the result is not due to the particular assumptions of a given method. For example, the cultural evolution of compositional structure in language as a product of pressures for compression and expression in iterated learning has been demonstrated in computational models and lab experiments (see Irvine et al., 2013; Kirby et al., 2015). In another example, Slonimska and Roberts (2016, 2017) use cross-cultural typology, corpus analyses, and psycholinguistic experiments to provide robust evidence for the idea that forms of interrogative words adapt to the pragmatic requirements of conversation.

Methodological robustness depends on there being a general theory that can produce hypotheses for many particular cases. For example, the hypothesis regarding humidity and tone may derive from a more general theory about how humidity affects vocal production. The more general theory can produce a range of hypotheses such as communities in drier climates using fewer vowels, or individual speakers using different tones in different parts of the year. Later sections of this paper discuss some concrete ways to test the hypothesized link between humidity and language using a wider range of methods.

### 4.4. Representational robustness

Weisberg and Reisman (2008) also discuss *representational robustness*: whether a result holds when a computational model represents particular aspects using a different representational schema. The most obvious application is to test whether the same conceptual model provides the same results when implemented in two different programming languages. If so, we can be more confident that the result is not due to a particular intricacies of a particular programming language. Roberts et al. (2015) found that results could differ substantially between running the stats on different operating systems, due to small bugs in the code for the *lme4* package (since fixed, see Roberts et al., 2015). Representational robustness is often sought when the methods become complicated in order to ensure that the procedures are correct. For example, Everett et al. (2016b) implemented the statistical tests in both R and Python. Similar results suggest that there were no procedural errors in either. However, this may be better thought of as kind of check on the validity of an analysis in these cases, rather than a check of robustness.

### 4.5. Estimation robustness

All statistical analyses make some assumptions about the statistical procedure. However, robustness does not relate to many assumptions such as the normality of the data because that can be verified for a particular dataset. Instead, robustness relates to assumptions which we cannot verify or for which decisions are somewhat arbitrary. This relates to choices like the statistical framework that is chosen and the particular optimizer used to estimate the coefficients in a regression model. These issues are most similar to the concept of parameter robustness, though that relates to parameters of the mechanistic model. Therefore, this kind of robustness may be termed *estimation robustness*: invariance of the results to assumptions about the statistical estimation. For example, Roberts et al. (2015) compared the results of the same model structure with different kinds of assumptions in the estimators (linear mixed effect models in *lme4*, Bates et al., 2011; Bayesian mixed effect models in *blme*, Dorie, 2011) and demonstrated that results differed considerably, suggesting that the correlation was not robust. Little attention is paid to whether results are robust to changes in the optimizer algorithm within a particular framework. This is mostly justified, since it is unlikely to make a difference, but explicit testing is also possible. The analyses in study 3 below use two different mixed effects modeling frameworks and seven different optimizer algorithms to demonstrate the robustness of the result.

### 4.6. Robustness against *ad hoc* hypotheses

The age of large-scale databases and cheap computation has some dangers: it is easy to test a wide range of relationships between variables without having an *a priori* theory which would predict it. It would be possible to search for strong correlations and then invent an *ad hoc* hypothesis to suit them. Alternatively, researchers may come across a strong correlation by chance and focus their research on it, when a wider view of the domain would have lead them to test different hypotheses. The origin story of the link between lexical tone and a particular genotype may be such a case (Dediu and Ladd, 2007). How can we make sure that a result is robust to this fallacy? Obviously, transparency and honesty apply, but these are not exact methodologies. One approach taken by Dediu and Ladd (2007) and also by Roberts et al. (2015) is to assume that the relationship between the two variables of interest should be stronger than the relationship between one of them and a set of other variables that could have been considered (a “serendipity test”). Roberts et al. (2015) tested whether economic decisions were more strongly correlated with future tense than with any of the other variables in the World Atlas of Language Structures. This is kind of the opposite of controlling for multiple comparisons: controlling for the tests *which could have been done*. In both of the publications, other correlations were reliably weaker, providing evidence that pursuing the hypothesis may be productive.

### 4.7. Combining the causal, incremental, and robust approaches

Combining the approaches from the sections above provides the specification for a maximum robustness approach. The causal structure of hypotheses should be explicitly defined using causal graphs. This should point the way to the next most useful analysis. This analysis should tackle a sub-part of the causal graph with the most appropriate method. The analysis should not aim to definitively prove or disprove the hypothesis, but provide incremental evidence for or against it. Individual studies should attempt to demonstrate at least structural robustness and estimation robustness. Reviewing evidence from multiple studies and multiple methodologies will contribute toward methodological robustness (and maybe measurement and *ad hoc* robustness).

The disadvantage of this approach is that it is unclear how to assess theories when evidence from different studies does not agree. For example, when discussing sensitivity (structural robustness), Leamer (1985) suggests an “extreme bounds” approach: the correlation should be considered non-significant if it is not significant in any single test. Sala-i Martin (1997) points out that, given the massive number of possible control tests, this is too strict, and suggests a threshold for significance such as 95% of tests being significant. The aim is not to try to break the correlation in order to disprove it, but to break the correlation in order to learn more about why it is observed.

In the next sections, I try to apply this approach to the link between humidity and tone, particularly regarding measurement, methodological, and estimation robustness.

## 5. Testing the robustness of the link between humidity and tone

Given the discussion above, one of the most pressing issues for the link between humidity and tone is the robustness of the initial statistical correlation. In the next few sections, I present some replication studies with an alternative dataset, and also some hypothetical future studies that could address some of the specific causal links. Study 1 replicates the initial correlation between humidity and tone from Everett et al. (2015) using an alternative dataset. Study 2 looks at a continuous measure of tone. Study 3 extends Everett (2017)'s study of humidity and vowel use using two phonological datasets and one phonetic dataset. All data, analysis scripts and results are available in an online repository: https://github.com/seannyD/HumidityToneReplication.

### 5.1. Study 1: replication of percentile test with alternative dataset

The statistical tests from Everett et al. (2015) (and further refined in Everett et al., 2016b) used linguistic data from the ANU phonotactics database. These tests can be replicated using measures of tone from the PHOIBLE database (Moran et al., 2014). Data on the number of tones for 1,100 languages was obtained from PHOIBLE and linked to the humidity data from Everett et al. (2015) (several sources in PHOIBLE such as UPSID do not code tone, and these were excluded). As in Everett et al. (2015) languages were divided into complex (three or more tones) and non-complex (two or fewer tones) languages.

**Figure 3:**
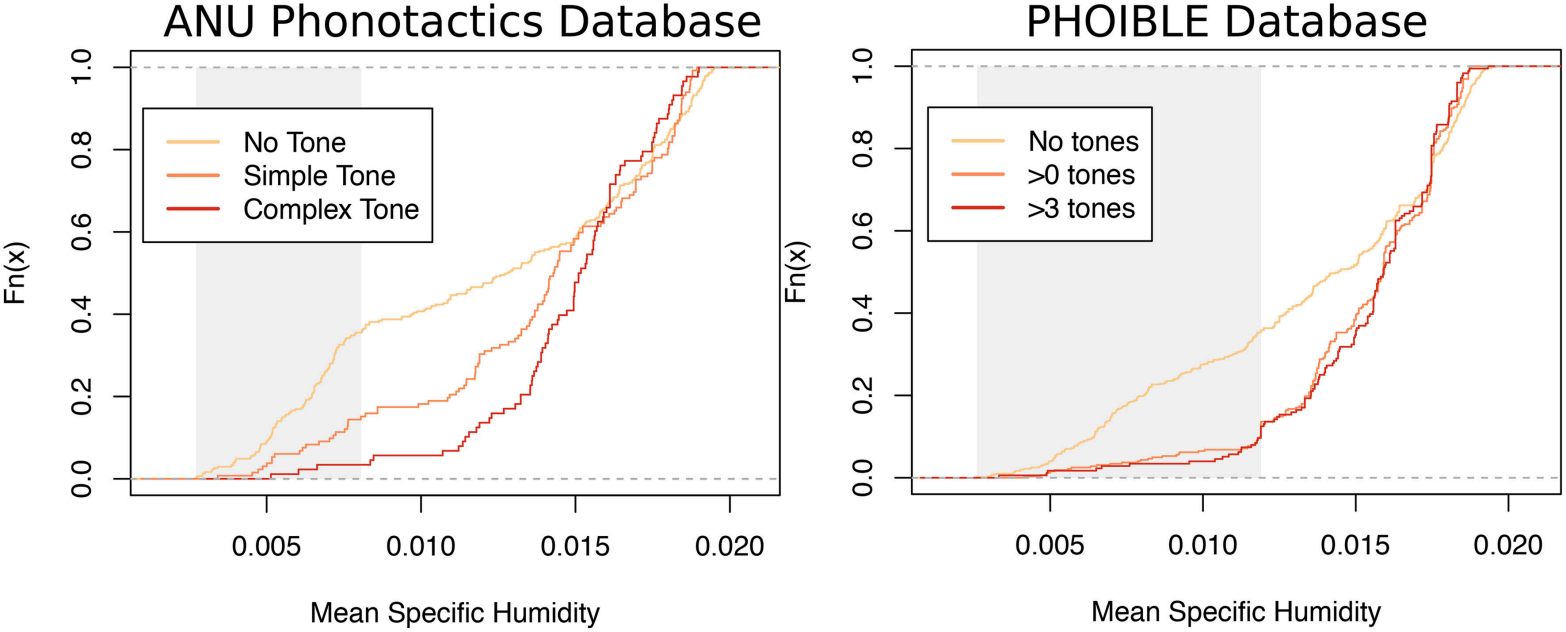
The cumulative distribution of humidity for different categories of language (no tones, <3 tones, 3 or more tones) from the ANU phonotactics database **(left)** and the PHOIBLE database **(right)**. Shaded areas represent the bottom quartile of the humidity distribution (25th percentile).

Figure 3 shows the data from the two linguistic databases side-by-side. There are some differences, but the main pattern is the same: languages with no tones are more frequent at lower humidities than languages languages without tone, and the distributions are more similar in the more humid region. This difference in dry regions only presents a problem for statistical methods which test for a difference in means (see Blasi and Roberts, 2017 for a discussion), which is why an alternative test was formulated.

The procedure for what was called “test 3” in Everett et al. (2016b) tests whether the size of the difference in the 25th percentile of humidity between a sample of complex tone languages and non-complex tone languages is greater than a baseline sample of languages. Sampling is done so that languages are independent in both language family and area. This is an improved version of the test in Everett et al. (2015) based on suggestions by Hammarström (2016).

1. Sample humidity measures from all languages such that no language overlaps in language family nor area. Call this group *R*.
2. From all languages with complex tone, sample humidity measures from languages such that no language overlaps in language family nor area. Call this group *C*.
3. From all languages with non-complex tone, sample humidity measures from languages such that no language overlaps in language family nor area. Call this group *N*.
4. Make sure the groups are the same size (randomly throw out items from the larger groups).
5. For a given percentile *x*, work out the humidity percentile for each group (the humidity below which *x*% of the data lie).
6. Return the difference in percentiles: *C* − *N* and *C* − *R*.
7. Repeat steps 1–6 for 5,000 times.
8. Test the proportion of times (*C* − *N*) > (*C* − *R*).

Table 2 shows the results. In the original study, there were two crucial results: first, that the difference between humidity percentiles was larger than the baseline in more than 95% of samples for the lowest humidity percentile (15th percentile). Secondly, that this value was much lower for higher percentiles (50th and 75th). Neither of these results holds when using the PHOIBLE data.

**Table 2:** Results from the percentile test (test 3) using data from the ANU phonotactics database (from Everett et al., 2016b) and from PHOIBLE (this paper).

**Source**	**Percentile**			
	**15th**	**25th**	**50th**	**75th**
ANU Phonotactics database	0.977	0.818	0.0368	0.113
PHOIBLE	0.855	0.906	0.866	0.715

*Numbers represent proportion of samples in which the size of the difference in humidity quantiles between complex and non-complex tone languages was bigger than between complex languages and a random sample of languages*.

### 5.2. Study 2: using continuous measures of tone

**Figure 4:**
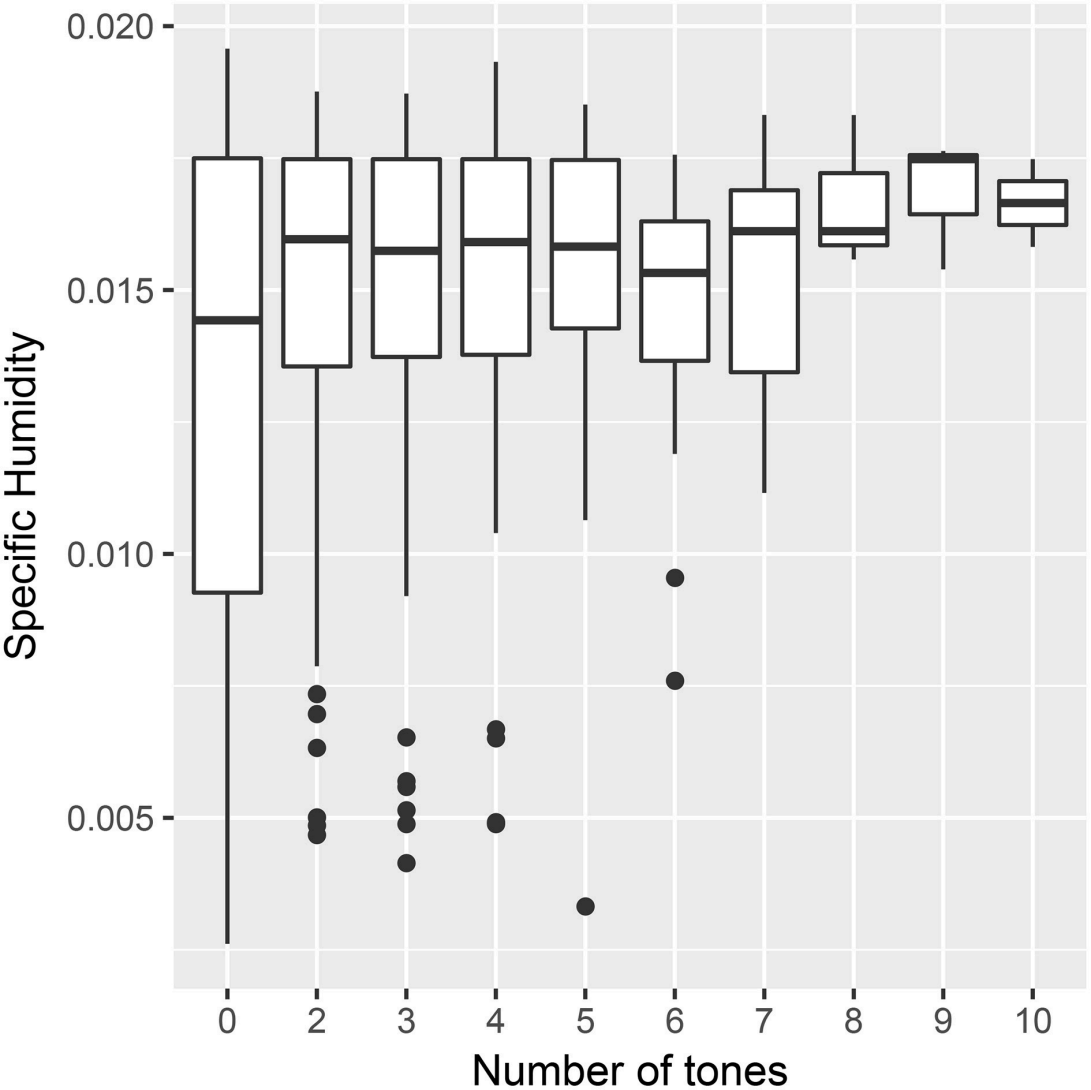
Mean specific humidity by the number of tones in a language from the PHOIBLE database.

The original tests split the data into complex and non-complex tone languages. A continuous variable allows an analysis to predict the number of tones directly. Figure 4 shows the distribution of humidity by continuous tones.

Mixed effects models in the *lme4* package (Bates et al., 2011) for R (R Core Team, 2011) were used to predict the raw number of tones (see Supplementary Material 1). A poisson distribution was used to capture the discrete and skewed nature of the data. The model had random intercepts for language family and geographic area and random slopes for the effect of humidity for both family and area. Including humidity as a fixed effect in the model did not significantly improve the fit of the model (β = 0.19, log likelihood difference = 0.22, df = 1, χ^2^ = 0.45, *p* = 0.50). The same model was tested using the *MCMCglmm* package for R (Hadfield, 2010), which converges on estimates using a Bayesian Monte Carlo Markov chain. This approach is better able to detect multiple conflicting solutions to the fixed effect estimates. The results broadly agreed with those from the *lme4* model (β = 0.20 [−0.04,0.44], *p* = 0.11).

We can dig deeper into the model to try to understand why this relationship is not significant. Table 3 shows how the estimate of the coefficient for humidity changes when removing particular parts of the random effect structure. The estimate is similar when removing the random slope for humidity by language family, suggesting that the effect of humidity does not differ much between families. On the other hand, the estimate is more significant when leaving out the either the intercept or slope by geographic area. Rather than arguing about which result is more valid, we should instead see these differences as suggesting something about the structure of the data. In this case, it suggests that the relationship between tone and humidity is not robust to controls for historical relationships, and in particular confounded by areal effects. This would fit with criticisms which suggest that borrowing is an important confound (Collins, 2016; Winter and Wedel, 2016). In robustness terms, the result is not structurally robust: the correlation does not survive controlling for the confound of contact.

**Table 3:** How the estimate for the coefficient for the effect of humidity on the number of tones changes when altering the random effects structure (*lme4* model with PHOIBLE data).

**Model**	**Estimate**	**Std. Error**	***z*-value**	**Pr(>|z|)**
Full model	0.19	0.28	0.68	0.495
No family intercept	0.75	0.41	1.83	0.067
No family slope	0.19	0.28	0.68	0.495
No area intercept	0.52	0.19	2.70	0.007
No area slope	0.16	0.05	3.43	0.001

#### 5.2.1. Measurement robustness for tone

The results differ considerably in the alternative dataset, mainly because of measurement disagreements. The two sources overlap on 667 languages (Glottolog codes). The correlation in number of tones is only moderate (Cohen's weighted κ = 0.61, *r* = 0.62, see Supplementary Material 2). When categorising languages into those having tones and those having no tones, the databases agree 82% of the time (Cohen's κ = 0.64, “moderate” agreement according to Landis and Koch, 1977, similar results comparing two or fewer tones to three or more). On average, the ANU database predicts a greater number of tones than in PHOIBLE.

PHOIBLE has many sources, but few languages are coded in more than one, making measurement robustness difficult to assess. Where it is possible to measure agreement between these sources, in one case it is very low (AA vs. GM: Cohen's weighted κ = 0.08, *r* = 0.05, *n* = 36) and in another it is very high (SPA vs. UZ: Cohen's weighted κ = 0.95, *r* = 0.95, *n* = 26). We can compare this to the agreement between vowels, which sits between these two extremes (AA vs. GM: Cohen's weighted κ = 0.56, *r* = 0.52, *n* = 36; SPA vs. UZ: Cohen's weighted κ = 0.53, *r* = 0.53, *n* = 26). The differences might be due to differences in methodological approaches, theoretical background or errors in data entry or coding of languages, but are most likely to be due to the inherently difficult nature of quantifying a phonetic system (e.g., dealing with length, nasalisation, diphthongs, see Maddieson, 2013; Moran, 2016 or a specific case e.g., Montes Rodriguez, 2004, p. 111). It is worth noting that PHOIBLE and many other recent databases provide features for continuous and centralized updating and refining of the data by members of the research community through github (e.g., see PHOIBLE's issue tracker: https://github.com/phoible/dev/issues), so these problems will hopefully decrease as time goes on.

The low measurement robustness for the number of tones is concerning, especially since another independent source is hard to produce. However, it is only problematic for the statistical inquiries of this paper if the differences are *biased according to humidity*. This was tested by trying to predict the difference between the two estimates using a mixed effects model with random intercepts for language family and geographic area. If the differences are completely unbiased, then the random effects should not account for a significant proportion of the variance. This was the case (for family *p* = 0.09; for area *p* = 1, see Supplementary Material 2). If estimates differ in particular humidity conditions, then a fixed effect of humidity should improve the fit of the model. This was not the case (*p* = 0.42). Therefore, the differences between the sources are not biased with regards to language, area, or humidity. Given the results above, however, it is still clear that the different sources lead to different results regarding the link between tone and humidity. This is another reason to take a maximum robustness approach.

## 6. Study 3: humidity and proportion of vowels

This section tests the robustness of the link between humidity and vowels. Everett (2017) looked at the proportion of vowels vs. consonants in basic wordlists from the ASJP database (Wichmann et al., 2013). This was used as a measure of the relative frequency of vowels and consonants during speech, and it was shown that this correlated with the specific humidity of the areas in which the languages were spoken. In this section, a different approach is taken: to try and predict the proportion of vowels in a language's phoneme inventory by humidity. The relative frequency of phonemes is a more ideal measure (indeed, Everett argues that phoneme inventories are misleading since it is habitual use that is more important). However, the basic word lists used in the study are relatively restricted, and the theory could extend to affecting the number of distinctions in the phoneme system. In any case, the study here is an illustrative example of expanding the range of analyses.

Data on phoneme inventories was taken from the PHOIBLE database (Moran et al., 2014). As above, a linear mixed effects model (in package *lme4*) was used to predict the ratio of vowels to consonants within a language's inventory by humidity (see Supplementary Material 3). Since the vowel ratio may be affected by the total phoneme inventory size, it was added as a fixed effect. Adding humidity as an additional fixed effect significantly improved the fit of the model (β = 0.17, log likelihood difference = 3.9, df = 1, χ^2^ = 7.77, *p* = 0.005), indicating that higher humidity was associated with a greater proportion of vowels. Interestingly, there was a significant interaction between humidity and inventory size (β = 0.10, log likelihood difference = 9.3, df = 1, χ^2^ = 18.57, *p* < 0.001), such that the correlation between proportion of vowels and humidity is stronger for languages with larger inventories.

We can test the estimation robustness of the finding. The estimates did not change much when using 6 alternative optimizers, providing at least some estimation robustness (see Supplementary Material 2). The coefficient estimates are also very similar when using the *MCMCglmm* package. There was a significant effect of humidity (β = 0.16 [0.05,0.28], *p* = 0.004) and a significant interaction between humidity and inventory size is also significant (β = 0.10 [0.05,0.17], *p* < 0.001). In this model, there is also a significant main effect of inventory size (0.09 [0.03, 0.16], *p* = 0.003).

The effect size is very small. The model predicts that when comparing the language in the driest environment to the language in the most humid environment that the proportion of vowels should increase from about 25% to about 35%. In a language with an average phoneme inventory size, that's a difference of about 3 vowels.

### 6.1. Measurement robustness for the relative frequency of vowels

Everett (2017) used the proportion of vowels in the basic vocabulary lists of the ASJP database as a proxy for the relative frequency of vowels in general speech. The ASJP database contains a large number of languages (over 7,000 varieties), but a small number of concepts (most languages have 40, some have 100). Are the estimates robust when increasing the number of concepts? An alternative could be the database of lexical items compiled by Slonimska and Roberts (2017) from sources in Haspelmath and Tadmor (2009); Key and Comrie (2015) and Borin et al. (2016). It has 999 concepts in 226 languages (about 10 times more concepts but 10 times fewer languages compared to the ASJP). The correlation between the proportion of vowels in the ASJP and the alternative dataset is reasonably good (*r* = 0.65). However, the magnitude of the differences between the two measures varies significantly between language families, geographic areas and (weakly) according to humidity. That is, the ASJP estimates of vowel frequency are biased (unlike the estimates for tones). It is not clear what the next course of action here is. The alternative dataset does not have enough languages to reliably detect the original correlation, but a larger database with many more concepts is unlikely to appear soon (though see the upcoming Lexibank database, http://glottobank.org/#lexibank). In this case, it may be best to turn to other measures. For example, phonetic measures may be more reliable because there are objective, repeatable methods.

### 6.2. Using phonetic measurements

**Figure 5:**
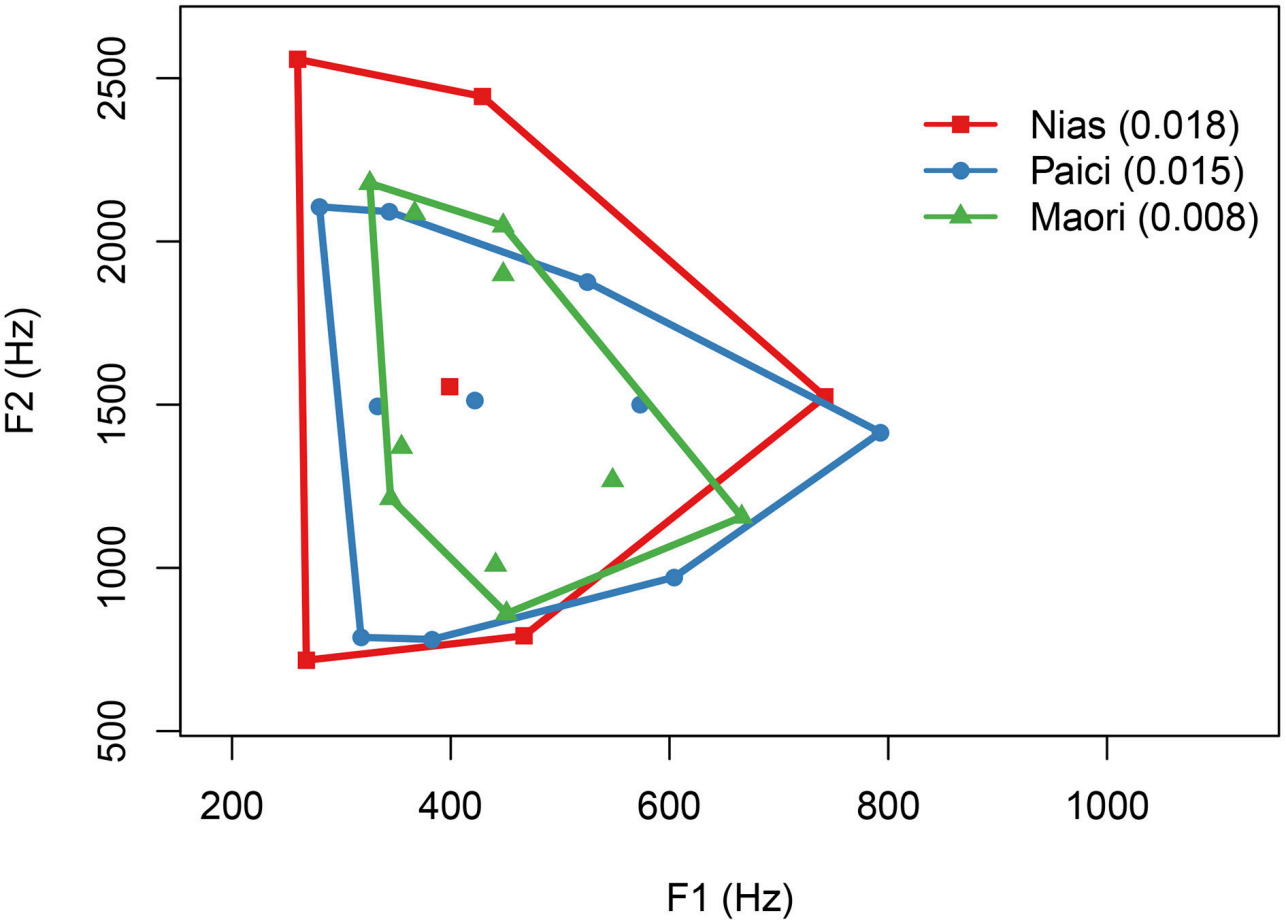
An example of the range of vowel systems from three Austronesian languages. The humidity in which the languages are spoken are shown in parentheses next to the language name in the legend.

It is also possible to use phonetic measurements to test the hypothesis (see also Maddieson, under review in this volume). Indeed, it might be easier to automatically extract and replicate phonetic measurements (Ennever et al., 2017). The hypothesis predicts that speakers in drier climates would use a more restricted range of frequencies in vowels. Becker-Kristal (2010) provides a database of phonetic measurements of vowels for many languages based on a meta-analysis. Following Weirich and Simpson (2014), for each language, the F1 and F2 measures of all vowels in a language were taken, then the area of the convex hull of the points was calculated. This represents the range that a vowel system takes up (see Figure 5).

**Figure 6:**
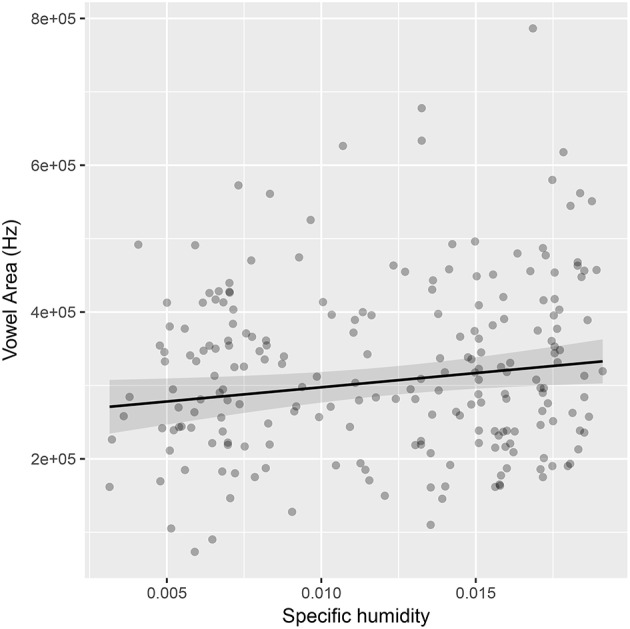
The relationship between the range of frequencies a vowel space covers and the specific humidity in which it is used. The regression line is drawn according to the mixed effects model estimates.

The area of 219 vowel systems was calculated. A mixed effects model was used to predict vowel area with random intercepts for language family and geographic area. Even when controlling for the number of vowels in a system, adding specific humidity as a fixed effect significantly improved the fit of the model (β = 0.16, log likelihood difference = 2.5, χ^2^ = 5.01, df = 1, *p* = 0.025, see Supplementary Material 4). The effect size was small (see Figure 6), and there was not enough data to include random slopes for specific humidity, so this result is probably not robust. The main point here is that it is possible to use more fine-grained measures from alternative data sources to test large-scale statistical claims and contribute to the methodological robustness of the result.

## 7. Methodological robustness for the effects of humidity on language

The studies above looked at the correlation between humidity and tone or between humidity and vowel use, but these are just the end-points of a more detailed causal chain drawn up in section 2. The maximum robustness approach suggests that each of these links can be addressed with different methods and data. The following subsections suggest some ideas for how this might be done. The point here is not to test each link, but to demonstrate that methods from many different areas of linguistics can be brought to bear on them.

### 7.1. Iterated learning

**Figure 7:**
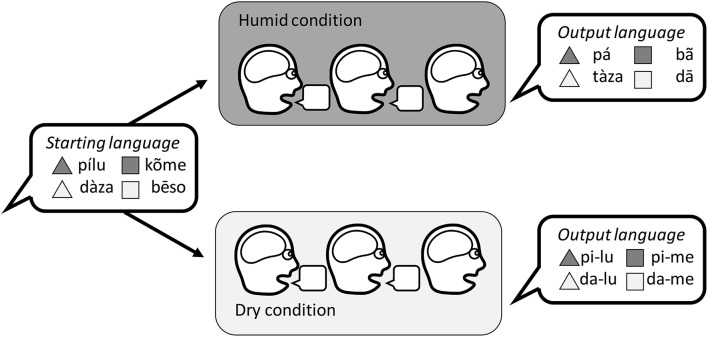
A hypothetical iterated learning experiment to investigate the link between humidity, desiccation, production, and the loss of tone distinctions. The desiccation hypothesis predicts that languages transmitted in a humid condition would develop a (possibly compositional) system that preserved distinctions in tone, while languages in a dry condition would develop a compositional system that relied on non-tonal distinctions.

One could imagine an iterated learning study to address the link between humidity, desiccation, production, and the loss of tones. A participant would learn an artificial language where the labels were auditory words with distinctions in tone and non-tone segments. They would be asked to reproduce the correct labels, and their productions would be given as the input language to a new participant. This process would be repeated to create a chain of generations in which the labels would change gradually. Chains would be run in specially controlled rooms with two conditions: dry air and humid air. The prediction is that distinctions in tone would survive in the humid condition, but be more likely to disappear from the dry condition (Figure 7). Alternative conditions could be tested such as having the participants communicate with a partner using the language, to test the role of miscommunication over and above production error.

### 7.2. Historical case study

**Figure 8:**
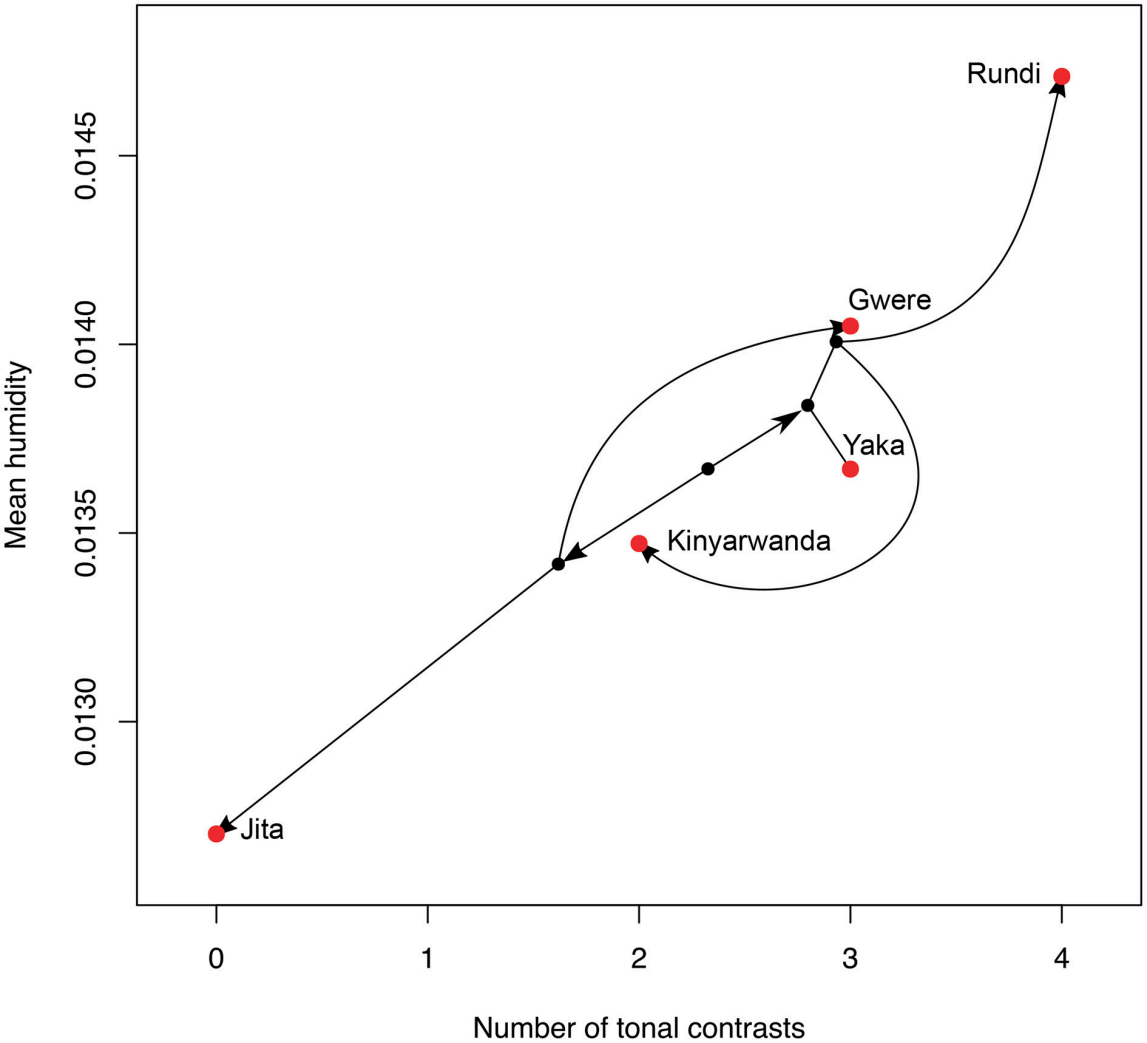
Phylomorpho space plot of Northeast Savannah Bantu languages. Red dots are the actual attested languages, and these are joined with lines to black dots representing their ancestors reflecting the consensus phylogenetic tree from Currie et al. (2013), where the position of ancestor points regarding both number of tonal contrasts and mean humidity are calculated via continuous ancestral state reconstruction. Some of the branches of the tree are altered for clarity, line lengths are not meaningful.

We can use the cross-linguistic data to find promising case-studies for more detailed historical linguistic work. Data on tones and humidity were used together with the historical tree suggested by Glottolog to infer the likely ancestral states in the Atlantic Congo family. The most interesting section is the Narrow Bantu clade, where two sub-groups (Eastern and Central-Western Bantu) enter drier climates. This clade is also known to have generally fewer tonal contrasts and simpler tone systems (e.g., only high vs. low, Güldemann, 2011, p. 115). Crucially, some languages within the sub-groups re-enter humid zones, for instance the languages which border Rwanda and Tanzania. Figure 8 shows the tone and humidity measures for some of these languages, linked by the phylogenetic tree inferred by Currie et al. (2013) (group J, also closely related in Glottolog). The trend is the predicted one—fewer tones occur in drier climates. For example, Jita and Gwere split up, one heading into a humid region, and one heading into a dry region, with the predicted change in tones. The points are also not very clustered by historical relatedness—Yaka and Gwere are similar in tones and in humidity, but actually not close on the phylogenetic tree. We suggest that this group of languages provides an excellent candidate for a more detailed case study of historical changes to tone.

### 7.3. Corpus study: production

Croft's approach to language change is that the locus of change is individual utterances (Croft, 2000, perhaps improved by Tamariz's focus on the reproductions themselves separate from their meanings, Tamariz, 2017a). That is, selection operates on variation in productions turn-by-turn, and not just in generations. The hypothesis linking tone and humidity would predict that, in order for change to happen, there would have to be underlying variation on which selection could operate, and that this should be visible within speech communities. For example, do users of tone languages show variation in the proportion of different tone types that they use, and do they vary systematically with humidity. That is, a language offers a speaker multiple choices about how to express a meaning. These options may differ in the demands they make on vocal fold control, and so may be more or less difficult to produce in different locations or at different times of the year. This could be tested in two ways. First, do speakers of a language such as Cantonese produce tones differently or use a different proportion of tones in the humid parts of China compared to the colder, more arid parts? Secondly, do speakers' productions differ according to the seasonal change in humidity?

Databases with geolocations and dates of production are not common, but the CHILDES database does include the dates of recordings. Data from 189 recordings of Cantonese were obtained from CHILDES (Fletcher et al., 1996, 2000; Lee et al., 1996; Weizman and Fletcher, 2000) and productions by children were removed. The number of each type of tone was calculated for each recording, then linked to the month that the recording was taken. The following prediction was made: contour tones would require more precise control of vocal fold vibration, so would be avoided during the drier months. Mean monthly specific humidity was collected around Hong Kong for the years spanning the corpus collection (Kalnay et al., 1996). Mixed effects modeling with random intercepts for source corpus was used to test whether the contribution of humidity significantly predicted the proportion of use of contour tones (see Supplementary Material 5). There was no significant effect (χ^2^ = 0.28, *p* = 0.59), and in fact the use of contour tones does not vary over the year.

### 7.4. Corpus study: miscommunication

One link in the causal chain relating to interaction predicts that more complex tones are more difficult to produce and therefore more likely to cause problems of understanding. For example, Mandarin has 4 tones, with the 3rd tone being a contour tone with a wide range, possibly requiring more precise control of the vocal folds (though often reduced in speech). One might predict that turns in conversation including 3rd tones would be more susceptible to errors in production and perception, and therefore more likely to elicit repair from interlocutors.

A corpus of repair sequences in Mandarin conversation was obtained from Dingemanse et al. (2015), collected and transcribed by Kobin Kendrick. The proportion of each type of tone was counted in trouble sources (turns followed by open other-initiated repair, indicating a problem of hearing or understanding) and compared to the baseline proportion of each tone type in a wider corpus (Wan and Jaeger, 1998). The 3rd tone was significantly more likely in trouble sources (using a χ^2^ test on the tone counts in Table 4, χ^2^ = 9.89, df = 3, *p* = 0.02). This is in line with the hypothesis, but much more could be done to check the robustness of this claim. In particular, it should be possible to look at tone type usage in the actual source of the problem for restricted repair initiations, rather than the whole prior turn in general. Again, the point here is that specific data and analyses can be brought to bear on particular links in the causal chain.

**Table 4:** Counts (and percentages) of different tonemes in Mandarin in general (baseline from Wan and Jaeger, 1998), and in conversational turns that lead to open repair (from Dingemanse et al., 2015).

**Tone**	**1**	**2**	**3**	**4**
Baseline	46 (23%)	44 (22%)	43 (21%)	71 (34%)
Trouble sources	56 (24%)	34 (15%)	75 (33%)	64 (28%)
Percentage difference	+2	−7	+12	−7

### 7.5. Individual speech

It is also possible to look at whether individual speakers shift the way they speak due to the climate, though a large sample of recordings would be needed. The ideal database would be a few minutes recording every day over the course of several years. This kind of database is rare, but they do exist. For example, Larry King has recorded a show almost every day for over a decade. CNN provides transcripts of these shows from 2000 to 2011 (http://transcripts.cnn.com/TRANSCRIPTS/lkl.html), a total of around 3,500 recordings. These transcripts were downloaded and King's turns were extracted. Personal names and locations were removed and the text was transcribed to a phonological representation using the CMU pronouncing dictionary (Weide, 2005, on average 95% of tokens were transcribable, 91% of types). Daily specific humidity estimates are also available for each show's recording date and location (assuming Los Angeles, Kalnay et al., 1996). We can then test whether King uses a smaller proportion of vowels compared to consonants during drier weather.

**Figure 9:**
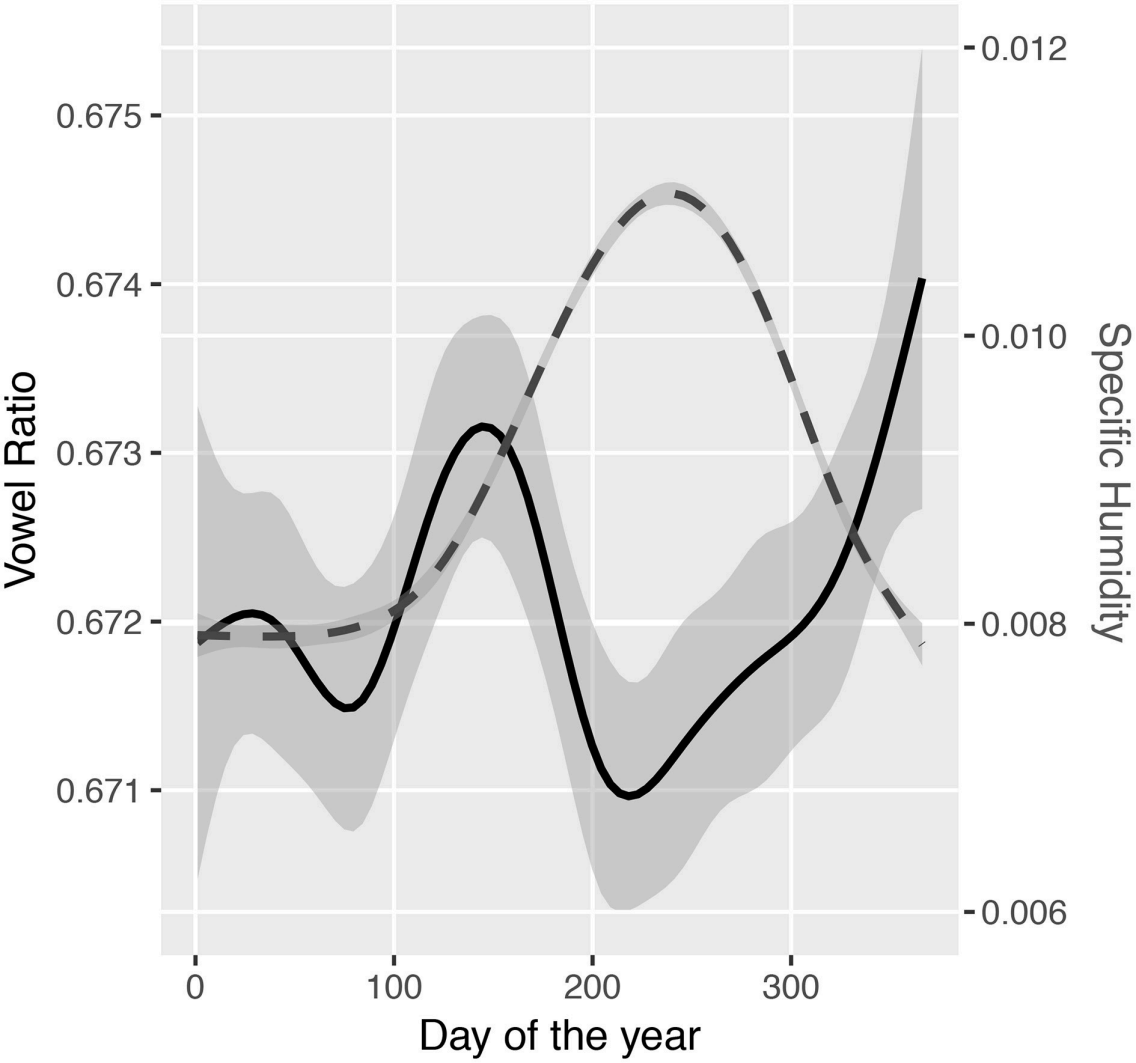
A graph showing the average proportion of vowels used by Larry King (solid line, left axis) and the average specific humidity of Los Angeles (dashed line, right axis) over days of the year. Lines are smoothed using a general additive model with 95% confidence intervals.

King's vowel ratio is very stable. It ranges between 0.63 and 0.70 (more consonants than vowels, sd = 0.008, see Figure 9). A general additive model was used to test the relationship between the vowel ratio and humidity. There was a significant relationship [*F*_(2.85, 3.61)_ = 4.95, *p* = 0.001, see Supplementary Material 6], but higher humidity was associated with the use of proportionally fewer vowels, going against the prediction.

There are, of course, many problems with this study. The recordings are mostly done in air-conditioned studios (in fact, the results are consistent with drier air due to air conditioning during summer), and occasionally broadcast from other cities. It is also probable that seasonal topics contribute to the variation in vowel ratio. However, the point here is that this question is at least empirically approachable.

## 8. Summary of the state of the hypothesis linking humidity and language

The maximum robustness method identified several weak parts in the causal chain relating humidity and tone. These are mostly regarding whether the effect on production is large enough to affect linguistic systems in the long term. Table 5 summarizes the robustness analyses above. The original relationship between tone and humidity was not replicated in an alternative database. Consistent with criticisms that the borrowing is a confounding factor, the strength of the relationship is mostly accounted for by differences in particular geographic areas. The alternative methods had mixed results, but at least demonstrated that the hypothesis can be approached from many different angles.

**Table 5:** A summary of the previous and current results relating humidity to tone and vowels.

**Variable**	**Test**	**Source**		
		**ANU**	**PHOIBLE**	
Tones	Percentile test	Yes	No	
	LME	No	No	
	MCMC	?	No	
	Measurement robustness	Medium	-	
		**ANU**	**PHOIBLE**	**ASJP**
Vowels: lexicon	LME	–	–	Yes
	Measurement robustness	–	–	Low
Vowels: inventory	LME	?	Yes	
	MCMC	?	Yes	
	**Alternative methods**	**Various sources**		
Tones	Iterated learning	?		
	Historical case study	?		
	Corpus study: production	No		
	Corpus study: miscommunication	Yes		
Vowels	Corpus study: individual speech	No		
	Phonetic measurements	Yes		

*Cells represent whether there was a significant relationship*.

The relationship between humidity and vowels is more robust, though the effect size is small. There is positive evidence from relative frequency of vowels in the basic vocabulary, relative frequency in phonemic inventories (with some estimation robustness) and phonetic measures. However, the measurement robustness of the frequency of vowels in the lexicon may be low. It should be noted that Everett (2017) suggests that looking at phoneme inventories is not a valid test of the hypothesis, since usage frequency is more important (and see also Maddieson, under review).

In summary, the effect of humidity on language is an intriguing frontier in accounts of linguistic adaptation. However, the basic correlation between humidity and tone is not robust. It is unlikely that new independent global data on tones will become available soon, so the best next step for this line of research is to diversify the methodological approaches and reach toward experimental and diachronic studies. Having said this, there are other lines of research that are better grounded in linguistic theory and are more likely to represent substantial effect sizes. What is needed is a more detailed mechanistic model of how production is affected by the ecological conditions, and how these effects put a pressure for whole linguistic systems to change.

## 9. Conclusion

This paper discussed the approach to studying how languages adapt to external selective pressures by looking at patterns in large-scale, cross-cultural databases. It advocated a maximum robustness approach, which is empirical, causal, incremental, and robust. Each of these aspects feeds into the others to provide an increasingly clear evidence for a particular hypothesis. This method encourages researchers to move beyond large-scale statistical analyses and into more diverse methods and toward more controlled, causal accounts by breaking a hypothesis down into smaller causal links, then addressing each link with the most appropriate method and data. This approach was illustrated with examples based on the research into the relationship between humidity and tone. Although large-scale statistical approaches can provide the motivation to pursue a hypothesis, and ultimately a demonstration that the whole causal chain produces significant adaptation, it is unlikely that they will be able to provide convincing evidence on their own. Some examples above suggested ways in which other approaches could help, including laboratory phonetics, iterated artificial language learning experiments, corpus studies, and historical case studies.

Engaging with this range of methods and disciplines is difficult for just one researcher. It is more likely that this approach will be successful in the context of collaboration between specialists from different areas. Open access to data and statistical modeling code will also be a key to making these projects viable. It also means that large-scale interdisciplinary grants will become more important, as well as recognising the different types of contribution that authors make to a paper (theoretical, experimental, data collection, statistical, organizational etc.).

The maximum robustness approach advocates doing many analyses with as many sources of data as possible. However, it is important to note that it definitely does not advocate practices such as p-hacking, cherry-picking or presenting *post-hoc* descriptions as a priori hypotheses. Studies with good structural robustness will run many analyses, then *report them all*. The aim is to provide many alternative viewpoints, not to discover the most convenient statistic. Recent advances in meta-analysis methods are providing ways of navigating the range of results (Lewis et al., 2015). The approach is also different from the slow science movement (Lutz, 2012). While both emphasize careful and detailed consideration of theories and methods, the maximum robustness approach is more open and pragmatic. In linguistics, different phenomena are deeply interrelated and new data is a scarce resource. It is better that the analyses are published and discussed so that they can help other research, even if the conditions are not perfect. Indeed, according to the maximum robustness approach, foregrounding the potential flaws and limits of an analysis is useful. On the other hand, while swift publishing is encouraged, this approach does require a more cautious approach to acceptance of theories. Overstated results and hasty adoption may be hard to overturn. The study above linking language to economic behavior (Chen, 2013) was quickly taken onboard by economists and the data has been reused (Santacreu-Vasut et al., 2014; Hicks et al., 2015; Pérez and Tavits, 2017), even though the original findings are now in doubt (Roberts et al., 2015, though see Mavisakalyan and Weber, 2017). The solution may mean that researchers need to spend more time refining the communication of their research, especially to non-specialist audiences. It may also require more moderate language to describe the significance of studies and a full account of its flaws. However, like language, scientific practice adapts to its wider ecology, and the current climate promotes hyperbolic discoveries over statistical grumblings. Wider changes may be necessary to support the maximum robustness method.

## Author contributions

The author confirms being the sole contributor of this work and approved it for publication.

### Conflict of interest statement

The author declares that the research was conducted in the absence of any commercial or financial relationships that could be construed as a potential conflict of interest.
