# Supplementary material for: Robust, Causal, and Incremental Approaches to Investigating Linguistic Adaptation
Source: Front Psychol. 2018 Feb 21;9:166. doi: 10.3389/fpsyg.2018.00166 (PMC5826341; doi:10.3389/fpsyg.2018.00166)

Tone and humidity: PHOIBLE replication

Load libraries

```
library(lme4)
library(sjPlot)
library(caret)
library(car)
library(MCMCglmm)
library(xtable)
```

```
setwd("~/Documents/MPI/ClimateAndLanguage/PHOIBLE_Replication/analysis/")
```

Load data

The PHOIBLE database contains data for 1667 varieites with unique glottolog codes. The data come from several source corpora, only some of which have data on tone languages (GM, PH, RA, SAPHON). These have already been filtered out in the file `phoibleTonesAndHumidity.csv`, and the languages have been linked to geographic coordinates and mean humidity values. There are multiple sources for some languages. PHOIBLE suggests a 'trump' source for each of these cases, which we select here.

```
p = read.csv("../data/phoibleTonesAndHumidity.csv")
p = p[p$Tones!=1,]
```

We transform the humidity variable with a box-cox power transformation, then scale and center the values.

```
pp = preProcess(p[,c('Tones','specH.mean')], method="BoxCox")

p$specH.mean.center = bcPower(p$specH.mean, lambda = pp$bc$specH.mean$lambda)

p$specH.mean.center = scale(p$specH.mean.center)

hist(p$specH.mean.center)
```

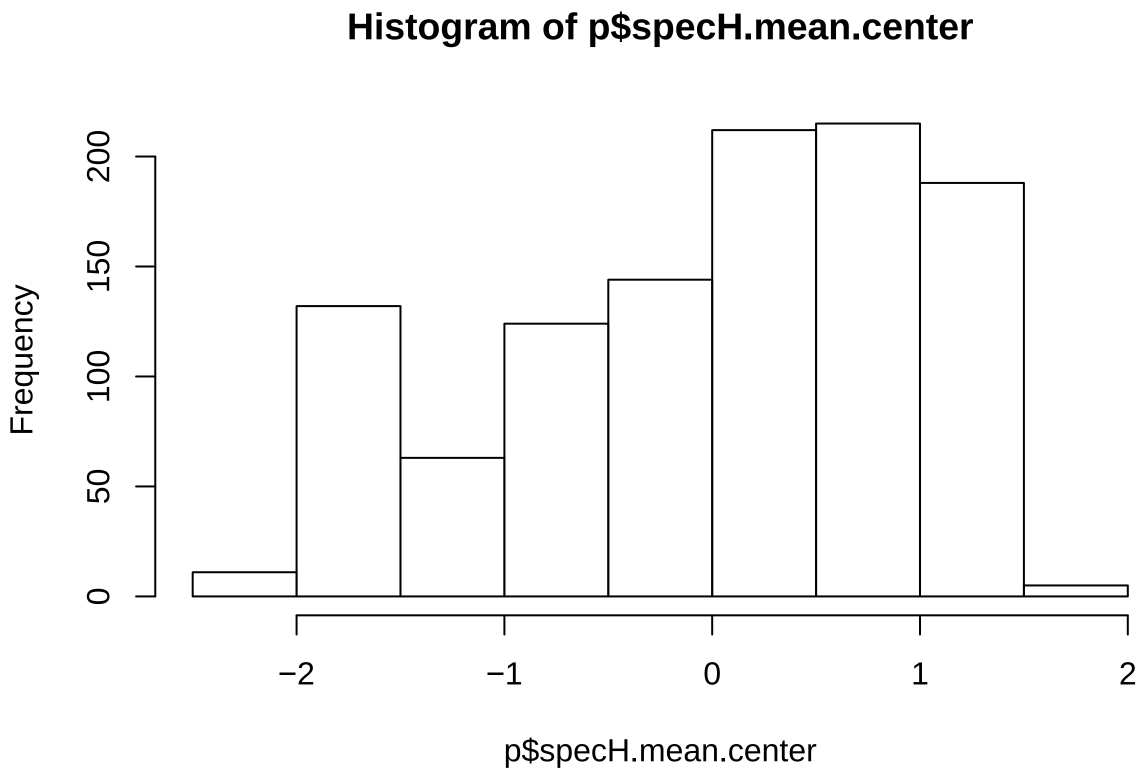

Plot the raw data:

```
gx = ggplot(p, aes(x=as.factor(Tones), y = specH.mean)) + geom_boxplot() +
  xlab("Number of tones") + ylab("Specific Humidity")
gx
```

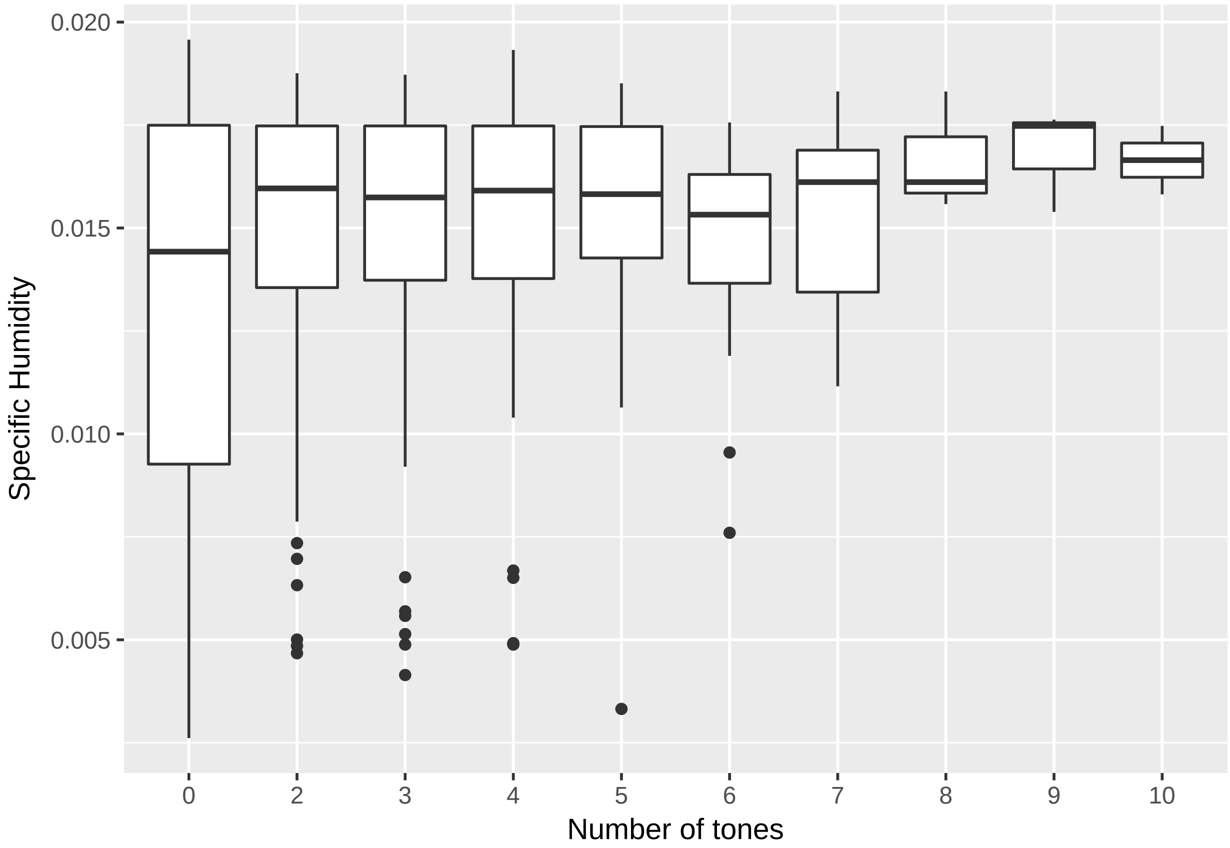


```
# Write to file
pdf("../results/PHOIBLE_Tones_raw.pdf", width=4, height=4)
gx
dev.off()
```

```
## pdf
##   2
```

Mixed effects modelling using lme4

We run mixed effects models predicting the number of tones (`Tones`) using a poisson model to capture the discrete and skewed nature of the data. We start by building a null model with only random effects for language family (`Family`) and geographic area (`autotyp.area`).

Test the contribution of random slopes:

```
m0 = glmer(Tones~1  + (1|Family) +
             (1|autotyp.area),
           data=p, family=poisson,
           control = glmerControl(optimizer = 'bobyqa'))

m1 = glmer(Tones~1  + (1|Family) +
             (1+specH.mean.center|autotyp.area),
           data=p, family=poisson,
           control = glmerControl(optimizer = 'bobyqa'))

m2 = glmer(Tones~1  + (1+specH.mean.center||Family) +
             (1+specH.mean.center|autotyp.area),
           data=p, family=poisson,
           control = glmerControl(optimizer = 'bobyqa'))

anova(m0,m1,m2)
```

```
## Data: p
## Models:
## m0: Tones ~ 1 + (1 | Family) + (1 | autotyp.area)
## m1: Tones ~ 1 + (1 | Family) + (1 + specH.mean.center | autotyp.area)
## m2: Tones ~ 1 + (1 + specH.mean.center || Family) + (1 + specH.mean.center |
## m2:     autotyp.area)
##    Df    AIC    BIC  logLik deviance Chisq Chi Df Pr(>Chisq)
## m0  3 3103.9 3118.9 -1549.0   3097.9
## m1  5 3086.5 3111.5 -1538.3   3076.5 21.37      2  2.288e-05 ***
## m2  6 3088.5 3118.5 -1538.3   3076.5  0.00      1     0.9983
## ---
## Signif. codes:  0 '***' 0.001 '**' 0.01 '*' 0.05 '.' 0.1 ' ' 1
```

Random slopes for language family signficantly improve the model, white random slopes for area do not. However, we know that there are likely to be random effects by area, so we include these in the model.

Run a model with a fixed effect of humidity:

```
m3 = glmer(Tones~1 +specH.mean.center  +
             (1+specH.mean.center||Family) +
             (1+specH.mean.center|autotyp.area),
           data=p, family=poisson,
           control = glmerControl(optimizer = 'bobyqa'))
```

Test the contribution of humidity by comparing a model with and without humidity as a fixed effect:

```
anova(m2,m3)
```

```
## Data: p
## Models:
## m2: Tones ~ 1 + (1 + specH.mean.center || Family) + (1 + specH.mean.center |
## m2:     autotyp.area)
```

```
## m3: Tones ~ 1 + specH.mean.center + (1 + specH.mean.center || Family) +
## m3:     (1 + specH.mean.center | autotyp.area)
##    Df    AIC    BIC  logLik deviance  Chisq Chi Df Pr(>Chisq)
## m2  6 3088.5 3118.5 -1538.3   3076.5
## m3  7 3090.1 3125.1 -1538.0   3076.1 0.4471      1     0.5037
```

```r
summary(m3)
```

```
## Generalized linear mixed model fit by maximum likelihood (Laplace
##   Approximation) [glmerMod]
##  Family: poisson  ( log )
## Formula:
## Tones ~ 1 + specH.mean.center + (1 + specH.mean.center || Family) +
##     (1 + specH.mean.center | autotyp.area)
##    Data: p
## Control: glmerControl(optimizer = "bobyqa")
##
##      AIC      BIC   logLik deviance df.resid
##   3090.1   3125.1  -1538.0   3076.1     1087
##
## Scaled residuals:
##     Min      1Q  Median      3Q     Max
## -2.0202 -0.4721 -0.1780  0.2530 10.6700
##
## Random effects:
##  Groups       Name              Variance Std.Dev. Corr
##  Family       (Intercept)       3.2284   1.7968
##  Family.1     specH.mean.center 0.0000   0.0000
##  autotyp.area (Intercept)       2.5514   1.5973
##               specH.mean.center 0.3109   0.5576   0.31
## Number of obs: 1094, groups:  Family, 119; autotyp.area, 24
##
## Fixed effects:
##                   Estimate Std. Error z value Pr(>|z|)
## (Intercept)        -2.4628     0.5502  -4.476  7.6e-06 ***
## specH.mean.center   0.1883     0.2760   0.682    0.495
## ---
## Signif. codes:  0 '***' 0.001 '**' 0.01 '*' 0.05 '.' 0.1 ' ' 1
##
## Correlation of Fixed Effects:
##             (Intr)
## spcH.mn.cnt 0.378
```

Plot the model effects:

```r
x = sjp.glmer(m3, 'eff',
             vars=c("specH.mean.center"),
             show.scatter = T, show.ci = T,
             prnt.plot = F)
x[[1]]+ xlab("Scaled humidity")
```

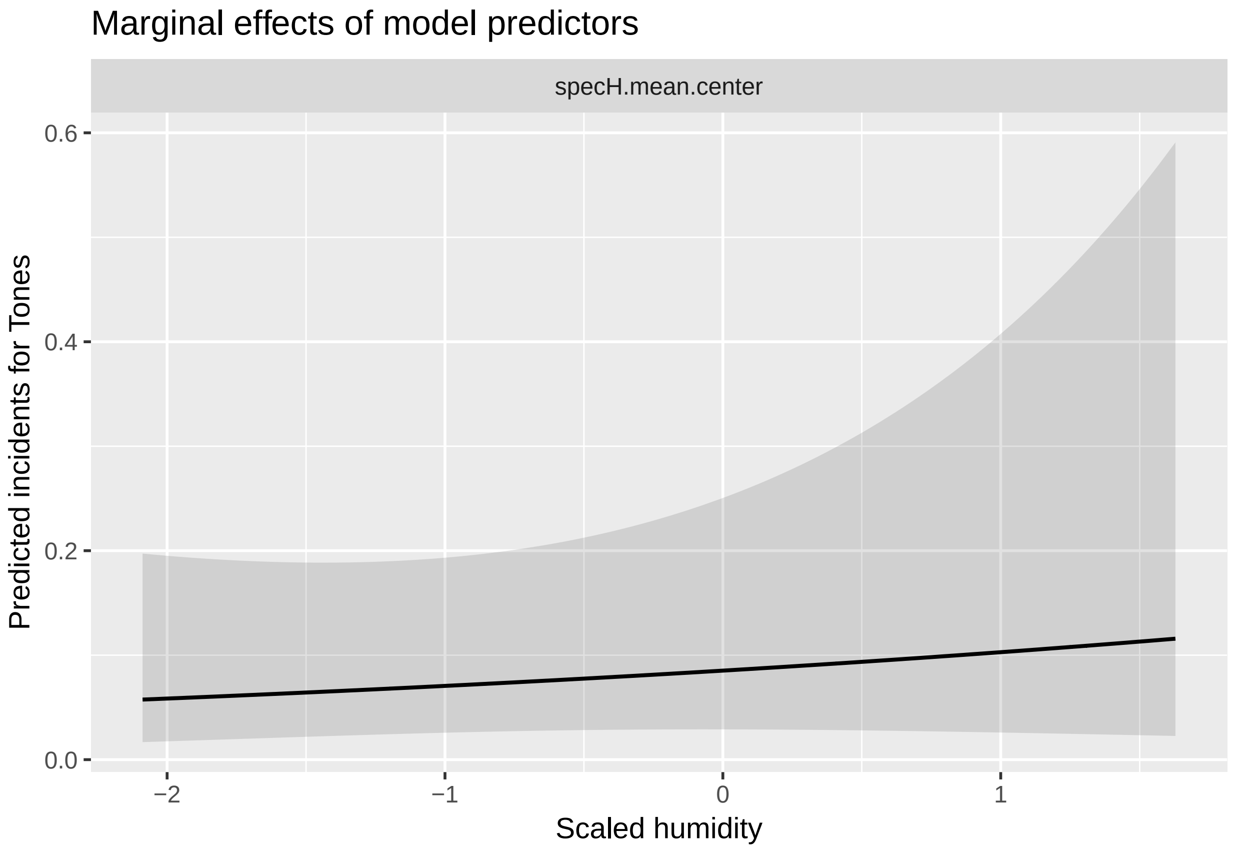

Explore the random effects:

```
x = ranef(m3)
x2 = x$autotyp.area$specH.mean.center
names(x2) = rownames(x$autotyp.area)
dp = dotplot(sort(x2), xlab='Humidity Random Effect')
dp
```

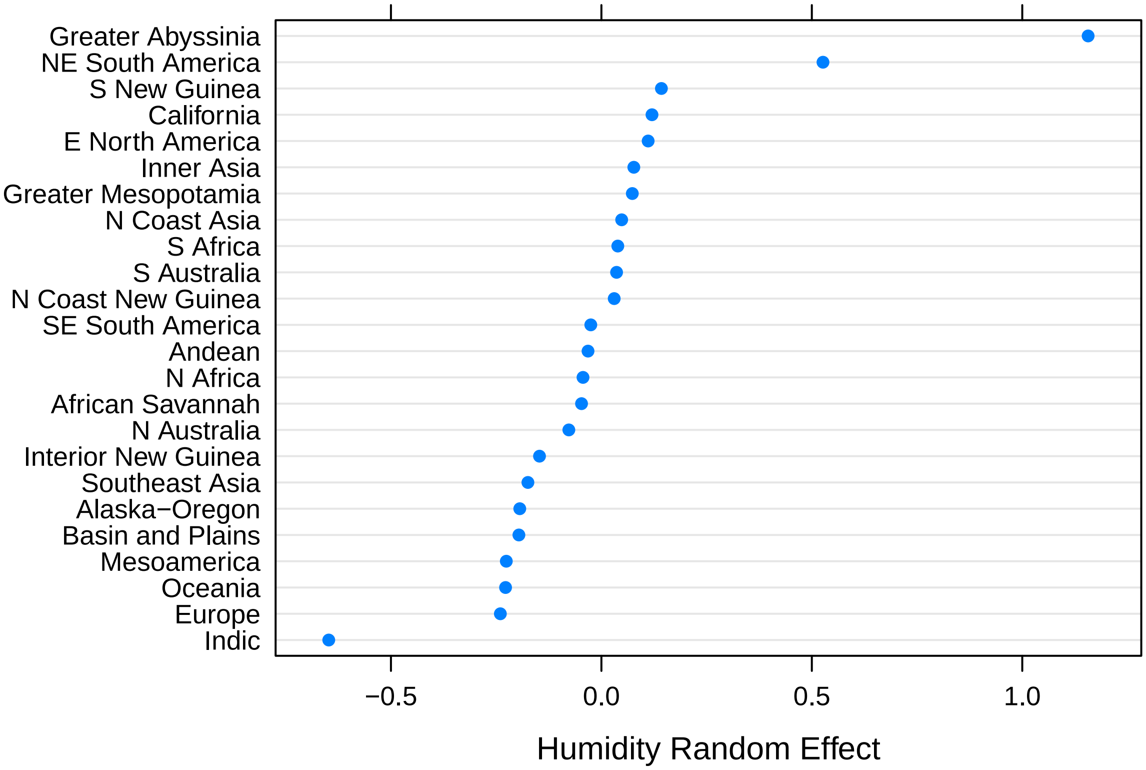


```
pdf("../results/PHOIBLE_ranef.pdf", height=5, width=4)
dp
dev.off()
```

```
## pdf
##   2
```

There was no significant main effect of humidity (beta = 0.19 , log likelihood difference = 0.22 , df = 1 , Chi Squared = 0.45 , p = 0.5).

Contribution of each random effect:

```
m3.noFam.slope = glmer(Tones~1 +specH.mean.center  +
             (1|Family) +
             (1+specH.mean.center|autotyp.area),
           data=p, family=poisson,
           control = glmerControl(optimizer = 'bobyqa'))

m3.noArea.slope = glmer(Tones~1 +specH.mean.center  +
             (1+specH.mean.center||Family) +
             (1|autotyp.area),
           data=p, family=poisson,
           control = glmerControl(optimizer = 'bobyqa'))

m3.noFam.int = glmer(Tones~1 +specH.mean.center  +
             (0+specH.mean.center||Family) +
             (1+specH.mean.center|autotyp.area),
           data=p, family=poisson,
           control = glmerControl(optimizer = 'bobyqa'))

m3.noArea.int = glmer(Tones~1 +specH.mean.center  +
```

```
             (1+specH.mean.center||Family) +
             (0+specH.mean.center|autotyp.area),
           data=p, family=poisson,
           control = glmerControl(optimizer = 'bobyqa'))

res = sapply(list(m3,m3.noFam.int,m3.noFam.slope,m3.noArea.int,m3.noArea.slope),
       function(X){
         summary(X)$coefficients[2,]
       })
res= t(res)
rownames(res) = c("Full model",
                  "No family intercept",
                  "No family slope",
                  "No area intercept",
                  "No area slope")
res
```

```
##                      Estimate Std. Error   z value     Pr(>|z|)
## Full model          0.1882675 0.27602135 0.6820760 0.4951909143
## No family intercept 0.7481830 0.40908827 1.8289036 0.0674140461
## No family slope     0.1882683 0.27603041 0.6820565 0.4952032177
## No area intercept   0.5244216 0.19436207 2.6981686 0.0069722114
## No area slope       0.1626112 0.04745492 3.4266460 0.0006110851
```

```
cat(print(xtable(res, digits = c(0,2,2,2,3)),"latex"),
    "../results/ToneModel_RandomEffectsResults.tex")
```

```
## % latex table generated in R 3.3.1 by xtable 1.8-2 package
## % Wed Oct 25 13:00:12 2017
## \begin{table}[ht]
## \centering
## \begin{tabular}{rrrrr}
##   \hline
##  & Estimate & Std. Error & z value & Pr($>$$|$$z$$|$) \\
##   \hline
## Full model & 0.19 & 0.28 & 0.68 & 0.495 \\
##   No family intercept & 0.75 & 0.41 & 1.83 & 0.067 \\
##   No family slope & 0.19 & 0.28 & 0.68 & 0.495 \\
##   No area intercept & 0.52 & 0.19 & 2.70 & 0.007 \\
##   No area slope & 0.16 & 0.05 & 3.43 & 0.001 \\
##    \hline
## \end{tabular}
## \end{table}
## % latex table generated in R 3.3.1 by xtable 1.8-2 package
## % Wed Oct 25 13:00:12 2017
## \begin{table}[ht]
## \centering
## \begin{tabular}{rrrrr}
##   \hline
##  & Estimate & Std. Error & z value & Pr($>$$|$$z$$|$) \\
##   \hline
## Full model & 0.19 & 0.28 & 0.68 & 0.495 \\
##   No family intercept & 0.75 & 0.41 & 1.83 & 0.067 \\
##   No family slope & 0.19 & 0.28 & 0.68 & 0.495 \\
##   No area intercept & 0.52 & 0.19 & 2.70 & 0.007 \\
```

```
##   No area slope & 0.16 & 0.05 & 3.43 & 0.001 \\
##    \hline
## \end{tabular}
## \end{table}
##  ../results/ToneModel_RandomEffectsResults.tex
```

Non-linear effects

Note that there is a significant non-linear relationship between tone and humidity:

```
m4 = glmer(Tones~1 +specH.mean.center  +
             I(specH.mean.center ^2) +
             (1+specH.mean.center||Family) +
             (1+specH.mean.center|autotyp.area),
           data=p, family=poisson,
           control = glmerControl(optimizer = 'bobyqa'))

anova(m3,m4)
```

```
## Data: p
## Models:
## m3: Tones ~ 1 + specH.mean.center + (1 + specH.mean.center || Family) +
## m3:     (1 + specH.mean.center | autotyp.area)
## m4: Tones ~ 1 + specH.mean.center + I(specH.mean.center^2) + (1 +
## m4:     specH.mean.center || Family) + (1 + specH.mean.center | autotyp.area)
##    Df    AIC    BIC  logLik deviance  Chisq Chi Df Pr(>Chisq)
## m3  7 3090.1 3125.1 -1538.0   3076.1
## m4  8 3079.3 3119.3 -1531.6   3063.3 12.815      1  0.0003438 ***
## ---
## Signif. codes:  0 '***' 0.001 '**' 0.01 '*' 0.05 '.' 0.1 ' ' 1
```

```
summary(m4)
```

```
## Generalized linear mixed model fit by maximum likelihood (Laplace
##   Approximation) [glmerMod]
##  Family: poisson  ( log )
## Formula: Tones ~ 1 + specH.mean.center + I(specH.mean.center^2) + (1 +
##     specH.mean.center || Family) + (1 + specH.mean.center | autotyp.area)
##    Data: p
## Control: glmerControl(optimizer = "bobyqa")
##
##      AIC      BIC   logLik deviance df.resid
##   3079.3   3119.3  -1531.6   3063.3     1086
##
## Scaled residuals:
##     Min      1Q  Median      3Q     Max
## -1.9413 -0.4931 -0.1791  0.2320 10.1889
##
## Random effects:
##  Groups       Name              Variance Std.Dev. Corr
##  Family       (Intercept)       3.3016   1.8170
##  Family.1     specH.mean.center 0.0000   0.0000
##  autotyp.area (Intercept)       2.6362   1.6236
##               specH.mean.center 0.4373   0.6613   0.40
## Number of obs: 1094, groups:  Family, 119; autotyp.area, 24
```

```
##
## Fixed effects:
##                        Estimate Std. Error z value Pr(>|z|)
## (Intercept)            -2.23844    0.56604  -3.955 7.67e-05 ***
## specH.mean.center       0.07466    0.30540   0.244 0.806877
## I(specH.mean.center^2) -0.21608    0.06147  -3.515 0.000439 ***
## ---
## Signif. codes:  0 '***' 0.001 '**' 0.01 '*' 0.05 '.' 0.1 ' ' 1
##
## Correlation of Fixed Effects:
##             (Intr) spcH..
## spcH.mn.cnt  0.404
## I(spcH..^2) -0.092  0.104
```

```r
x = sjp.glmer(m4, 'eff',
              vars=c("specH.mean.center"),
              show.scatter = T, show.ci = T,
              prnt.plot = F)
x[[1]]+ xlab("Scaled humidity")
```

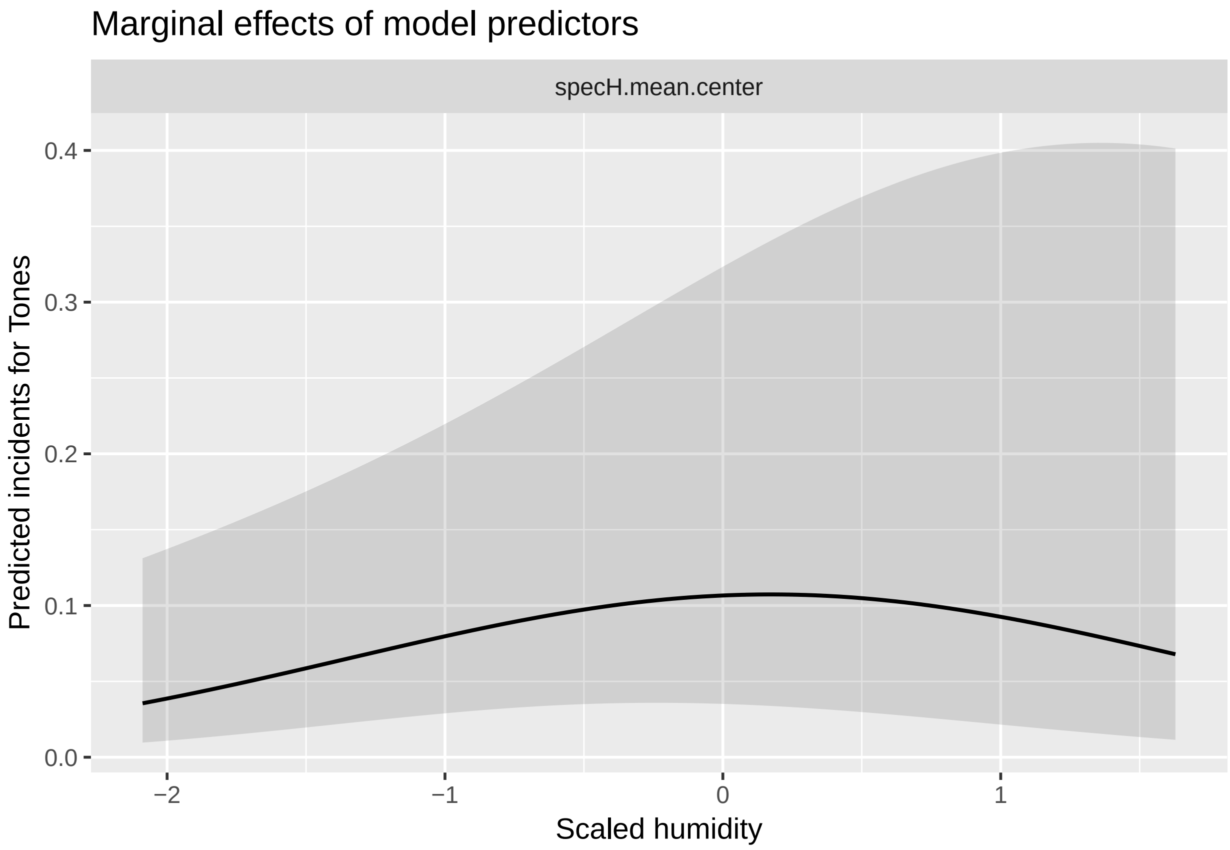

Mixed effects modelling using MCMCglmm

We run the same model as model `m3` above, but this time in the package MCMCglmm, which converges on estimates using a Bayesian Monte Carlo Markov chain.

First we set up the model priors:

```
familyRandomEffectsN = 2
areaRandomEffectsN = 2

prior.m3 <- list(
  R=list(V=1, n=1, fix=1),
  G=list(G1=list(V        = diag(familyRandomEffectsN),  # family intercept+slope
                 n        = familyRandomEffectsN,
                 alpha.mu = rep(0, familyRandomEffectsN),
                 alpha.V  = diag(familyRandomEffectsN)*25^2),
         G2=list(V        = diag(areaRandomEffectsN), # area intercept+slope
                 n        = areaRandomEffectsN,
                 alpha.mu = rep(0, areaRandomEffectsN),
                 alpha.V  = diag(areaRandomEffectsN)*25^2)))
```

Now we run the model. We run the process for 100,000 iterations, plus a 10,000 iteration burn-in. The sampling of posterior values is thinned to produce 10,000 observations.

```
set.seed(123)
m3.mcmcglmm <- MCMCglmm(
  Tones ~
    specH.mean.center,
  ~ us(1 + specH.mean.center):Family +
    us(1 + specH.mean.center):autotyp.area,
  data   = p,
  family = "gaussian",
  prior  = prior.m3,
  thin   =     10,
  burnin =   1000,
  nitt   = 101000,
  verbose = FALSE)
```

And save the results:

```
save(m3.mcmcglmm, file="../results/m3_mcmcglmm.RDat")
#load("../results/m3_mcmcglmm.RDat")
```

Plots of the model convergence (rendered as png to save space):

```
png("../results/MCMCConvergence1.png")
plot(m3.mcmcglmm$VCV[,1:3])
dev.off()
```

```
## pdf
##   2
```

```
png("../results/MCMCConvergence2.png")
plot(m3.mcmcglmm$VCV[,4:6])
dev.off()
```

```
## pdf
##   2
```

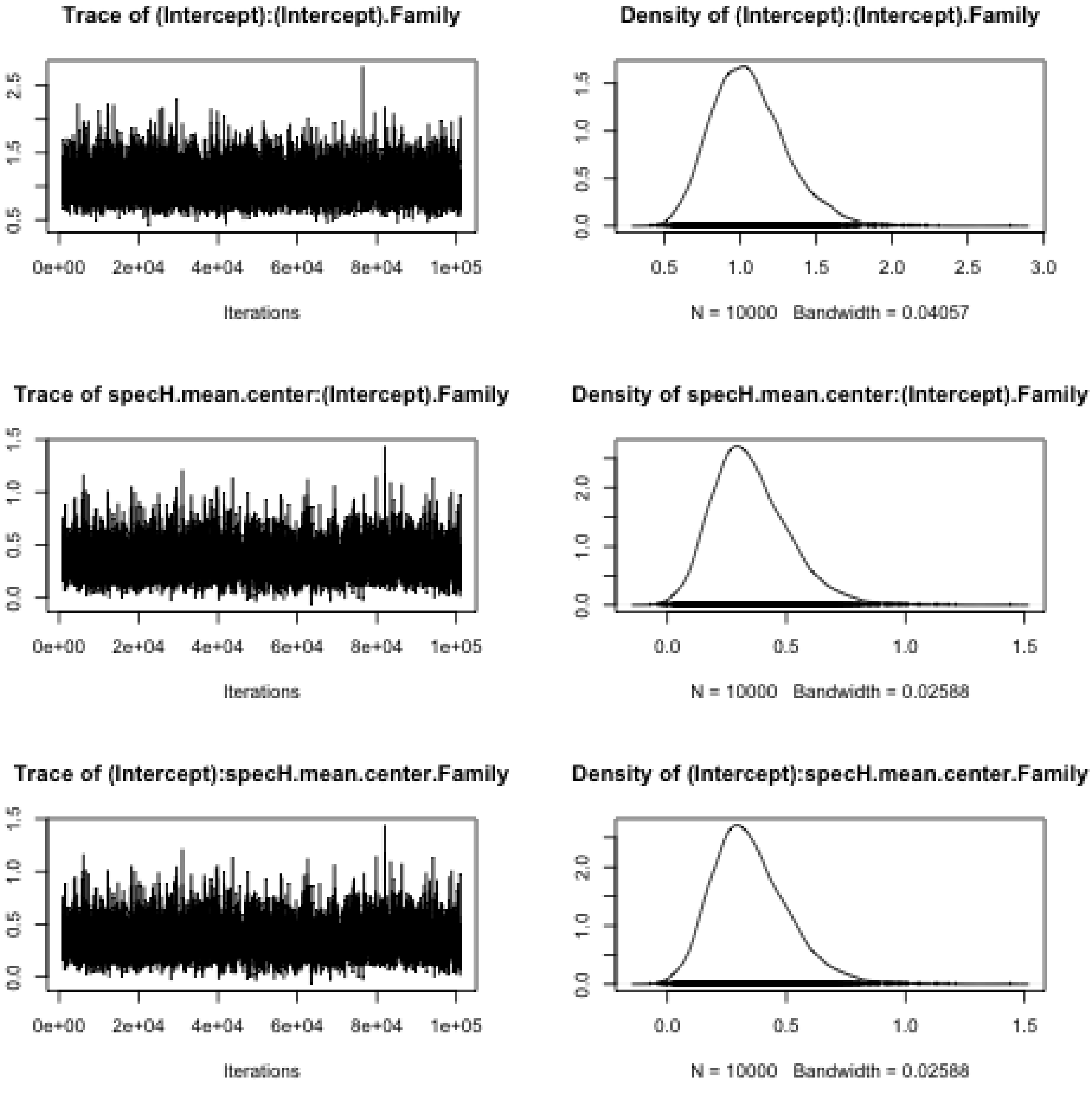
Trace of (Intercept):(Intercept).Family
Iterations
Density of (Intercept):(Intercept).Family
N = 10000 Bandwidth = 0.04057
Trace of specH.mean.center:(Intercept).Family
Iterations
Density of specH.mean.center:(Intercept).Family
N = 10000 Bandwidth = 0.02588
Trace of (Intercept):specH.mean.center.Family
Iterations
Density of (Intercept):specH.mean.center.Family
N = 10000 Bandwidth = 0.02588

Figure 1:

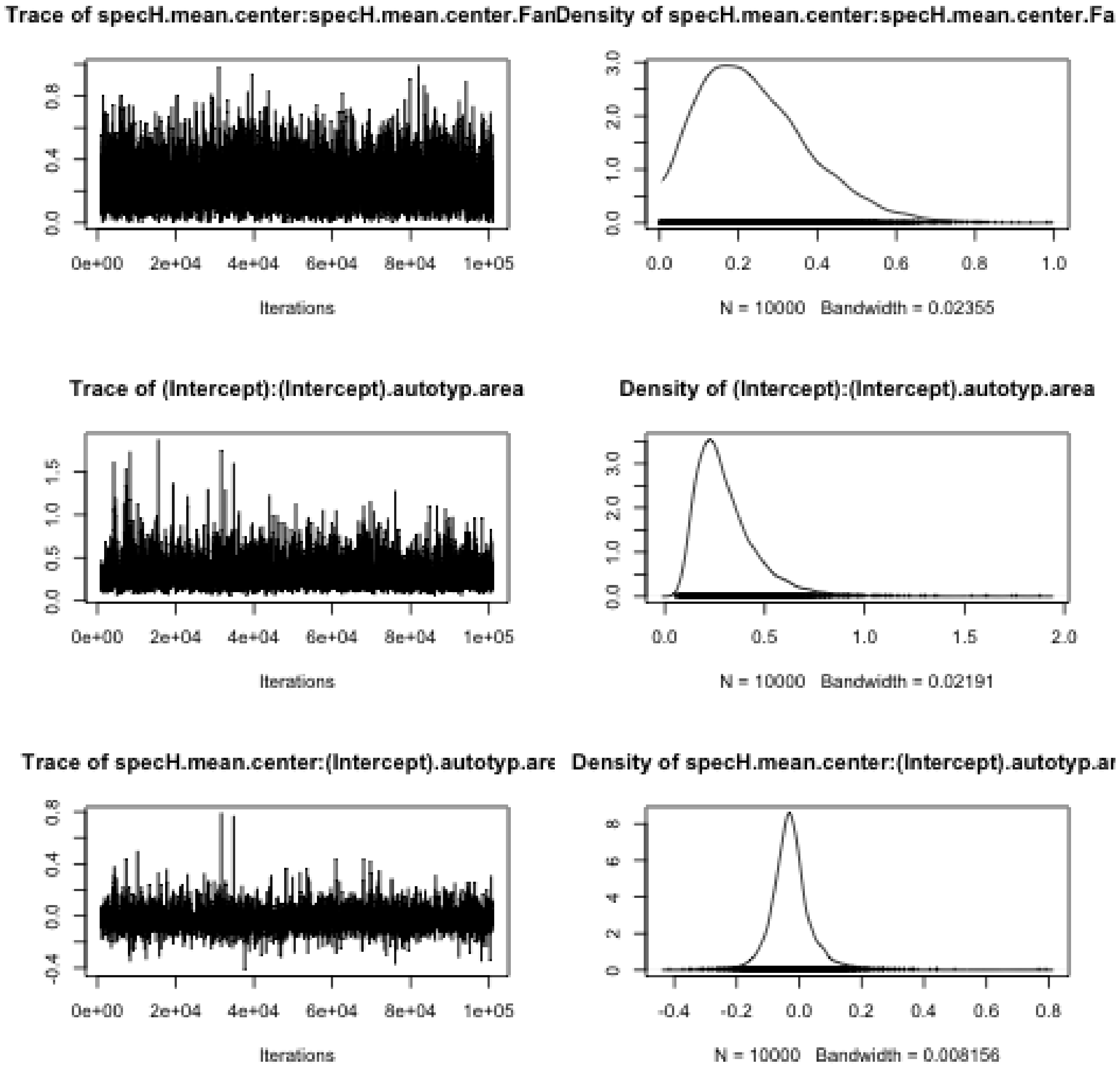

Trace of specH.mean.center:specH.mean.center.Fan
Density of specH.mean.center:specH.mean.center.Fa
Iterations
N = 10000 Bandwidth = 0.02355
Trace of (Intercept):(Intercept).autotyp.area
Density of (Intercept):(Intercept).autotyp.area
Iterations
N = 10000 Bandwidth = 0.02191
Trace of specH.mean.center:(Intercept).autotyp.are
Density of specH.mean.center:(Intercept).autotyp.ar
Iterations
N = 10000 Bandwidth = 0.008156

Figure 2:

Look at the results of the model and fixed effect:

```
sx = summary(m3.mcmcglmm)
sx
```

```
##
##  Iterations = 1001:100991
##  Thinning interval  = 10
##  Sample size  = 10000
##
##  DIC: 4428.272
##
##  G-structure:  ~us(1 + specH.mean.center):Family
##
##                                              post.mean l-95% CI u-95% CI
## (Intercept):(Intercept).Family                  1.0576  0.61742   1.5643
## specH.mean.center:(Intercept).Family            0.3510  0.05611   0.6719
## (Intercept):specH.mean.center.Family            0.3510  0.05611   0.6719
## specH.mean.center:specH.mean.center.Family     0.2419  0.01445   0.5178
##                                              eff.samp
## (Intercept):(Intercept).Family                  9386
## specH.mean.center:(Intercept).Family            3798
## (Intercept):specH.mean.center.Family            3798
## specH.mean.center:specH.mean.center.Family      3733
##
##                ~us(1 + specH.mean.center):autotyp.area
##
##                                                    post.mean   l-95% CI
## (Intercept):(Intercept).autotyp.area                 0.30426  7.921e-02
## specH.mean.center:(Intercept).autotyp.area          -0.02625 -1.655e-01
## (Intercept):specH.mean.center.autotyp.area          -0.02625 -1.655e-01
## specH.mean.center:specH.mean.center.autotyp.area   0.09744  1.046e-07
##                                                    u-95% CI eff.samp
## (Intercept):(Intercept).autotyp.area                 0.6076     1787
## specH.mean.center:(Intercept).autotyp.area          0.1098     4247
## (Intercept):specH.mean.center.autotyp.area          0.1098     4247
## specH.mean.center:specH.mean.center.autotyp.area   0.2460     4148
##
##  R-structure:  ~units
##
##       post.mean l-95% CI u-95% CI eff.samp
## units         1        1        1        0
##
##  Location effects: Tones ~ specH.mean.center
##
##                   post.mean l-95% CI u-95% CI eff.samp  pMCMC
## (Intercept)         0.84040  0.47006  1.20190    10000 <1e-04 ***
## specH.mean.center   0.19783 -0.04012  0.44087     9582  0.106
## ---
## Signif. codes:  0 '***' 0.001 '**' 0.01 '*' 0.05 '.' 0.1 ' ' 1
```

```
fe = m3.mcmcglmm$Sol[,2]
dx = density(fe)
plot(dx, main='', xlab='Parameter estimate')
abline(v=0)
```

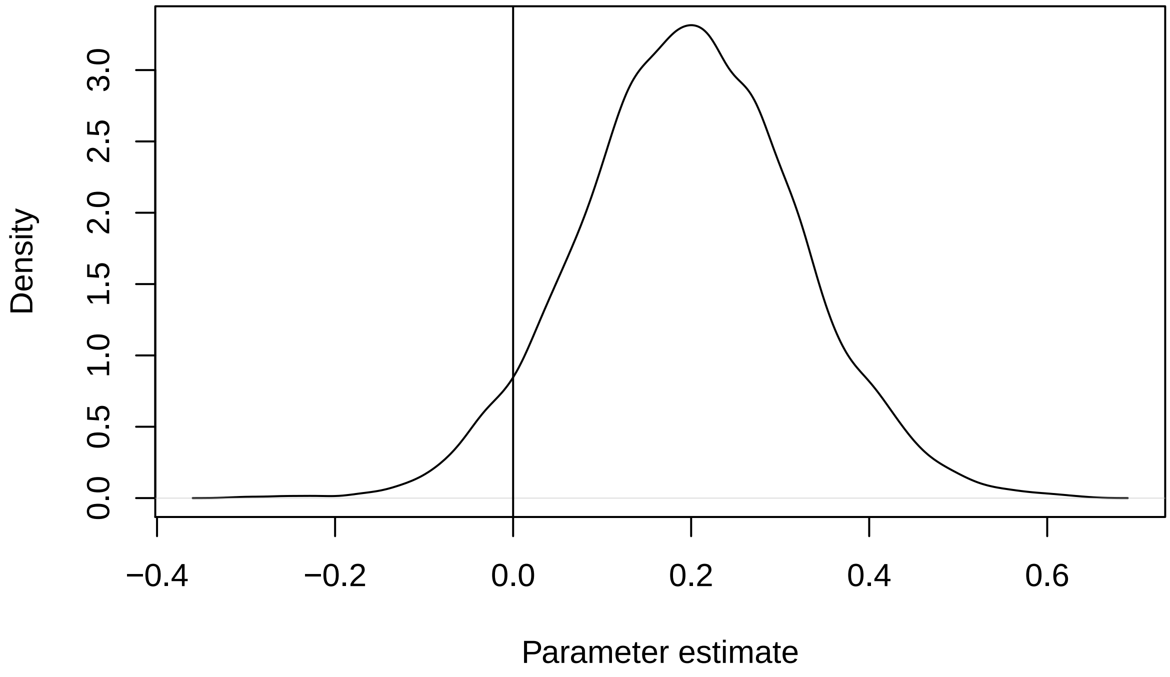

Plot the range of estimates for the random effects:

```
re = m3.mcmcglmm$VCV
re = as.data.frame(re)
re.area = sample(re$`specH.mean.center:specH.mean.center.autotyp.area`,10000)
re.area.d = density(re.area)
plot(re.area.d)
```

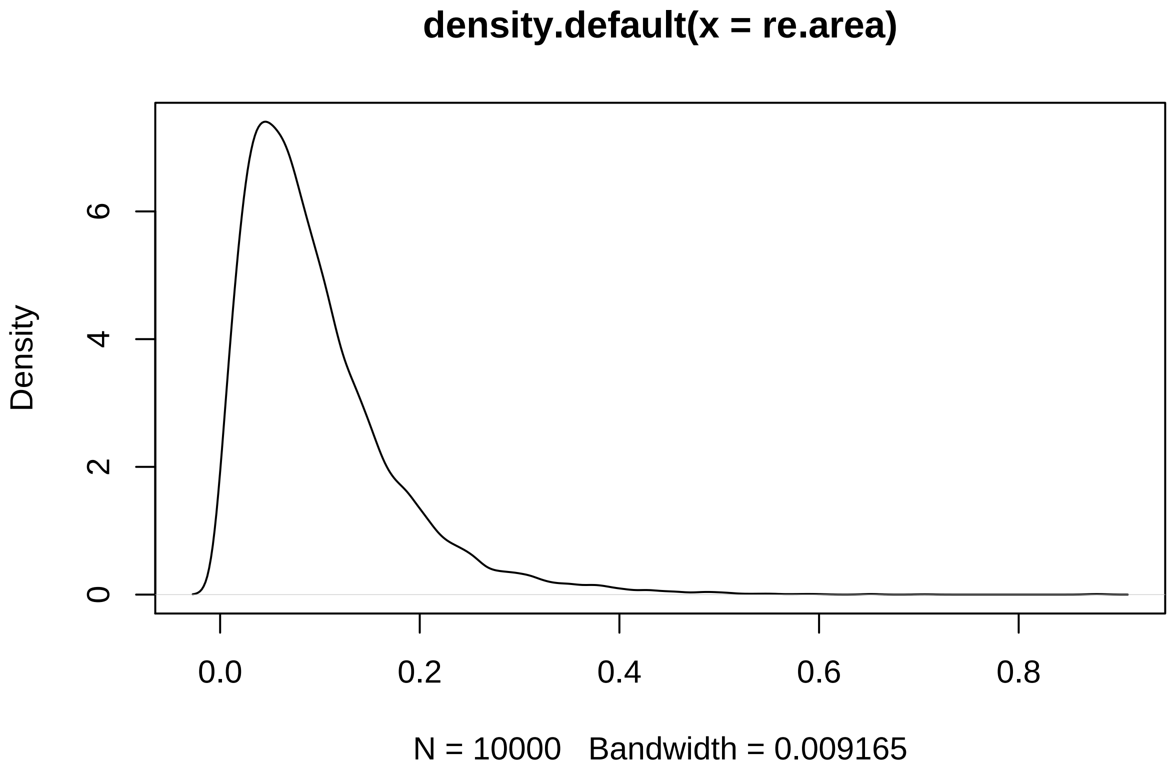

Supplement: Supplementary file 1 [file DataSheet1.PDF]
