# Supplementary material for: Robust, Causal, and Incremental Approaches to Investigating Linguistic Adaptation
Source: Front Psychol. 2018 Feb 21;9:166. doi: 10.3389/fpsyg.2018.00166 (PMC5826341; doi:10.3389/fpsyg.2018.00166)

Comparing ANU and PHOIBLE estimates for tone

Load libraries

```
library(lme4)
library(psych)
library(ggplot2)
library(sjPlot)
```

Load data

```
combined = read.csv("../data/phoibleAndANU_combined.csv", stringsAsFactors = F)

gx = ggplot(combined, aes(x = Tones, y=ANU.Tones)) +
  geom_count() + #scale_size_area(breaks=c(0,5,10,50,100,200,500)) +
  scale_y_continuous(breaks=c(0,2,4,6,8,10,12)) +
  scale_x_continuous(breaks=c(0,2,4,6,8,10)) +
  stat_smooth(method='lm') +
  xlab("Number of tones (PHOIBLE)") +
  ylab("Number of tones (ANU)")

pdf('../results/ANU_vs_PHOIBLE.pdf', width = 4.5, height = 4)
gx
dev.off()
```

```
## pdf
##   2
```

Test agreement

Look at correlation and weighted kappa:

```
cor.test(combined$Tones, combined$ANU.Tones)
```

```
##
##  Pearson's product-moment correlation
##
## data:  combined$Tones and combined$ANU.Tones
## t = 20.346, df = 665, p-value < 2.2e-16
## alternative hypothesis: true correlation is not equal to 0
## 95 percent confidence interval:
##  0.5703099 0.6640953
## sample estimates:
##       cor
## 0.6194076
```

```
cohen.kappa(cbind(combined$Tones, combined$ANU.Tones))
```

```
## Call: cohen.kappa1(x = x, w = w, n.obs = n.obs, alpha = alpha, levels = levels)
##
## Cohen Kappa and Weighted Kappa correlation coefficients and confidence boundaries
##                  lower estimate upper
## unweighted kappa  0.34     0.38  0.43
## weighted kappa    0.55     0.61  0.67
##
##  Number of subjects = 667
```

Test agreement on tone vs non-tone:

```
tx = table(combined$ANU.Tones, combined$Tones)
write.csv(tx,"../results/ANU_vs_PHOIBLE.csv")

tx2 = table(as.numeric(combined$ANU.Tones>0),combined$Tones>0)

sum(diag(tx2)/sum(tx2))
```

```
## [1] 0.8230885
```

```
cohen.kappa(x=tx2)
```

```
## Call: cohen.kappa1(x = x, w = w, n.obs = n.obs, alpha = alpha, levels = levels)
##
## Cohen Kappa and Weighted Kappa correlation coefficients and confidence boundaries
##                  lower estimate upper
## unweighted kappa  0.58     0.64   0.7
## weighted kappa    0.58     0.64   0.7
##
##  Number of subjects = 667
```

```
tx3 = table(as.numeric(combined$ANU.Tones>=3),combined$Tones>=3)

sum(diag(tx3)/sum(tx3))
```

```
## [1] 0.7976012
```

```
cohen.kappa(x=tx3)
```

```
## Call: cohen.kappa1(x = x, w = w, n.obs = n.obs, alpha = alpha, levels = levels)
##
## Cohen Kappa and Weighted Kappa correlation coefficients and confidence boundaries
##                  lower estimate upper
## unweighted kappa  0.42     0.49  0.56
## weighted kappa    0.42     0.49  0.56
##
##  Number of subjects = 667
```

Are the differences biased?

Calculate the difference and plot

```
combined$Tones.diff = combined$Tones -combined$ANU.Tones
combined$Tones.diff.center = scale(combined$Tones.diff)

plot(combined$Tones.diff, combined$specH.mean)
```

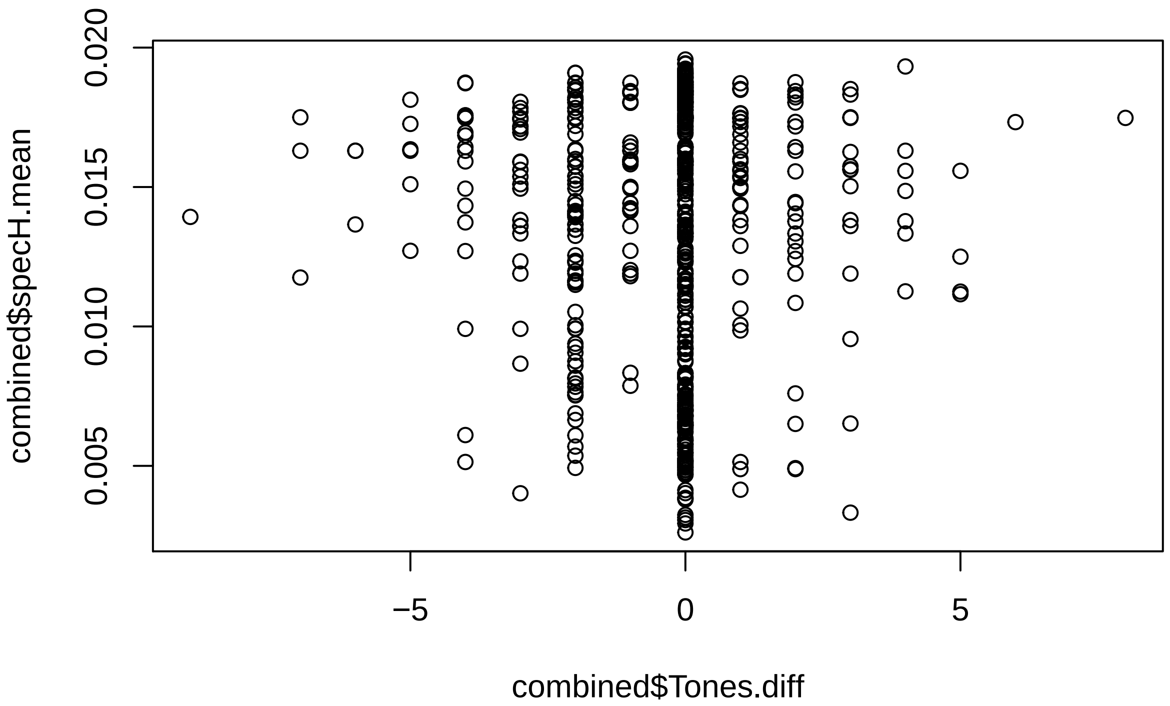


```
cor.test(combined$Tones.diff, combined$specH.mean)
```

```
##
##  Pearson's product-moment correlation
##
## data:  combined$Tones.diff and combined$specH.mean
## t = -1.0695, df = 665, p-value = 0.2852
## alternative hypothesis: true correlation is not equal to 0
## 95 percent confidence interval:
##  -0.11698400  0.03458683
## sample estimates:
##         cor
## -0.04143698
```

Test for biases in langauge families, areas and by humidity:

```
m3 = lmer(Tones.diff.center ~ 1 + (1|Family) +  (1|autotyp.area),
           data = combined)

m.familyInt = lmer(Tones.diff.center ~ 1 + (1|autotyp.area),
           data = combined)

m.areaInt = lmer(Tones.diff.center ~ 1 + (1|Family),
           data = combined)

combined$specH.mean.center = scale(combined$specH.mean)

m.specHMean = lmer(Tones.diff.center ~ 1 + specH.mean.center +
                     (1|Family) +  (1|autotyp.area),
                   data = combined)
```

Contribution of intercept for family:

```r
anova(m3,m.familyInt)
```

```
## refitting model(s) with ML (instead of REML)

## Data: combined
## Models:
## m.familyInt: Tones.diff.center ~ 1 + (1 | autotyp.area)
## m3: Tones.diff.center ~ 1 + (1 | Family) + (1 | autotyp.area)
##             Df    AIC    BIC  logLik deviance  Chisq Chi Df Pr(>Chisq)
## m.familyInt  3 1897.9 1911.4 -945.93   1891.9
## m3           4 1897.0 1915.0 -944.52   1889.0 2.8331      1    0.09234 .
## ---
## Signif. codes:  0 '***' 0.001 '**' 0.01 '*' 0.05 '.' 0.1 ' ' 1
```

Contribution of intercept for area:

```r
anova(m3,m.areaInt)
```

```
## refitting model(s) with ML (instead of REML)

## Data: combined
## Models:
## m.areaInt: Tones.diff.center ~ 1 + (1 | Family)
## m3: Tones.diff.center ~ 1 + (1 | Family) + (1 | autotyp.area)
##           Df  AIC    BIC  logLik deviance Chisq Chi Df Pr(>Chisq)
## m.areaInt  3 1895 1908.5 -944.52     1889
## m3         4 1897 1915.0 -944.52     1889     0      1          1
```

Contribution of humidity:

```r
anova(m3,m.specHMean)
```

```
## refitting model(s) with ML (instead of REML)

## Data: combined
## Models:
## m3: Tones.diff.center ~ 1 + (1 | Family) + (1 | autotyp.area)
## m.specHMean: Tones.diff.center ~ 1 + specH.mean.center + (1 | Family) + (1 |
## m.specHMean:     autotyp.area)
##             Df    AIC    BIC  logLik deviance  Chisq Chi Df Pr(>Chisq)
## m3           4 1897.0 1915.0 -944.52   1889.0
## m.specHMean  5 1898.4 1920.9 -944.20   1888.4 0.6321      1     0.4266
```

Plot random effects:

```r
sjp.glmer(m3, 're', sort.est = "(Intercept)")
```

```
## Plotting random effects...
## Plotting random effects...
```

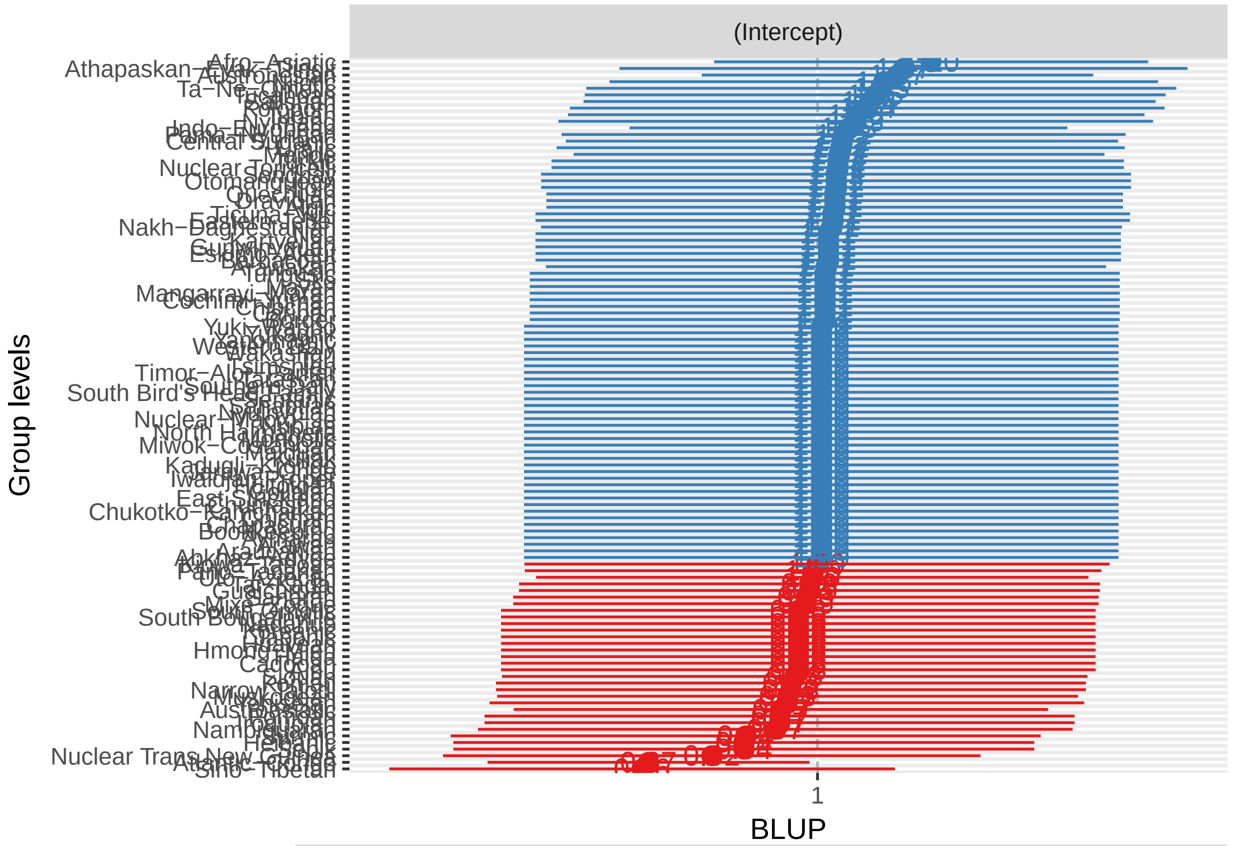
(Intercept)
Group levels
BLUP
1

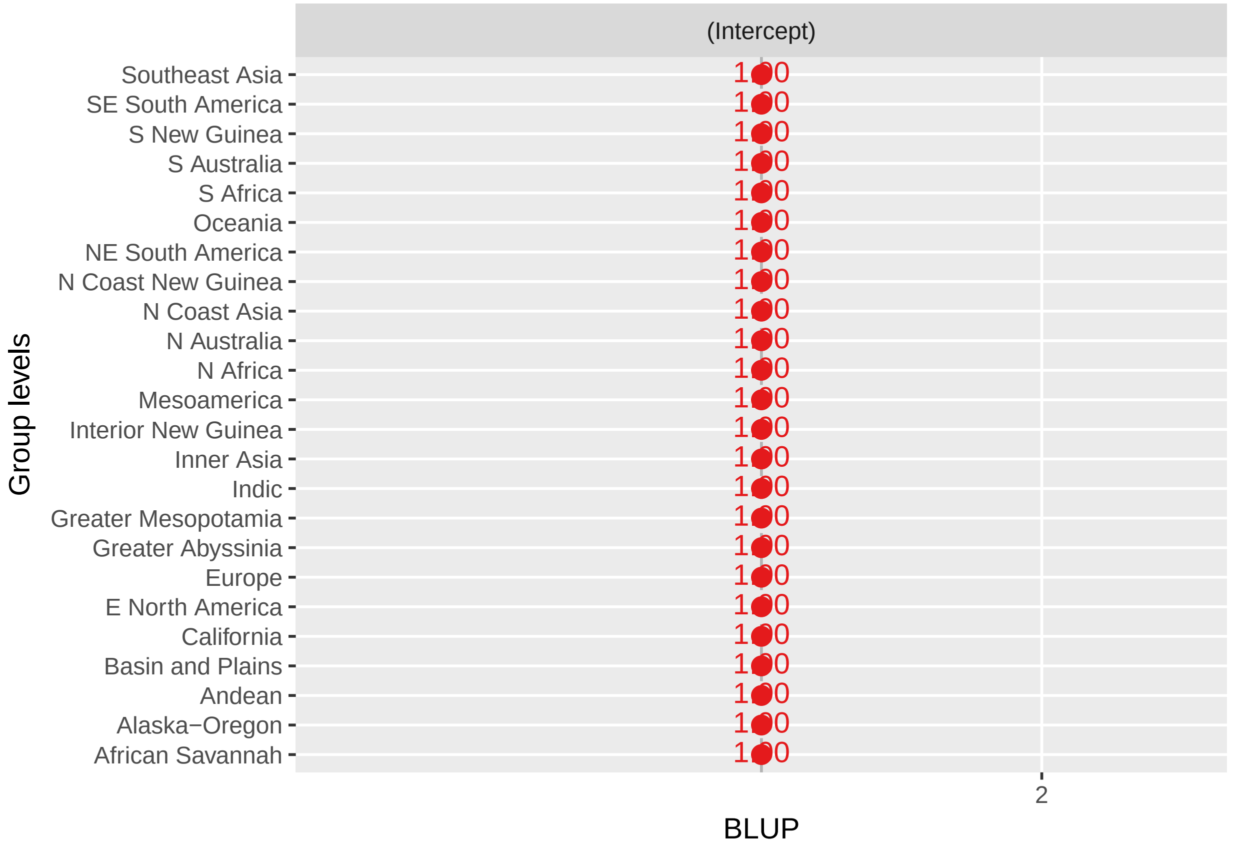
(Intercept)
Southeast Asia
SE South America
S New Guinea
S Australia
S Africa
Oceania
NE South America
N Coast New Guinea
N Coast Asia
N Australia
N Africa
Mesoamerica
Interior New Guinea
Inner Asia
Indic
Greater Mesopotamia
Greater Abyssinia
Europe
E North America
California
Basin and Plains
Andean
Alaska−Oregon
African Savannah
Group levels
BLUP
2

```
plot(combined$Tones.diff.center,predict(m3))
```

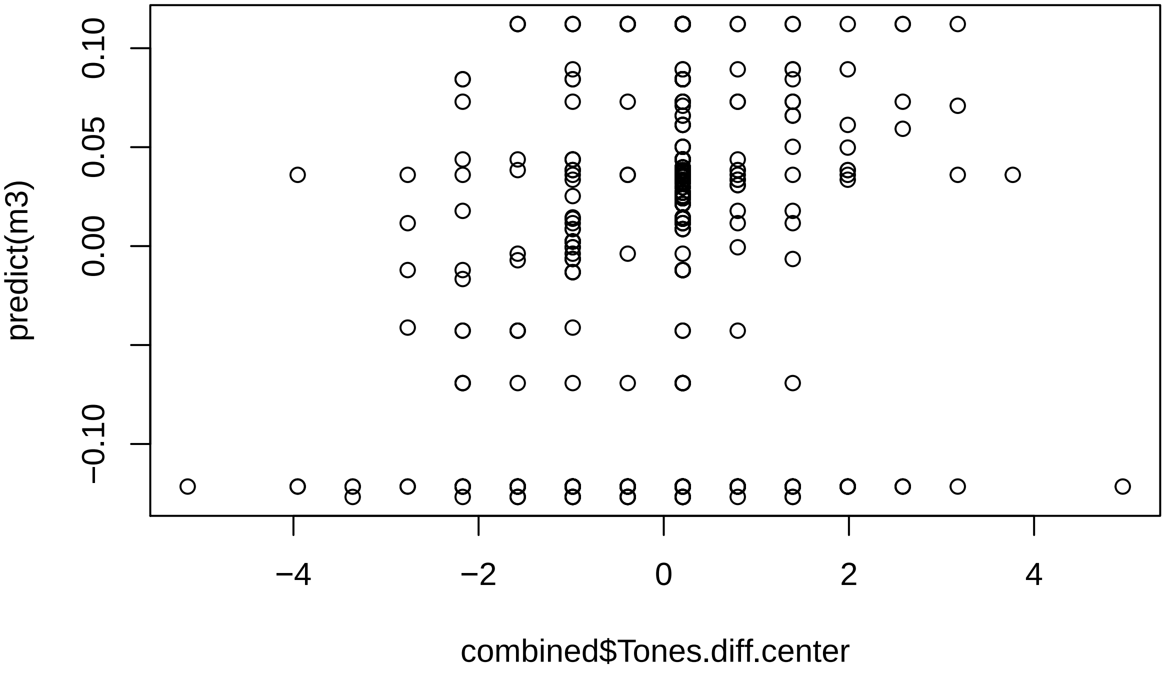

Supplement: Supplementary file 2 [file DataSheet2.PDF]
