# Supplementary material for: Robust, Causal, and Incremental Approaches to Investigating Linguistic Adaptation
Source: Front Psychol. 2018 Feb 21;9:166. doi: 10.3389/fpsyg.2018.00166 (PMC5826341; doi:10.3389/fpsyg.2018.00166)

Vowels and humidity: PHOIBLE replication

```
library(lme4)
library(sjPlot)
library(ggplot2)
library(caret)
library(car)
library(MCMCglmm)
library(reshape2)
```

Load data

The PHOIBLE database contains data for 1667 varieites with unique glottolog codes. There are multiple sources for some languages. PHOIBLE suggests a 'trump' source for each of these cases, which we select if available, otherwise we seleted the source with the highest number of phonemes listed.

```
p = read.csv("../data/phoibleVowelsAndHumidity.csv")
p = p[complete.cases(p[,
                       c("Family",'autotyp.area',
                         'specH.mean')]),]
```

There are now 1730 datapoints.

Transform, scale and center the data. The proportion of vowels to consonants is ratio in theory, but in practice the values are constrained below 1. In any case, the mdoel estimates differ very little using a log transformation or a simple scaling.

```
pp = preProcess(p[,c('Tones','specH.mean')], method="BoxCox")

h.lambda = pp$bc$specH.mean$lambda

p$specH.mean.center = bcPower(p$specH.mean, lambda = h.lambda)

p$specH.mean.center = scale(p$specH.mean.center)

hist(p$specH.mean.center)
```

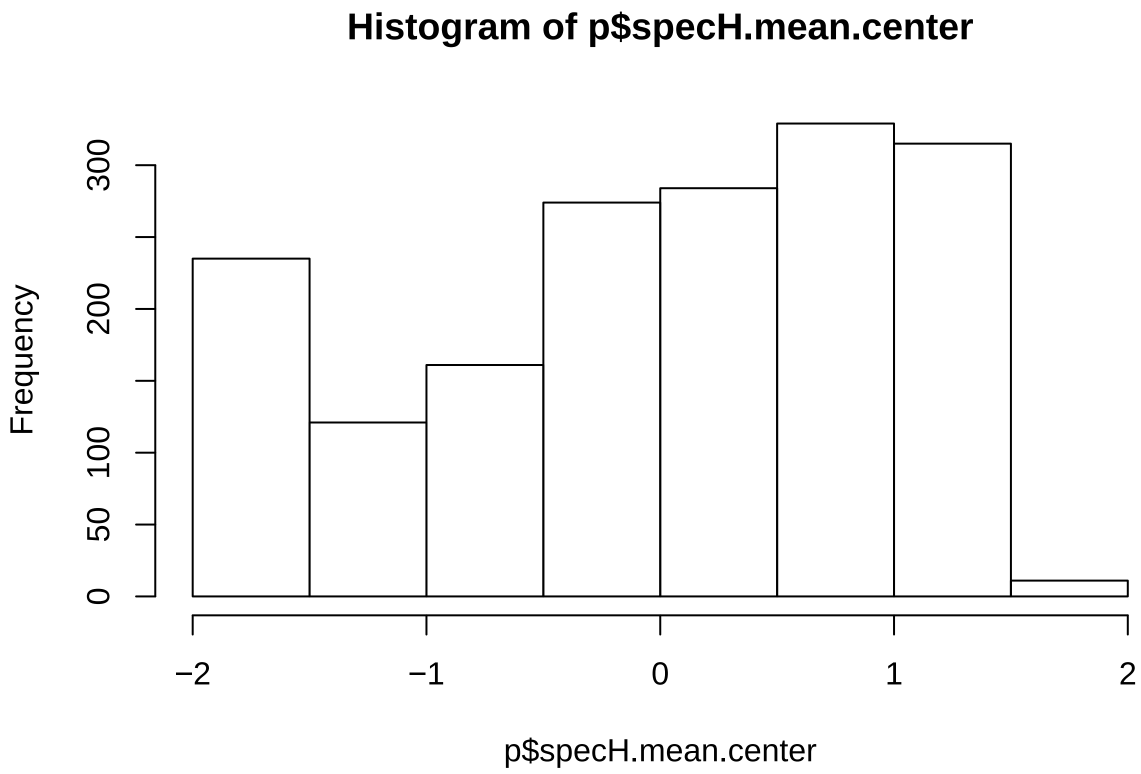


```r
p$prop.vowels = p$Vowels/(p$Consonants + p$Vowels)
p$prop.vowels.scaled = scale(p$prop.vowels)

p$inventorySize = p$Consonants + p$Vowels
p$inventorySize = scale(p$inventorySize)

hist(p$prop.vowels.scaled)
```

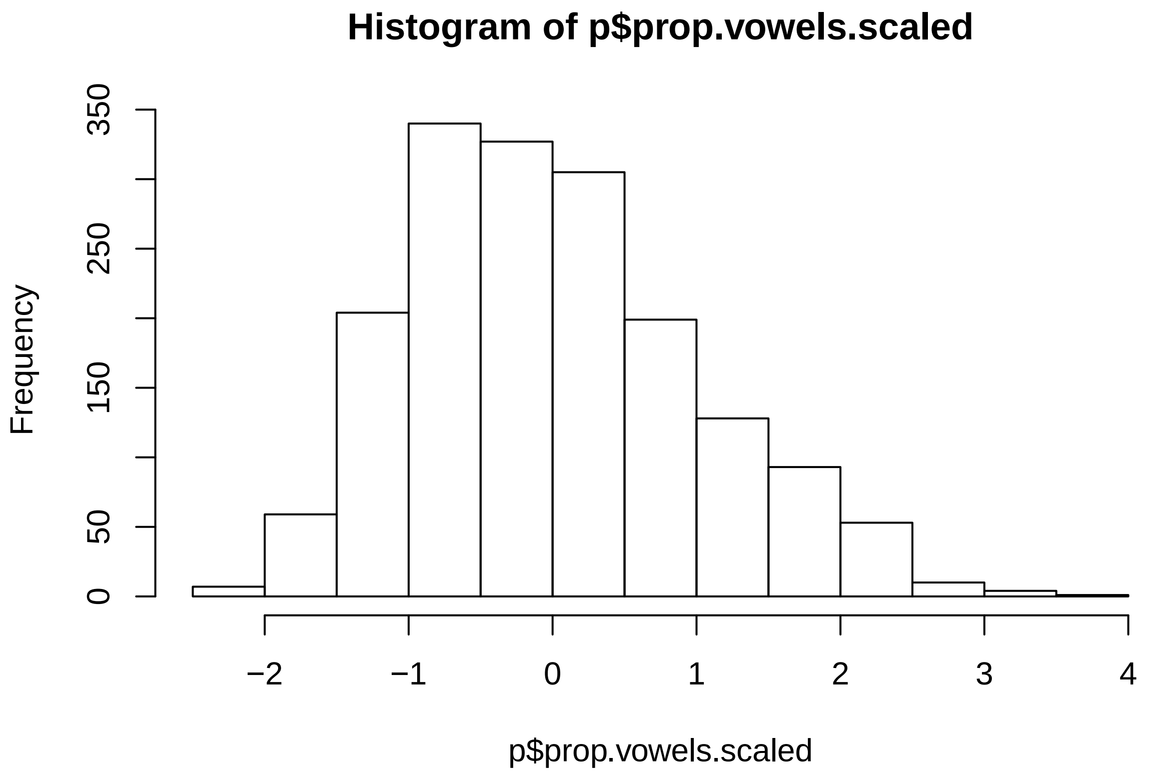

Plots

Plot the raw data:

```
gx = ggplot(p, aes(y=prop.vowels, x=specH.mean)) +
  geom_point() +stat_smooth() +
  ylab("Proportion of vowels") +
  xlab("Specific Humidity")
gx
```

```
## `geom_smooth()` using method = 'gam' and formula 'y ~ s(x, bs = "cs")'
```

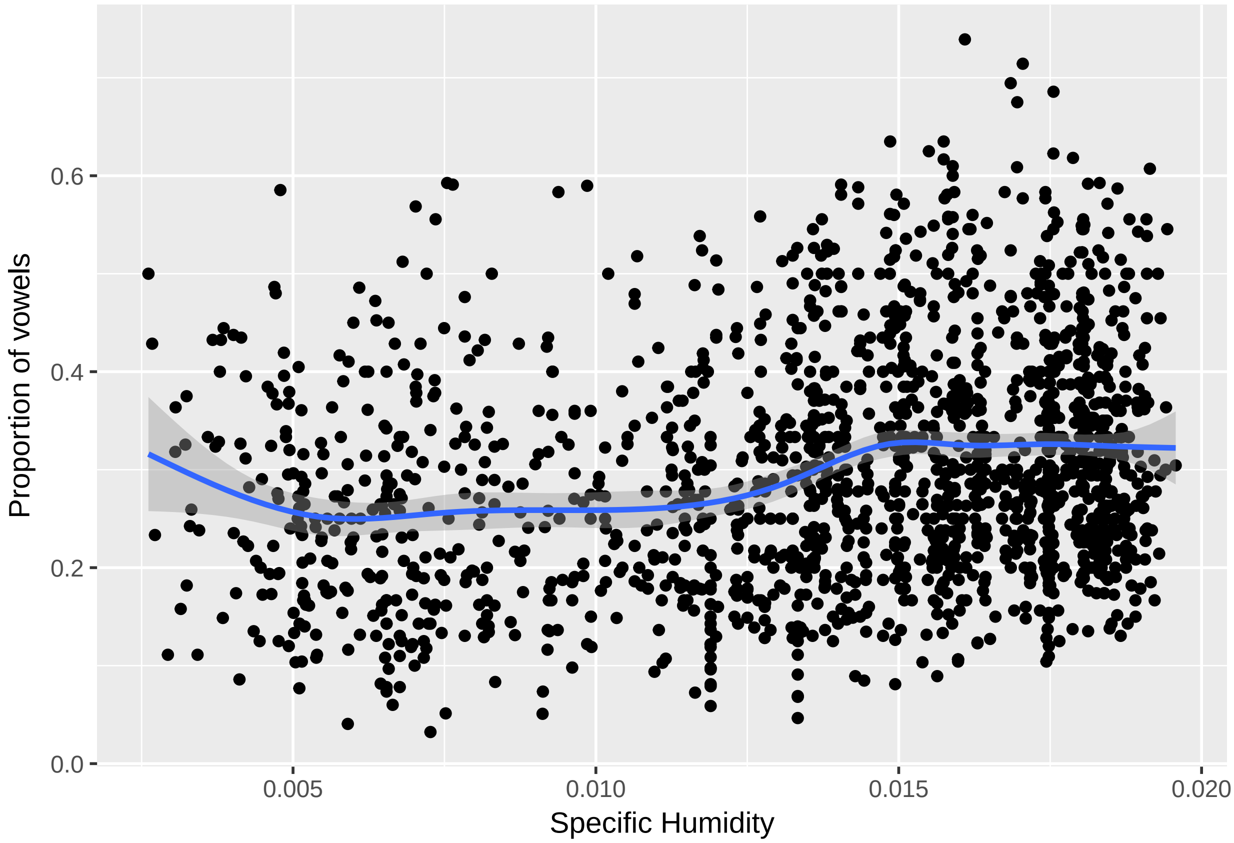


```
# Write to file
pdf("../results/PropVowels_SpecH.pdf", width=4, height=4)
gx
```

```
## `geom_smooth()` using method = 'gam' and formula 'y ~ s(x, bs = "cs")'
```

```
dev.off()
```

```
## pdf
##   2
```

Check for correlation between proportion of vowels and total inventory size:

```
plot(p$prop.vowels, p$inventorySize)
```

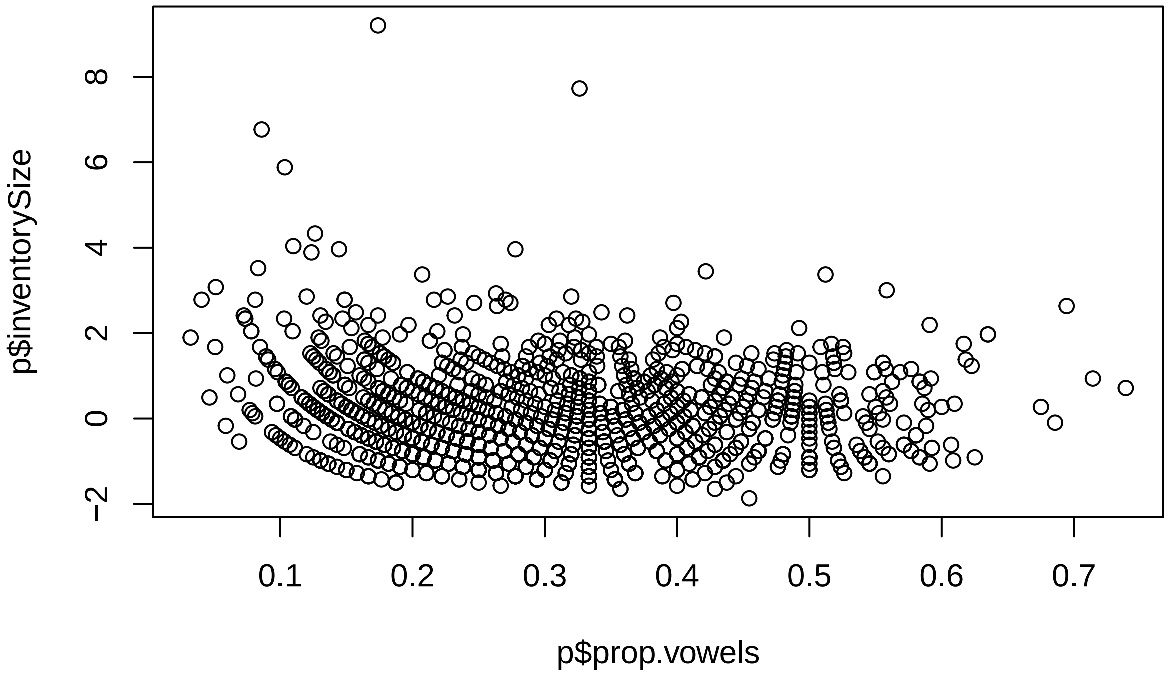


```r
cor.test(p$prop.vowels, p$inventorySize)
```

```
##
##  Pearson's product-moment correlation
##
## data:  p$prop.vowels and p$inventorySize
## t = -2.6058, df = 1728, p-value = 0.009243
## alternative hypothesis: true correlation is not equal to 0
## 95 percent confidence interval:
##  -0.10936982 -0.01548172
## sample estimates:
##         cor
## -0.06256419
```

```r
p$inventorySize.cat = cut(p$inventorySize, quantile(p$inventorySize,seq(0,1,length.out = 3)), include.lo

gx = ggplot(p, aes(y=prop.vowels, x=specH.mean,
                  colour=inventorySize.cat)) +
  geom_point() +stat_smooth() +
  ylab("Proportion of vowels") +
  xlab("Specific Humidity")
gx
```

```
## `geom_smooth()` using method = 'loess' and formula 'y ~ x'
```

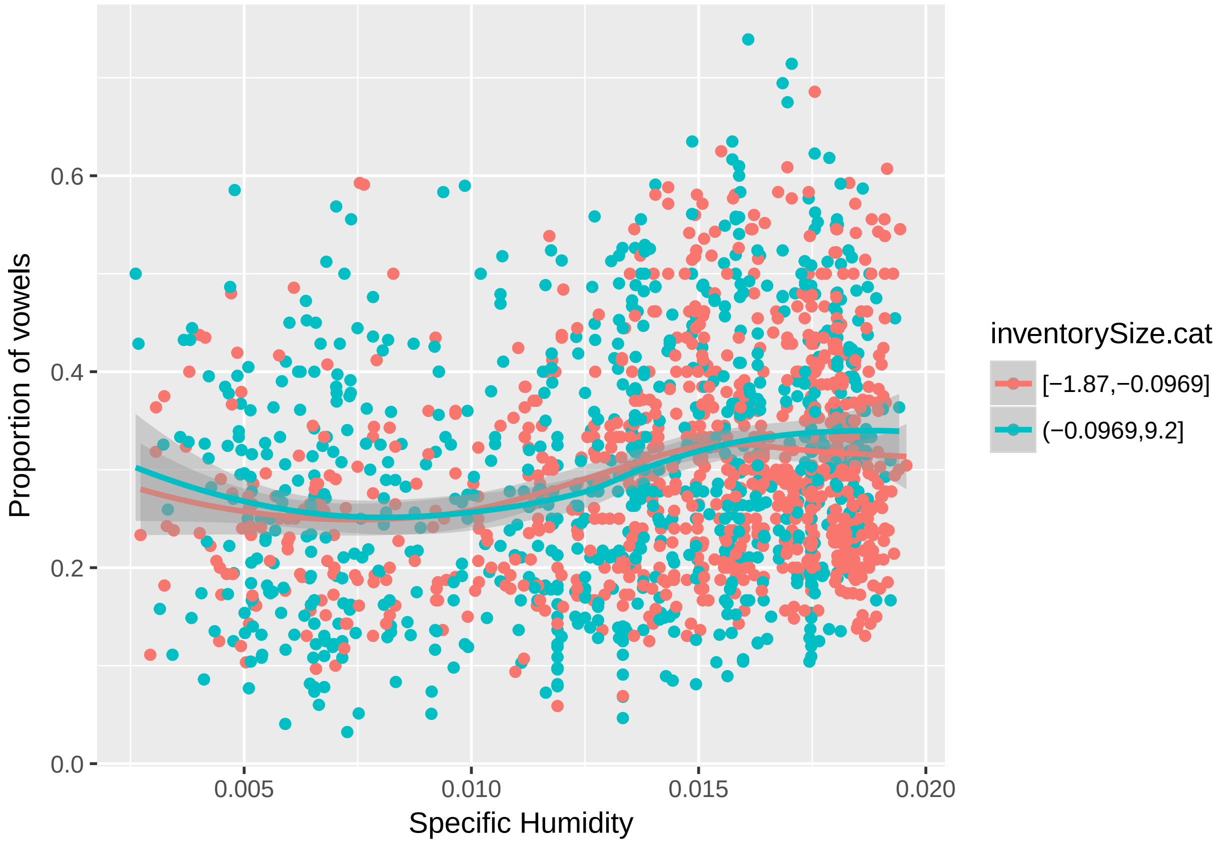

Mixed effects models

We run mixed effects models predicting the number proportion of vowels to consonants (`prop.vowels.scaled`). We start by building a null model with only random effects for language family (`Family`) and geographic area (`autotyp.area`) and random slopes for humidity (`specH.mean.center`). Then we add fixed effects for the inventory size (`inventorySize`), the humidity and the interaction between the two.

```
m0 = lmer(prop.vowels.scaled ~ 1 +
            (1+specH.mean.center|Family) +
            (1+specH.mean.center|autotyp.area),
          data=p)

m1 = lmer(prop.vowels.scaled ~ 1 +
            inventorySize +
            (1+specH.mean.center|Family) +
            (1+specH.mean.center|autotyp.area),
          data=p)

m2 = lmer(prop.vowels.scaled ~
            specH.mean.center +
            inventorySize +
            (1+specH.mean.center|Family) +
            (1+specH.mean.center|autotyp.area),
          data=p)
```

```r
m3 = lmer(prop.vowels.scaled ~
            inventorySize *
            specH.mean.center +
            (1+specH.mean.center|Family) +
            (1+specH.mean.center|autotyp.area),
          data=p)
```

Test the contribution of humidity:

```r
anova(m0,m1,m2,m3)
```

```
## refitting model(s) with ML (instead of REML)
```

```
## Data: p
## Models:
## m0: prop.vowels.scaled ~ 1 + (1 + specH.mean.center | Family) + (1 +
## m0:     specH.mean.center | autotyp.area)
## m1: prop.vowels.scaled ~ 1 + inventorySize + (1 + specH.mean.center |
## m1:     Family) + (1 + specH.mean.center | autotyp.area)
## m2: prop.vowels.scaled ~ specH.mean.center + inventorySize + (1 +
## m2:     specH.mean.center | Family) + (1 + specH.mean.center | autotyp.area)
## m3: prop.vowels.scaled ~ inventorySize * specH.mean.center + (1 +
## m3:     specH.mean.center | Family) + (1 + specH.mean.center | autotyp.area)
##    Df    AIC    BIC  logLik deviance   Chisq Chi Df Pr(>Chisq)
## m0  8 4441.9 4485.6 -2213.0   4425.9
## m1  9 4440.1 4489.2 -2211.1   4422.1  3.8211      1   0.050611 .
## m2 10 4434.4 4488.9 -2207.2   4414.4  7.7654      1   0.005326 **
## m3 11 4417.8 4477.8 -2197.9   4395.8 18.5657      1  1.641e-05 ***
## ---
## Signif. codes:  0 '***' 0.001 '**' 0.01 '*' 0.05 '.' 0.1 ' ' 1
```

```r
summary(m3)
```

```
## Linear mixed model fit by REML ['lmerMod']
## Formula: prop.vowels.scaled ~ inventorySize * specH.mean.center + (1 +
##     specH.mean.center | Family) + (1 + specH.mean.center | autotyp.area)
##    Data: p
##
## REML criterion at convergence: 4414.6
##
## Scaled residuals:
##     Min      1Q  Median      3Q     Max
## -2.9282 -0.6815 -0.1059  0.6071  3.6261
##
## Random effects:
##  Groups       Name              Variance Std.Dev. Corr
##  Family       (Intercept)       0.240004 0.48990
##               specH.mean.center 0.015998 0.12648  0.21
##  autotyp.area (Intercept)       0.061230 0.24745
##               specH.mean.center 0.008478 0.09207  -1.00
##  Residual                       0.666153 0.81618
## Number of obs: 1730, groups:  Family, 165; autotyp.area, 24
##
## Fixed effects:
##                                  Estimate Std. Error t value
## (Intercept)                       0.06052    0.07916   0.765
```

```
## inventorySize                     0.09593    0.02674   3.587
## specH.mean.center                 0.16820    0.04639   3.626
## inventorySize:specH.mean.center  0.10425    0.02381   4.379
##
## Correlation of Fixed Effects:
##             (Intr) invntS spcH..
## inventorySz  0.141
## spcH.mn.cnt -0.135  0.141
## invntrS:H..  0.158  0.342  0.080
```

Note that random slope and intercept for area are exactly correlated. Removing the random slope (or the intercept) just makes the relationship very slighlty weaker, though slighly more significant. Essentially, there is little difference:

```
m1b = lmer(prop.vowels.scaled ~ 1 +
            inventorySize +
            (1+specH.mean.center|Family) +
            (1|autotyp.area),
          data=p)

m2b = lmer(prop.vowels.scaled ~
            specH.mean.center +
            inventorySize +
            (1+specH.mean.center|Family) +
            (1|autotyp.area),
          data=p)
anova(m1b,m2b)
```

```
## refitting model(s) with ML (instead of REML)
```

```
## Data: p
## Models:
## m1b: prop.vowels.scaled ~ 1 + inventorySize + (1 + specH.mean.center |
## m1b:     Family) + (1 | autotyp.area)
## m2b: prop.vowels.scaled ~ specH.mean.center + inventorySize + (1 +
## m2b:     specH.mean.center | Family) + (1 | autotyp.area)
##     Df    AIC    BIC  logLik deviance  Chisq Chi Df Pr(>Chisq)
## m1b  7 4443.5 4481.7 -2214.8   4429.5
## m2b  8 4436.6 4480.2 -2210.3   4420.6 8.9831      1   0.002725 **
## ---
## Signif. codes:  0 '***' 0.001 '**' 0.01 '*' 0.05 '.' 0.1 ' ' 1
```

```
summary(m2b)
```

```
## Linear mixed model fit by REML ['lmerMod']
## Formula: prop.vowels.scaled ~ specH.mean.center + inventorySize + (1 +
##     specH.mean.center | Family) + (1 | autotyp.area)
##    Data: p
##
## REML criterion at convergence: 4433.5
##
## Scaled residuals:
##     Min      1Q  Median      3Q     Max
## -2.5693 -0.6913 -0.1083  0.6070  3.5741
##
## Random effects:
```

```
##  Groups       Name             Variance Std.Dev. Corr
##  Family       (Intercept)      0.23582  0.4856
##               specH.mean.center 0.02716  0.1648   -0.03
##  autotyp.area (Intercept)      0.07729  0.2780
##  Residual                      0.67340  0.8206
## Number of obs: 1730, groups:  Family, 165; autotyp.area, 24
##
## Fixed effects:
##                     Estimate Std. Error t value
## (Intercept)       -0.003818   0.084537  -0.045
## specH.mean.center  0.145493   0.047227   3.081
## inventorySize      0.055007   0.025470   2.160
##
## Correlation of Fixed Effects:
##             (Intr) spcH..
## spcH.mn.cnt 0.170
## inventorySz 0.090  0.137
```

Summary:

There was a significant main effect of humidity (beta = 0.17 , log likelihood difference = 3.9 , df = 1 , Chi Squared = 7.77 , p = 0.0053).

There was a significant interaction between humidity and inventory size (beta = 0.1 , log likelihood difference = 9.3 , df = 1 , Chi Squared = 18.57 , p = 1.6e-05).

Plots

Plot the model estimates:

```
x = sjp.lmer(m3,'fe.slope',
             vars=c("specH.mean.center"),
             show.scatter = T, show.ci = T,
             prnt.plot = F)
```

```
## Warning: Insufficient length of color palette provided. 2 color values
## needed.
```

```
## Warning: Interaction terms are not supported by this plot type. Output for
## interaction terms may be inappropriate.
```

```
# Rescale humidity back to real values
x$plot.list[[1]]$data$x =
  p[complete.cases(
    p[,c("specH.mean.center",
         'prop.vowels.scaled','Family',
         'autotyp.area')]),]$specH.mean

# rescale proportion of vowels to real values
x$plot.list[[1]]$data$y =
  x$plot.list[[1]]$data$y*
  attr(p$prop.vowels.scaled,'scaled:scale') +
  attr(p$prop.vowels.scaled, 'scaled:center')
```

```
mx = x$plot.list[[1]] +
  xlab("Specific Humidity")+
  ylab("Vowel Index")

mx
```

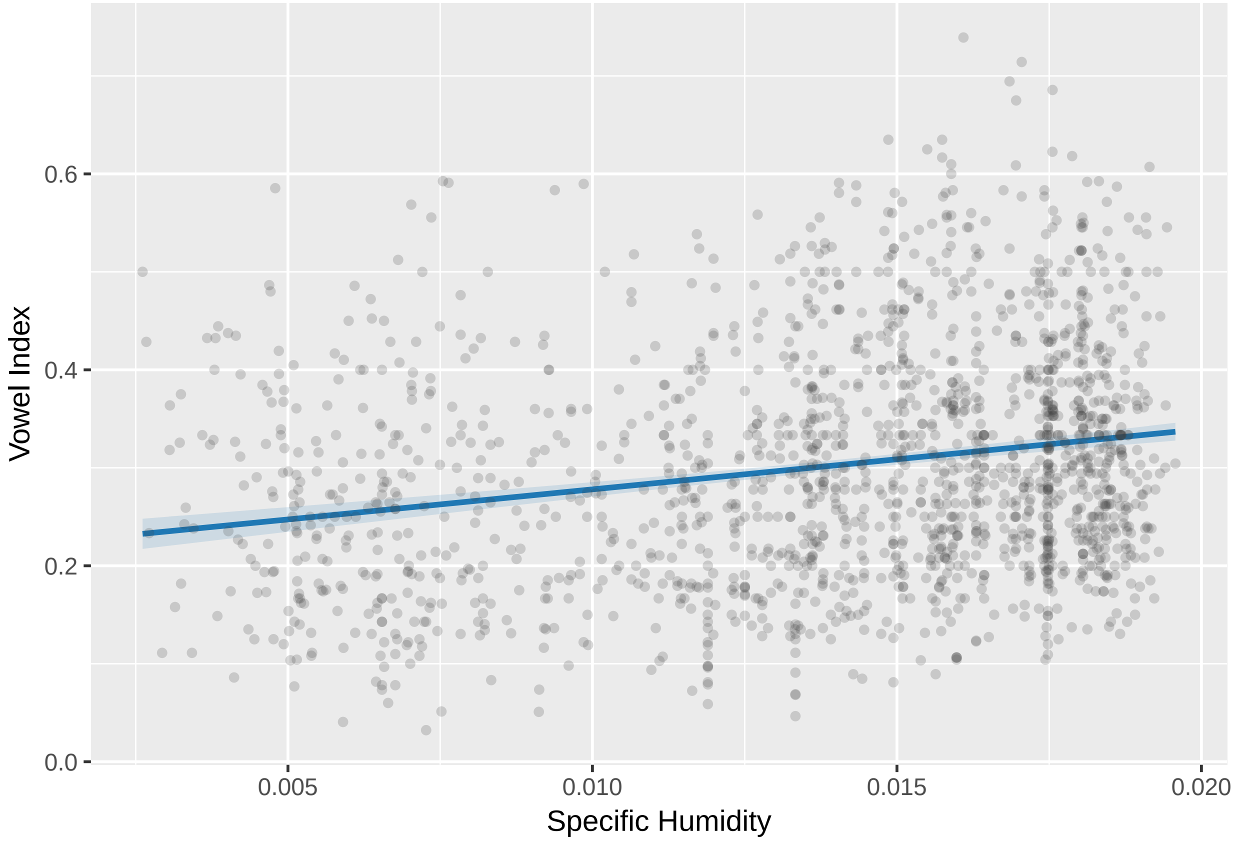


```
# write to file
pdf("../results/PropVowels_SpecH_Estimates.pdf", width=4, height=4)
mx
dev.off()
```

```
## pdf
##   2
```

Random effects:

```
sjp.lmer(m3, 're', sort.est = c("(Intercept)"))
```

```
## Plotting random effects...
## Plotting random effects...
```

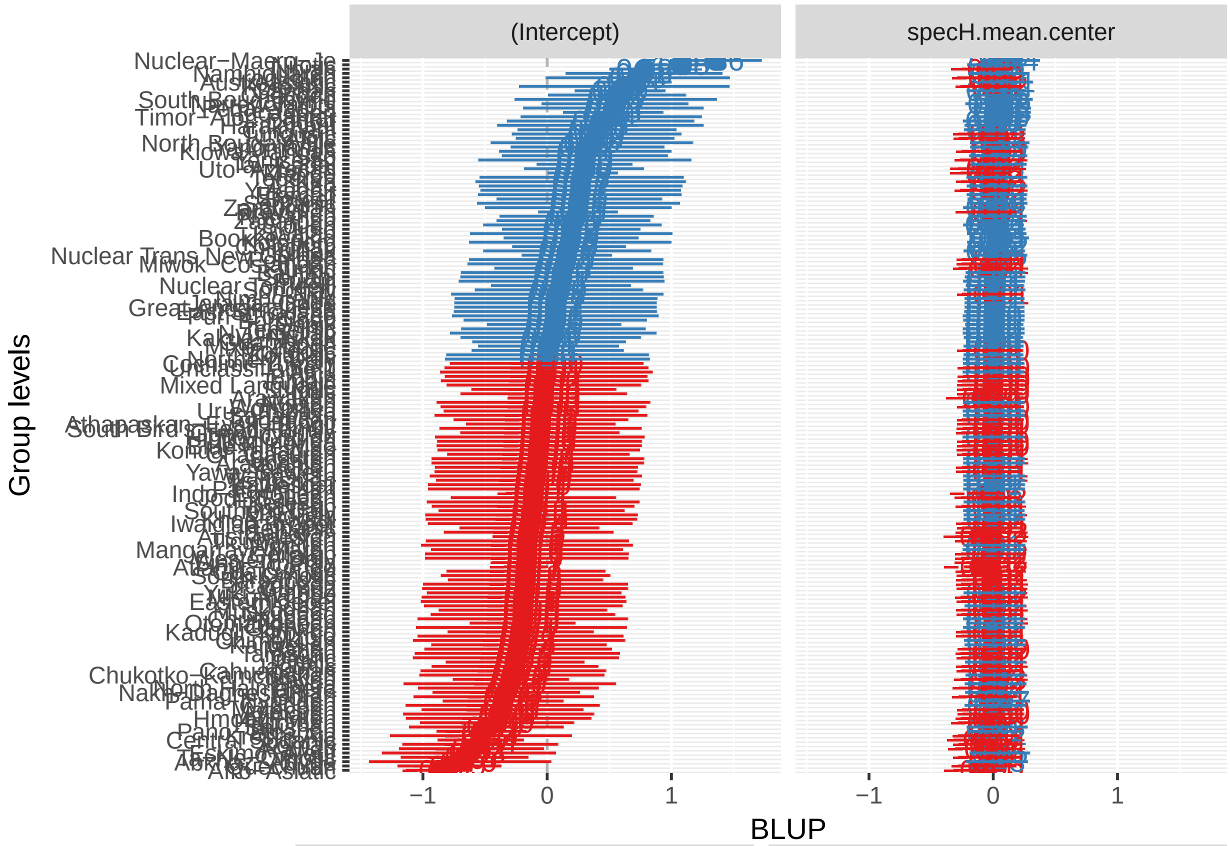

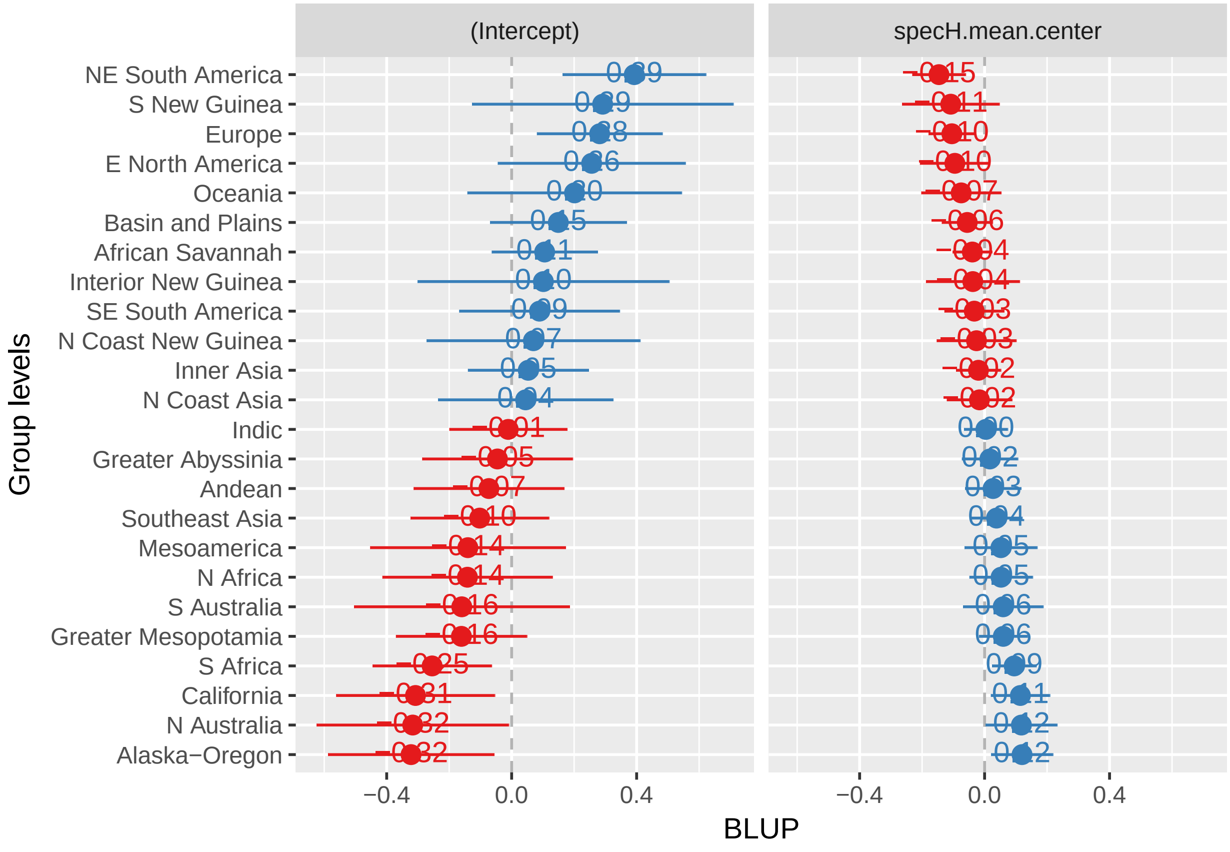

Plot the interaction:

```r
x = sjp.int(m3, show.ci = T, prnt.plot = F)

x$plot.list[[1]]$data$x =
  x$plot.list[[1]]$data$x*
  attr(p$specH.mean.center,"scaled:scale") +
  attr(p$specH.mean.center,"scaled:center")

x$plot.list[[1]]$data$x =
  ((x$plot.list[[1]]$data$x*h.lambda) + 1)^(1/h.lambda)

# rescale proportion of vowels to real values
x$plot.list[[1]]$data$y =
  x$plot.list[[1]]$data$y*
  attr(p$prop.vowels.scaled,'scaled:scale') +
  attr(p$prop.vowels.scaled, 'scaled:center')

x$plot.list[[1]]$data$conf.low =
  x$plot.list[[1]]$data$conf.low*
  attr(p$prop.vowels.scaled,'scaled:scale') +
  attr(p$prop.vowels.scaled, 'scaled:center')

x$plot.list[[1]]$data$conf.high =
  x$plot.list[[1]]$data$conf.high*
  attr(p$prop.vowels.scaled,'scaled:scale') +
  attr(p$prop.vowels.scaled, 'scaled:center')

grp = as.numeric(as.character(x$plot.list[[1]]$data$grp))
x$plot.list[[1]]$data$grp = "Smallest inventory"
x$plot.list[[1]]$data$grp[grp>0] = "Largest inventory"
x$plot.list[[1]]$data$grp = as.factor(x$plot.list[[1]]$data$grp)

x$plot.list[[1]]$coordinates$limits$x = range(x$plot.list[[1]]$data$x)
x$plot.list[[1]]$coordinates$limits$y = range(x$plot.list[[1]]$data$y)

x$plot.list[[1]]$labels$colour = "Inventory Size"

intx = x$plot.list[[1]] + xlab("Humidity") +
  ylab("Predicted values of proportion of vowels") +
  theme(plot.title=element_blank()) +
  scale_colour_discrete("Inventory size")
```

```
## Scale for 'colour' is already present. Adding another scale for
## 'colour', which will replace the existing scale.
```

```r
intx
```

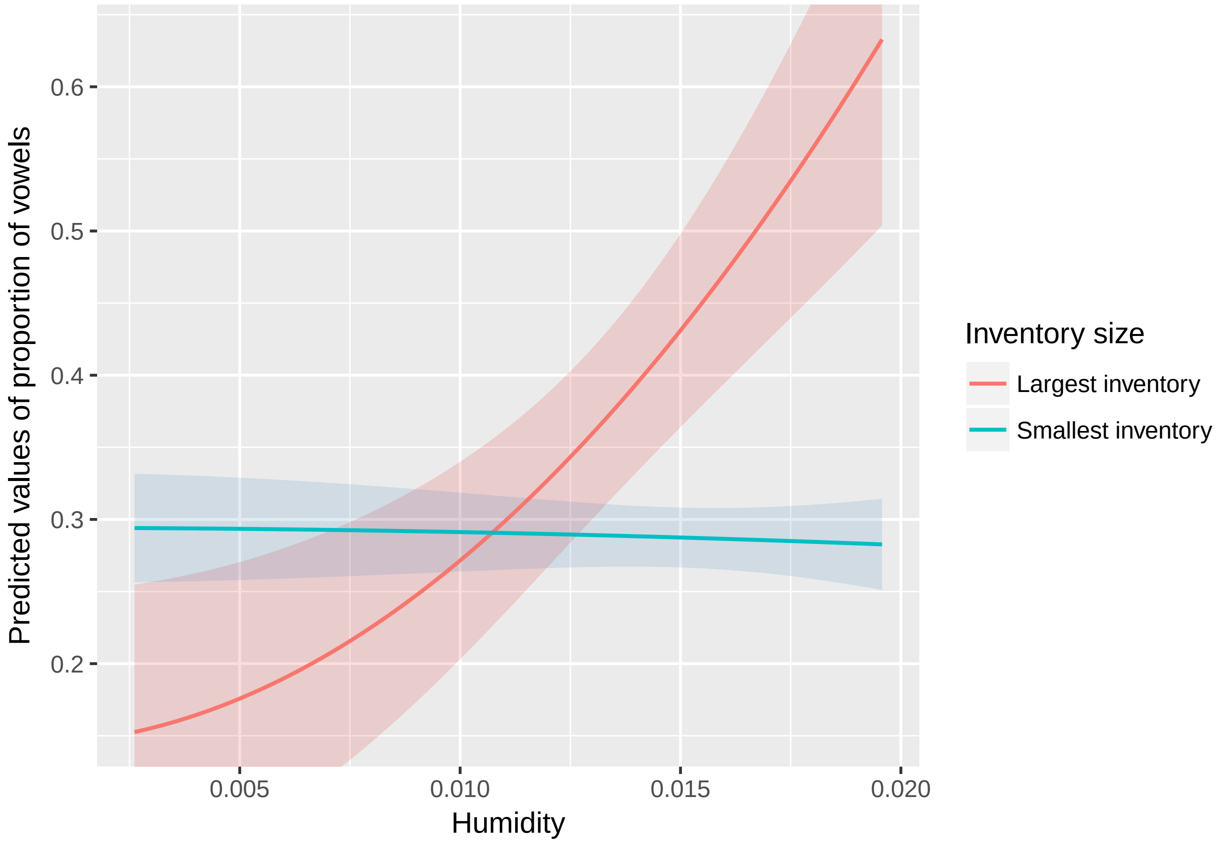


```
pdf("../results/PropVowels_SpecH_Interaction.pdf",
    width=6, height=4)
intx
dev.off()
```

```
## pdf
##   2
```

Testing the optimiser parameter robustness

From https://raw.githubusercontent.com/lme4/lme4/master/inst/utils/allFit.R

```
source("allFit.R")
aa <- allFit(m3)
```

```
## bobyqa : [OK]
## Nelder_Mead : [OK]
## nlminbw : [OK]
## nmkbw : [OK]
## optimx.L-BFGS-B : [OK]
## nloptwrap.NLOPT_LN_NELDERMEAD : [OK]
## nloptwrap.NLOPT_LN_BOBYQA : [OK]
```

Look at differences in log likelihood (very small differences):

```
t(t(lliks <- sort(sapply(aa,logLik))))
```

```
##                                      [,1]
## nloptwrap.NLOPT_LN_NELDERMEAD -2207.397
```

```
## nloptwrap.NLOPT_LN_BOBYQA      -2207.397
## nlminbw                        -2207.397
## optimx.L-BFGS-B                -2207.397
## nmkbw                          -2207.308
## Nelder_Mead                    -2207.308
## bobyqa                         -2207.308
```

Differences in parameter estimates. The plots below show the differences for each coefficient in model 3 when using a different optimiser. The differences are very small, and none change the sign of the parameter.

```
aa.fixef <- t(sapply(aa,fixef))
aa.fixef.m <- melt(aa.fixef)
models <- levels(aa.fixef.m$Var1)
ylabs <- substr(models,1,3)
aa.fixef.m <- transform(aa.fixef.m,Optimiser=factor(Var1,levels=names(lliks)))
ggplot(aa.fixef.m,aes(x=value,y=Optimiser,colour=Optimiser))+
  geom_point()+
  facet_wrap(~Var2,scale="free")+
  scale_colour_brewer(palette="Dark2")+
  scale_y_discrete(breaks=models,
                   labels=ylabs)+
  theme(legend.position='top') +
  labs(x="",y="")
```

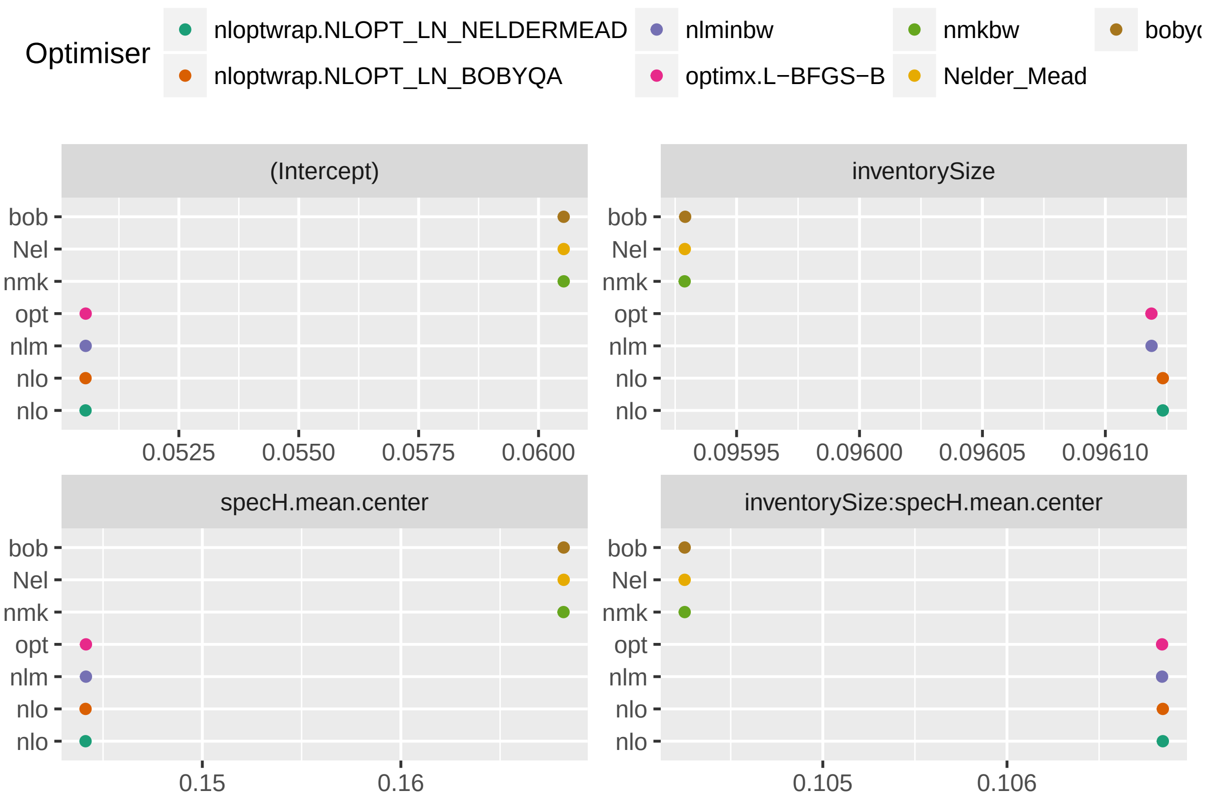

MCMCglmm

Run the same model as above, but with the MCMCglmm package.

Set up the priors:

```
familyRandomEffectsN = 2
areaRandomEffectsN = 2

prior.m3 <- list(
  R=list(V=1, n=1, fix=1),
  G=list(G1=list(V        = diag(familyRandomEffectsN),  # family intercept+slope
     n        = familyRandomEffectsN,
     alpha.mu = rep(0, familyRandomEffectsN),
     alpha.V  = diag(familyRandomEffectsN)*25^2),
G2=list(V        = diag(areaRandomEffectsN), # area intercept+slope
     n        = areaRandomEffectsN,
     alpha.mu = rep(0, areaRandomEffectsN),
     alpha.V  = diag(areaRandomEffectsN)*25^2)))
```

Run the model:

```
set.seed(1234)
m3.mcmcglmm <- MCMCglmm(
  prop.vowels.scaled ~
    specH.mean.center * inventorySize,
  ~ us(1 + specH.mean.center):Family +
    us(1 + specH.mean.center):autotyp.area,
  data    = p,
  family = "gaussian",
  prior   = prior.m3,
  thin    =     10,
  burnin =   1000,
  nitt    = 101000,
  verbose = FALSE)
```

Save the results:

```
save(m3.mcmcglmm, file="../results/m3_mcmcglmm_vowels.RDat")
# load("../results/m3_mcmcglmm_vowels.RDat")
```

Plot the convergence:

```
# Render as png to save space
png("../results/MCMCConvergence_vowels1.png")
plot(m3.mcmcglmm$VCV[,1:3])
dev.off()
```

```
## pdf
##   2
```

```
png("../results/MCMCConvergence_vowels2.png")
plot(m3.mcmcglmm$VCV[,4:6])
dev.off()
```

```
## pdf
##   2
```

```r
png("../results/MCMCConvergence_vowels3.png")
plot(m3.mcmcglmm$VCV[,7:8])
dev.off()
```

```
## pdf
##   2
```

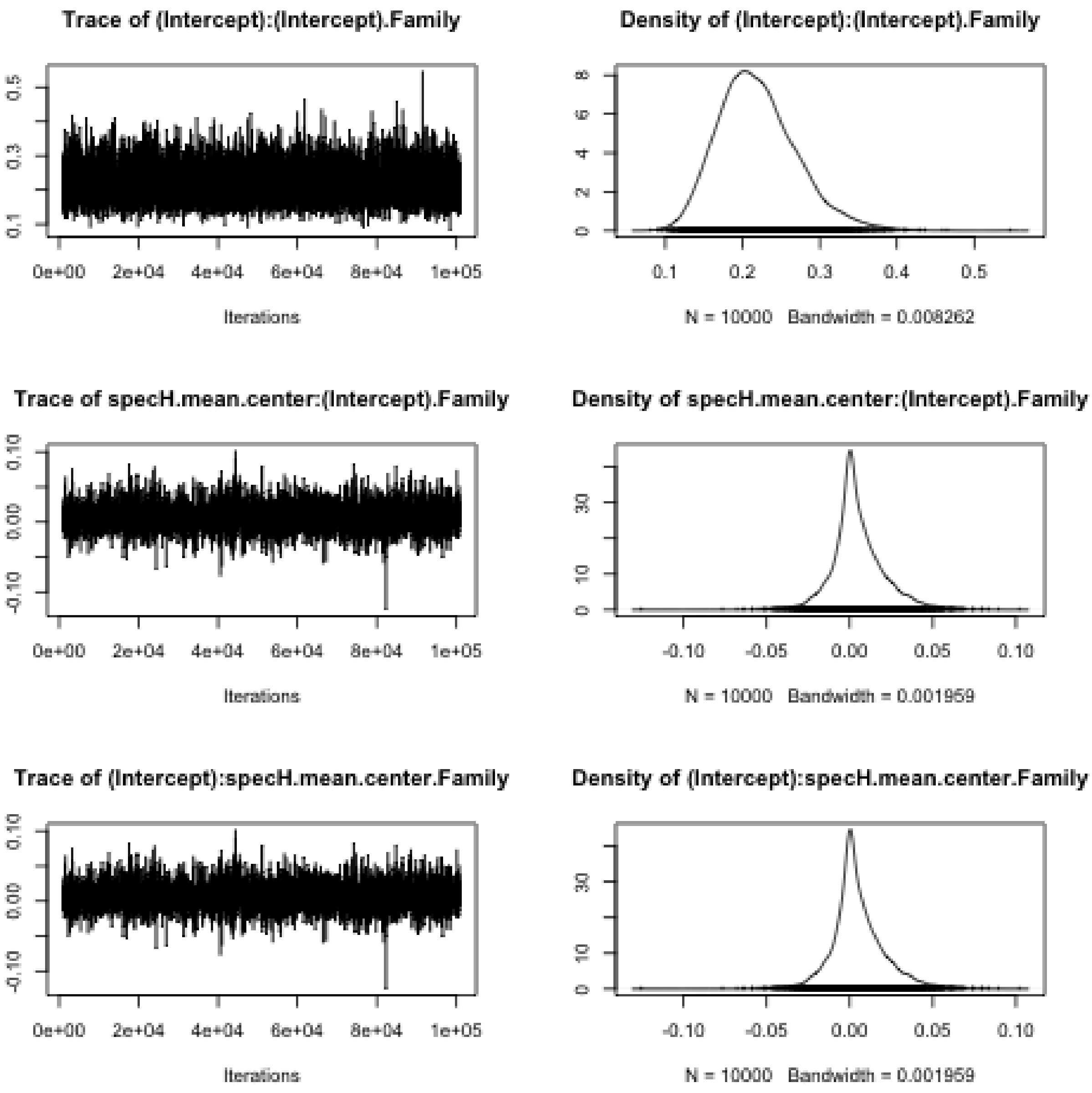

Figure 1:

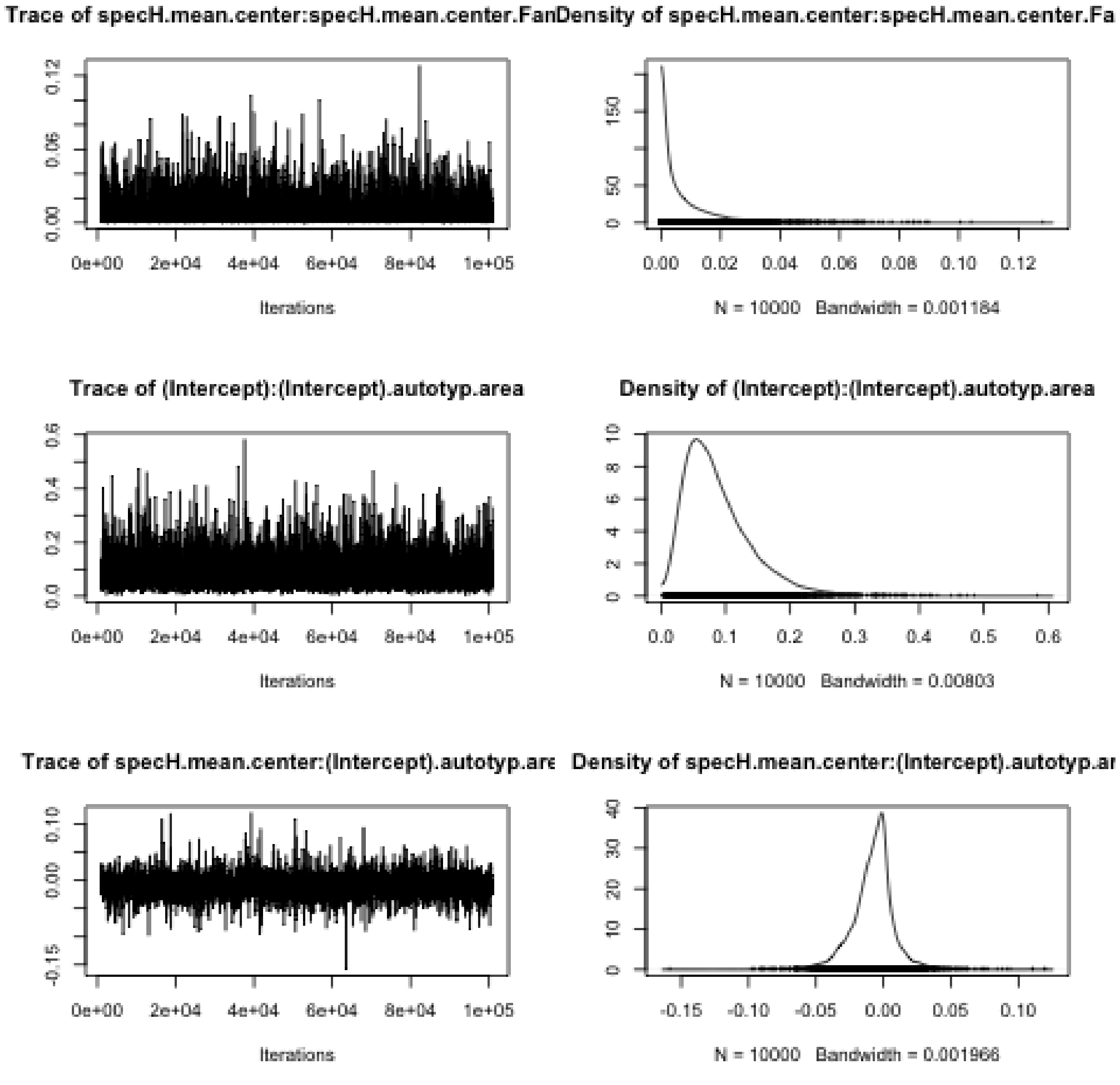
Trace of specH.mean.center:specH.mean.center.Fan
Density of specH.mean.center:specH.mean.center.Fa
Iterations
N = 10000 Bandwidth = 0.001184
Trace of (Intercept):(Intercept).autotyp.area
Density of (Intercept):(Intercept).autotyp.area
Iterations
N = 10000 Bandwidth = 0.00803
Trace of specH.mean.center:(Intercept).autotyp.are
Density of specH.mean.center:(Intercept).autotyp.ar
Iterations
N = 10000 Bandwidth = 0.001966

Figure 2:

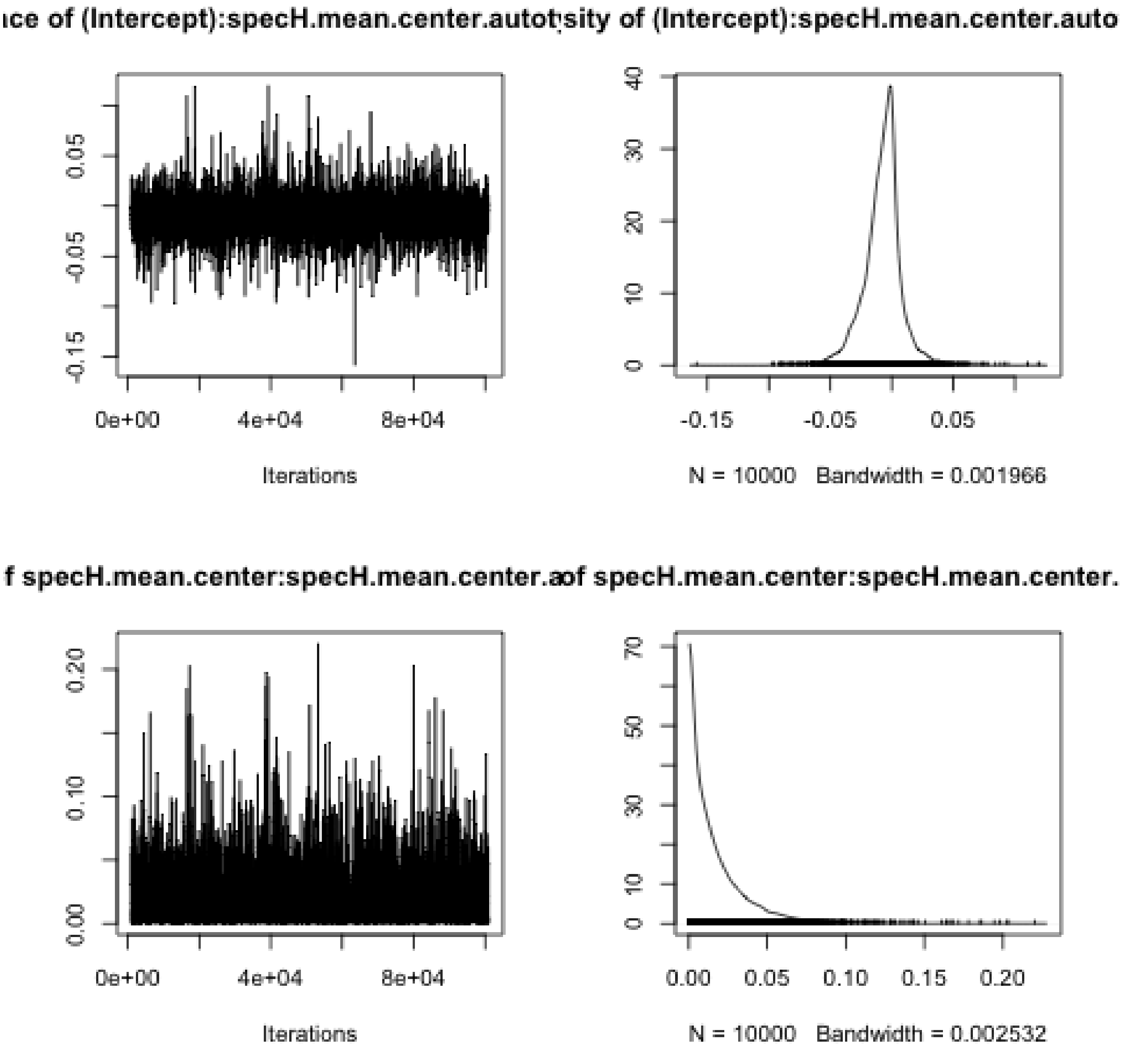
ıce of (Intercept):specH.mean.center.autot;sity of (Intercept):specH.mean.center.auto
0.05
-0.05
-0.15
0e+00
4e+04
8e+04
Iterations
0
10
20
30
40
-0.15
-0.05
0.05
N = 10000 Bandwidth = 0.001966
f specH.mean.center:specH.mean.center.aof specH.mean.center:specH.mean.center.
0.00
0.10
0.20
Iterations
0
10
30
50
70
0.00
0.05
0.10
0.15
0.20
N = 10000 Bandwidth = 0.002532

Figure 3:

```r
sx = summary(m3.mcmcglmm)

sx
```

```
##
##  Iterations = 1001:100991
##  Thinning interval  = 10
##  Sample size  = 10000
##
##  DIC: 4464.137
##
##  G-structure:  ~us(1 + specH.mean.center):Family
##
##                                             post.mean  l-95% CI u-95% CI
## (Intercept):(Intercept).Family               0.219163  1.28e-01  0.32195
## specH.mean.center:(Intercept).Family         0.004919 -2.49e-02  0.04125
## (Intercept):specH.mean.center.Family         0.004919 -2.49e-02  0.04125
## specH.mean.center:specH.mean.center.Family  0.007724  1.21e-12  0.02978
##                                             eff.samp
## (Intercept):(Intercept).Family                  8880
## specH.mean.center:(Intercept).Family            6128
## (Intercept):specH.mean.center.Family            6128
## specH.mean.center:specH.mean.center.Family     7625
##
##                ~us(1 + specH.mean.center):autotyp.area
##
##                                                   post.mean   l-95% CI
## (Intercept):(Intercept).autotyp.area               0.089448  7.714e-03
## specH.mean.center:(Intercept).autotyp.area        -0.007713 -4.304e-02
## (Intercept):specH.mean.center.autotyp.area        -0.007713 -4.304e-02
## specH.mean.center:specH.mean.center.autotyp.area  0.017428  1.344e-10
##                                                   u-95% CI eff.samp
## (Intercept):(Intercept).autotyp.area               0.19676     4906
## specH.mean.center:(Intercept).autotyp.area         0.02408     7321
## (Intercept):specH.mean.center.autotyp.area         0.02408     7321
## specH.mean.center:specH.mean.center.autotyp.area  0.05704     6023
##
##  R-structure:  ~units
##
##       post.mean l-95% CI u-95% CI eff.samp
## units         1        1        1        0
##
##  Location effects: prop.vowels.scaled ~ specH.mean.center * inventorySize
##
##                                  post.mean l-95% CI u-95% CI eff.samp
## (Intercept)                        0.04994 -0.12856  0.23583    10000
## specH.mean.center                  0.16097  0.04526  0.27500     9002
## inventorySize                      0.09409  0.03001  0.15641    10000
## specH.mean.center:inventorySize   0.10836  0.05251  0.16695    10000
##                                   pMCMC
## (Intercept)                      0.5766
## specH.mean.center                0.0042 **
## inventorySize                    0.0032 **
## specH.mean.center:inventorySize <1e-04 ***
```

```
## ---
## Signif. codes:  0 '***' 0.001 '**' 0.01 '*' 0.05 '.' 0.1 ' ' 1
```

```
fe = m3.mcmcglmm$Sol[,2]
dx = density(fe)
plot(dx, main='',
     xlab='Parameter estimate\nfor humidity')
abline(v=0)
```

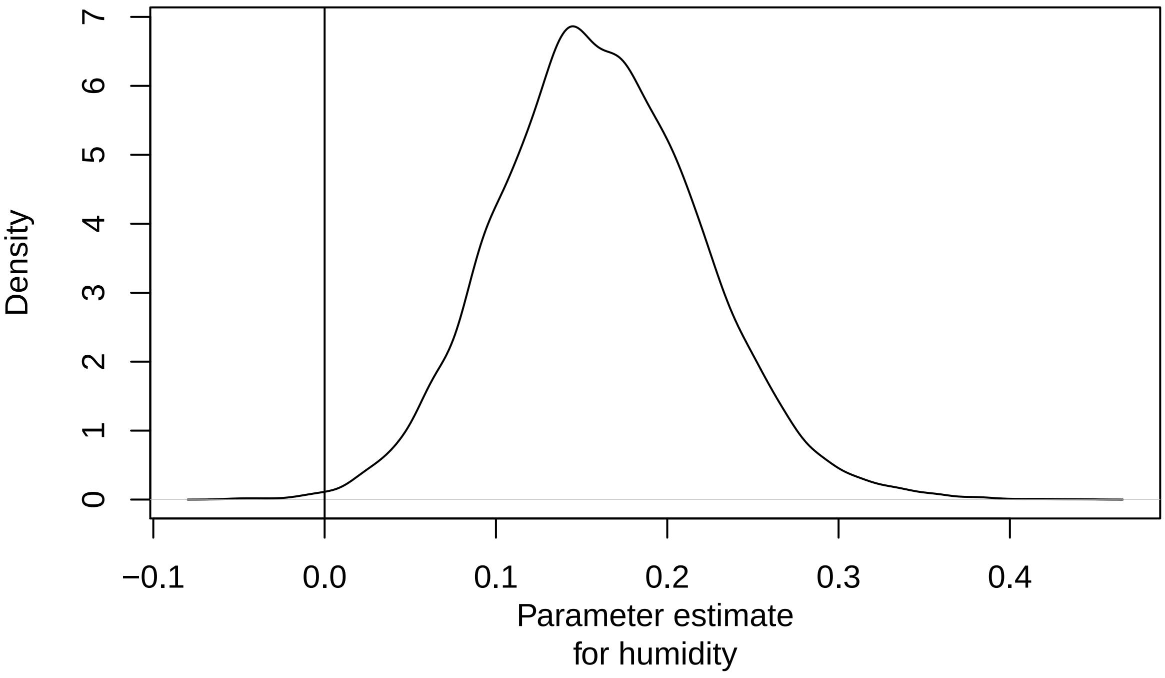


```
fe2 = m3.mcmcglmm$Sol[,3]
dx2 = density(fe2)
plot(dx2, main='',
     xlab='Parameter estimate\nfor inventory size')
abline(v=0)
```

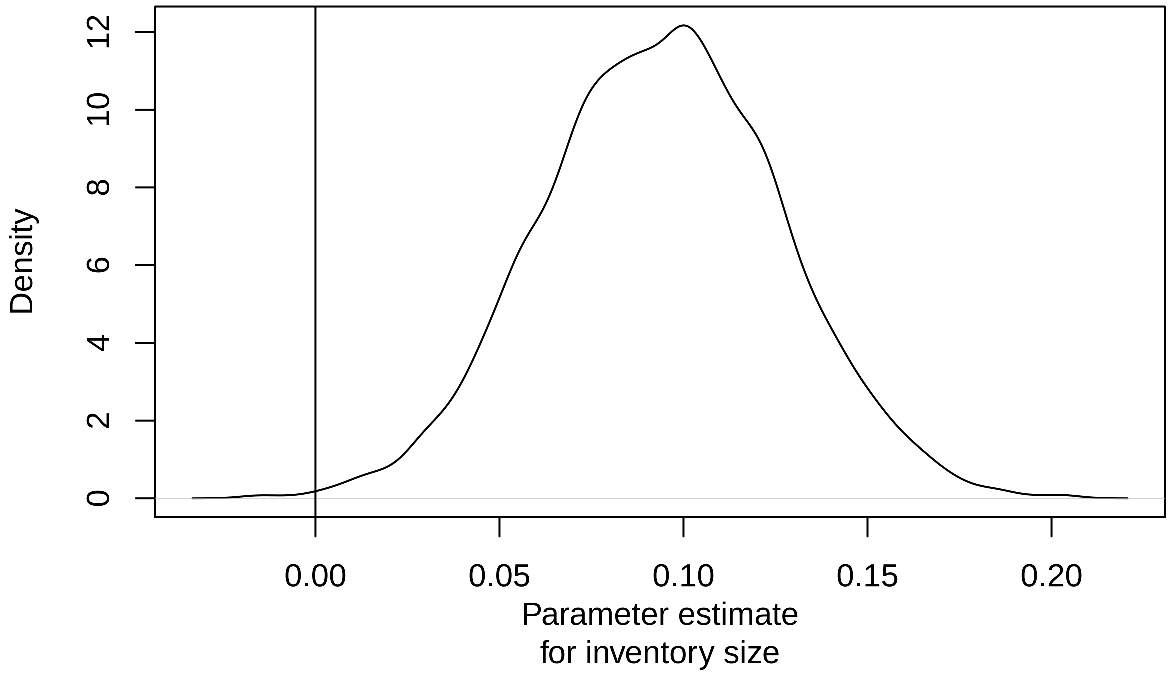


```
re = m3.mcmcglmm$VCV
re = as.data.frame(re)
re.area = re$`specH.mean.center:specH.mean.center.autotyp.area`
re.area.d = density(re.area)
plot(re.area.d)
```

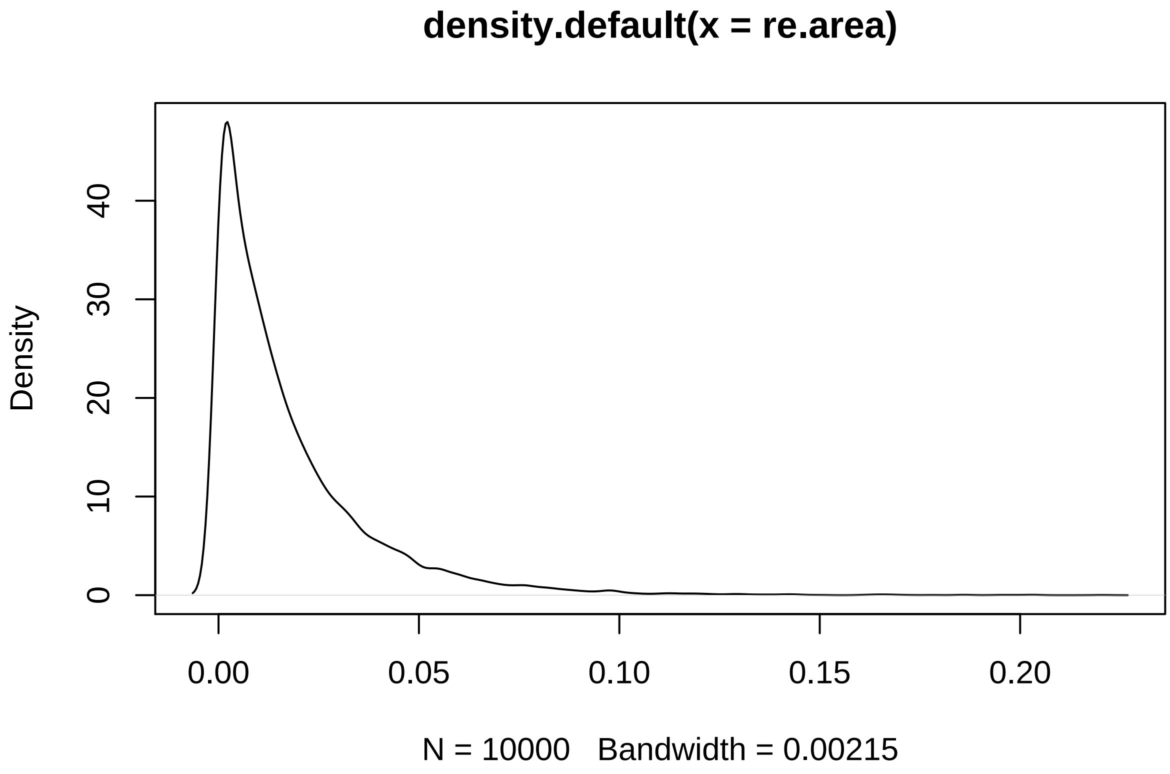

Supplement: Supplementary file 3 [file DataSheet3.PDF]
