# Supplementary material for: Robust, Causal, and Incremental Approaches to Investigating Linguistic Adaptation
Source: Front Psychol. 2018 Feb 21;9:166. doi: 10.3389/fpsyg.2018.00166 (PMC5826341; doi:10.3389/fpsyg.2018.00166)
Supplement: Supplementary file 4 [file DataSheet4.PDF]

Vowel area and humidity

Load libraries

```
library(ggplot2)
library(lme4)
library(sjPlot)
```

Load data

The data comes from Becker's (2010) corpus of vowel measurements (the script to wrangle the data came from Marton Soskuthy). For each language, the F1 and F2 measures of all vowels were taken, then the area of the convex hull of the points was calculated. This is the 'area' that a vowel system takes up.

We hypothesised that vowel areas are more restricted in drier climates.

```
d  = read.csv("../data/BeckerVowelCorpus_Area.csv", stringsAsFactors = F)

ggplot(d, aes(x=specH.mean, y = area)) +
  geom_point() + geom_smooth(method='lm')
```

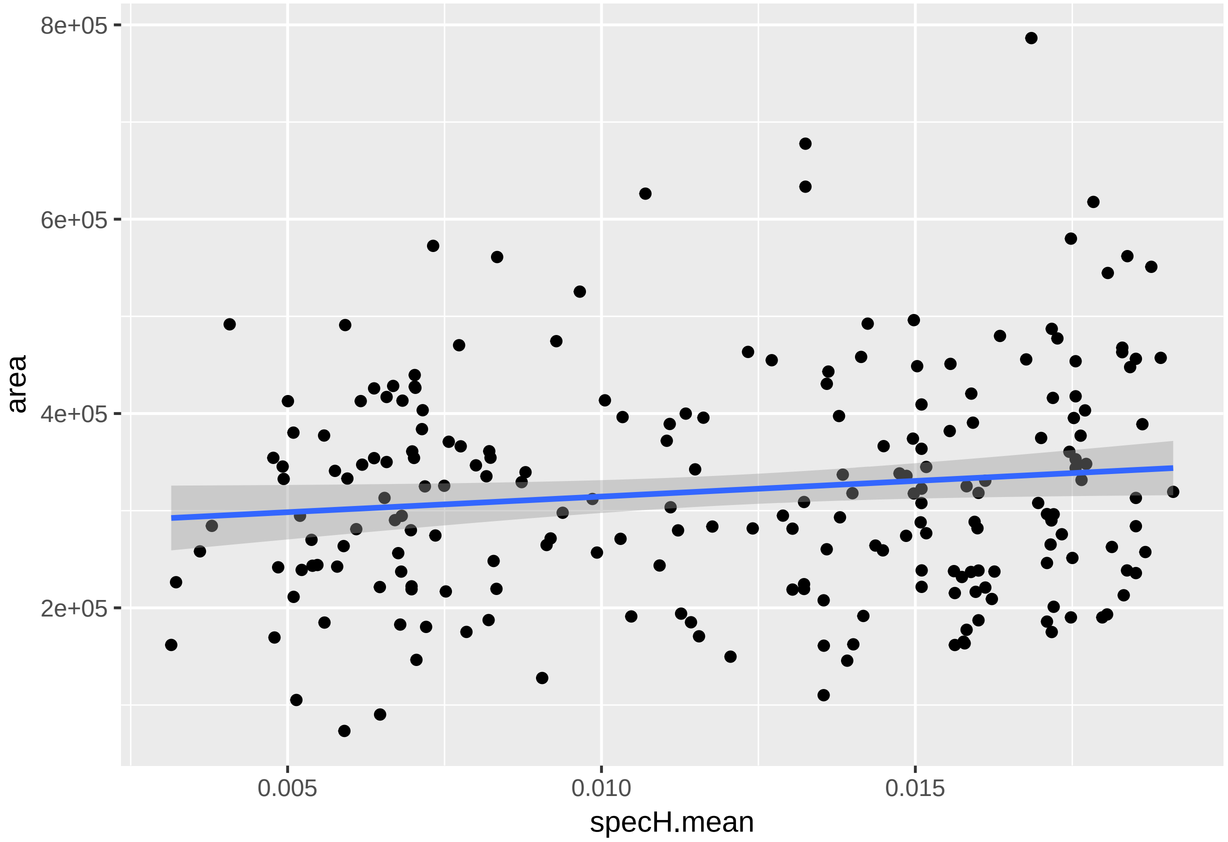


```
d$area.scaled = scale(d$area)
d$specH.mean.scled = scale(d$specH.mean)
```

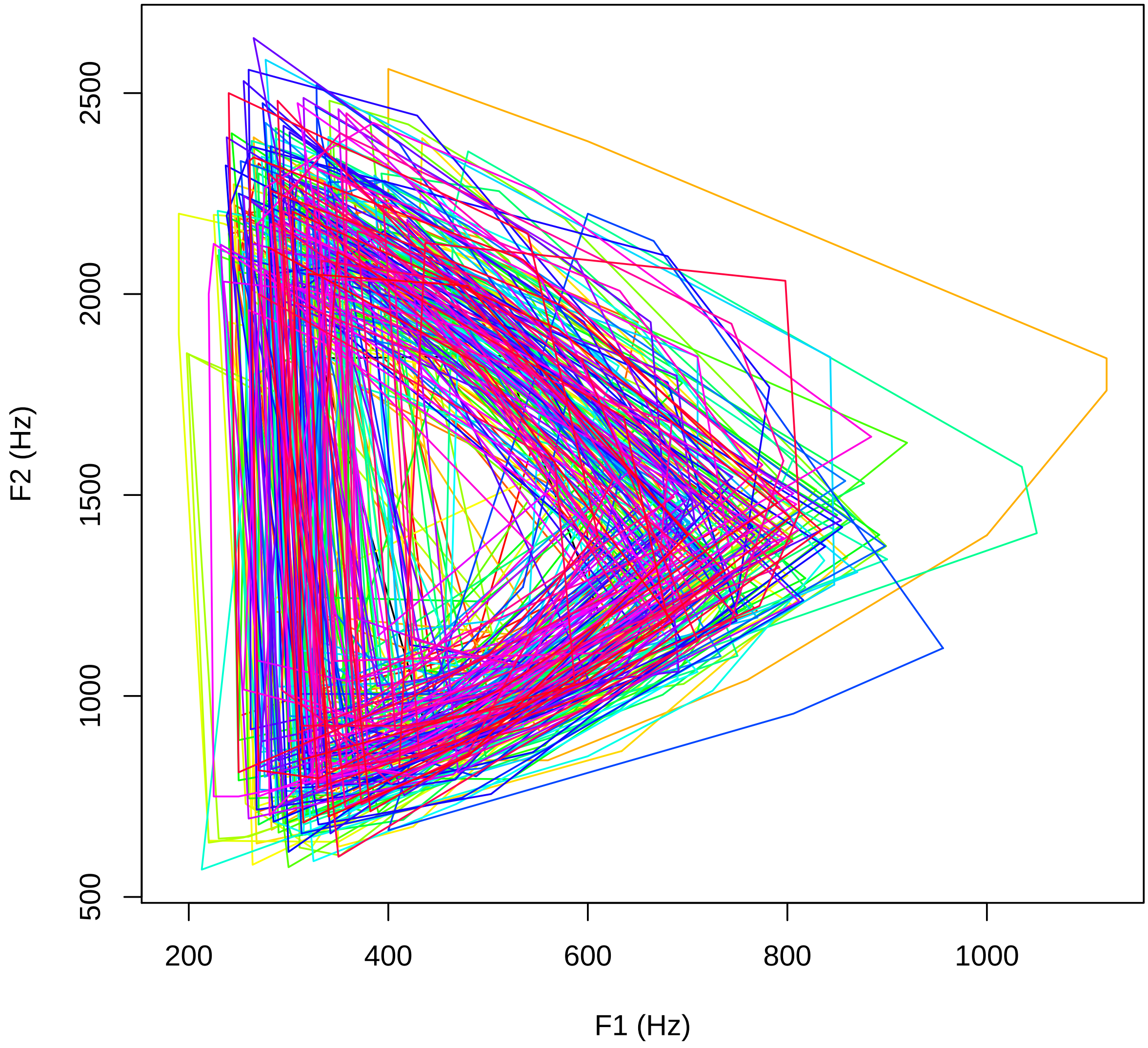

Figure 1: Polygons for the convex hull of the F1 and F2 measures for langauges from the Becker corpus.

Mixed effects modelling

The full model structure, including slopes:

```
m0.full = lmer(area.scaled ~
            1 +
            (1+specH.mean.scled|family) +
            (1+specH.mean.scled|autotyp.area),
          data = d)
```

However, the slopes and intercepts are exactly correlated, suggesting overfitting:

```
cor(ranef(m0.full)$family)
```

```
##                    (Intercept) specH.mean.scled
## (Intercept)                  1                1
## specH.mean.scled             1                1
```

```
cor(ranef(m0.full)$autotyp.area)
```

```
##                    (Intercept) specH.mean.scled
## (Intercept)                  1                1
## specH.mean.scled             1                1
```

Instead, a null model with only intercepts is used. We then add the number of vowels in the system and the mean specific humidity.

```
m0 = lmer(area.scaled ~
            1 +
            (1|family) +
            (1|autotyp.area),
          data = d)

# Add number of vowels:
m1 = lmer(area.scaled ~
            1 + numVowels +
            (1|family) +
            (1|autotyp.area),
          data = d)
# Add humidity:
m2 = lmer(area.scaled ~
            1 + numVowels +
            specH.mean.scled +
            (1|family) +
            (1|autotyp.area),
          data = d)
```

Test the influence of humidity:

```
anova(m0,m1,m2)
```

```
## refitting model(s) with ML (instead of REML)
```

```
## Data: d
## Models:
## m0: area.scaled ~ 1 + (1 | family) + (1 | autotyp.area)
## m1: area.scaled ~ 1 + numVowels + (1 | family) + (1 | autotyp.area)
## m2: area.scaled ~ 1 + numVowels + specH.mean.scled + (1 | family) +
## m2:     (1 | autotyp.area)
```

```
##    Df    AIC    BIC  logLik deviance   Chisq Chi Df Pr(>Chisq)
## m0  4 606.77 620.32 -299.38   598.77
## m1  5 549.04 565.99 -269.52   539.04 59.7236      1  1.092e-14 ***
## m2  6 546.03 566.37 -267.02   534.03  5.0116      1    0.02518 *
## ---
## Signif. codes:  0 '***' 0.001 '**' 0.01 '*' 0.05 '.' 0.1 ' ' 1
```

Plots

Fixed effect estimate:

```
gx = sjp.lmer(m2, "eff", show.ci = T, show.scatter = T,
              prnt.plot = F, facet.grid = F)
```

```
## Warning: package 'bindrcpp' was built under R version 3.3.2
```

```
# Rescale variables
gx$plot.list[[2]]$data$x = (gx$plot.list[[2]]$data$x *attr(d$specH.mean.scled,"scaled:scale")) + attr(d$

gx$plot.list[[2]]$data$y = (gx$plot.list[[2]]$data$y *attr(d$area.scaled,"scaled:scale")) + attr(d$area

gx$plot.list[[2]]$data$lower = (gx$plot.list[[2]]$data$lower *attr(d$area.scaled,"scaled:scale")) + attr

gx$plot.list[[2]]$data$upper = (gx$plot.list[[2]]$data$upper *attr(d$area.scaled,"scaled:scale")) + attr


gxx = gx$plot.list[[2]] +
  theme(plot.title = element_blank(), plot.subtitle = element_blank()) +
  xlab("Specific humidity") +
  ylab("Vowel Area (Hz)") +
  geom_point(data=d, aes(x=specH.mean, y=area), alpha=0.3)
gxx
```

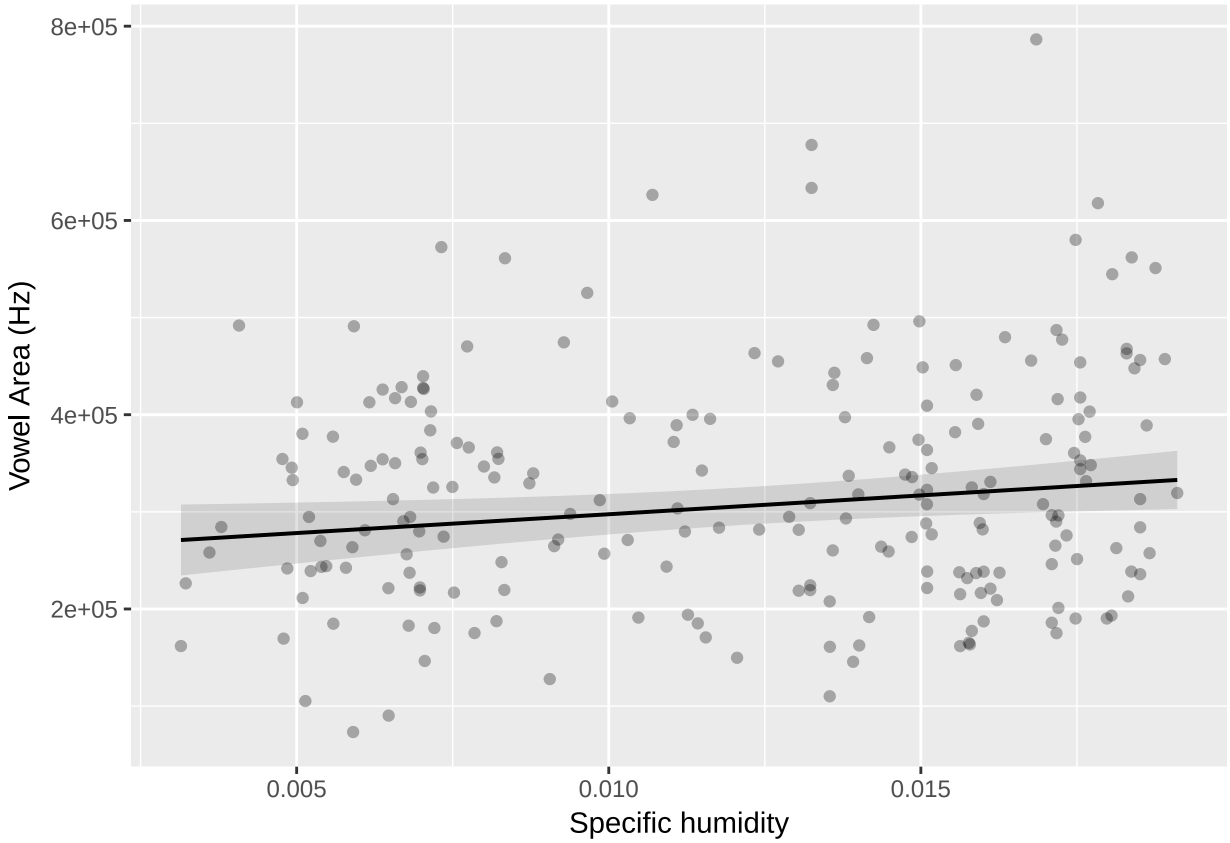


```r
pdf("../results/VowelSpace_and_humidity.pdf",
    width=5, height=5)
gxx
dev.off()
```

```
## pdf
##   2
```

Random effects:

```r
sjp.lmer(m1,'re', sort.est = "(Intercept)")
```

```
## Plotting random effects...
## Plotting random effects...
```

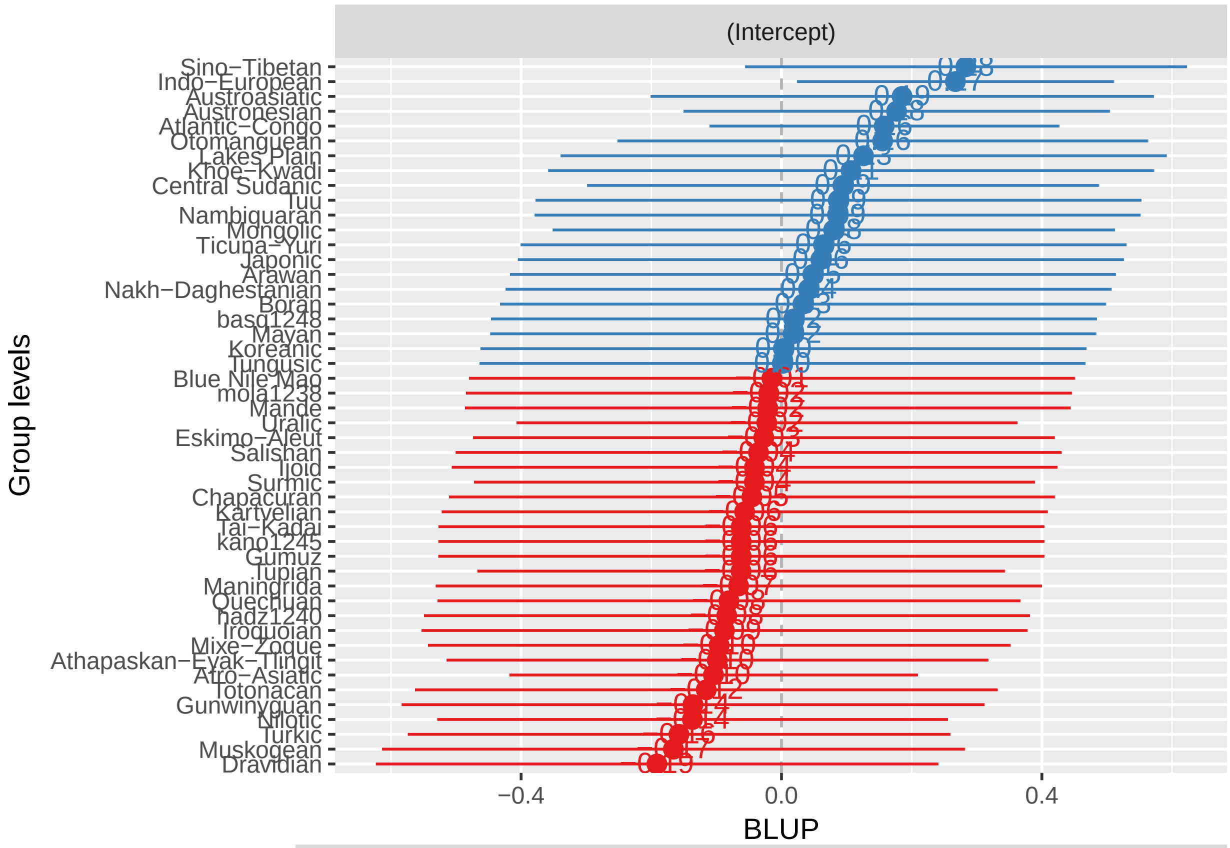
(Intercept)
Group levels
Sino−Tibetan
Indo−European
Austroasiatic
Austronesian
Atlantic−Congo
Otomanguean
Lakes Plain
Khoe−Kwadi
Central Sudanic
Tuu
Nambiquaran
Mongolic
Ticuna−Yuri
Japonic
Arawan
Nakh−Daghestanian
Boran
basq1248
Mayan
Koreanic
Tungusic
Blue Nile Mao
mola1238
Mande
Uralic
Eskimo−Aleut
Salishan
Ijoid
Surmic
Chapacuran
Kartvelian
Tai−Kadai
kano1245
Gumuz
Tupian
Maningrida
Quechuan
hadz1240
Iroquoian
Mixe−Zoque
Athapaskan−Eyak−Tlingit
Afro−Asiatic
Totonacan
Gunwinyguan
Nilotic
Turkic
Muskogean
Dravidian
−0.4
0.0
0.4
BLUP

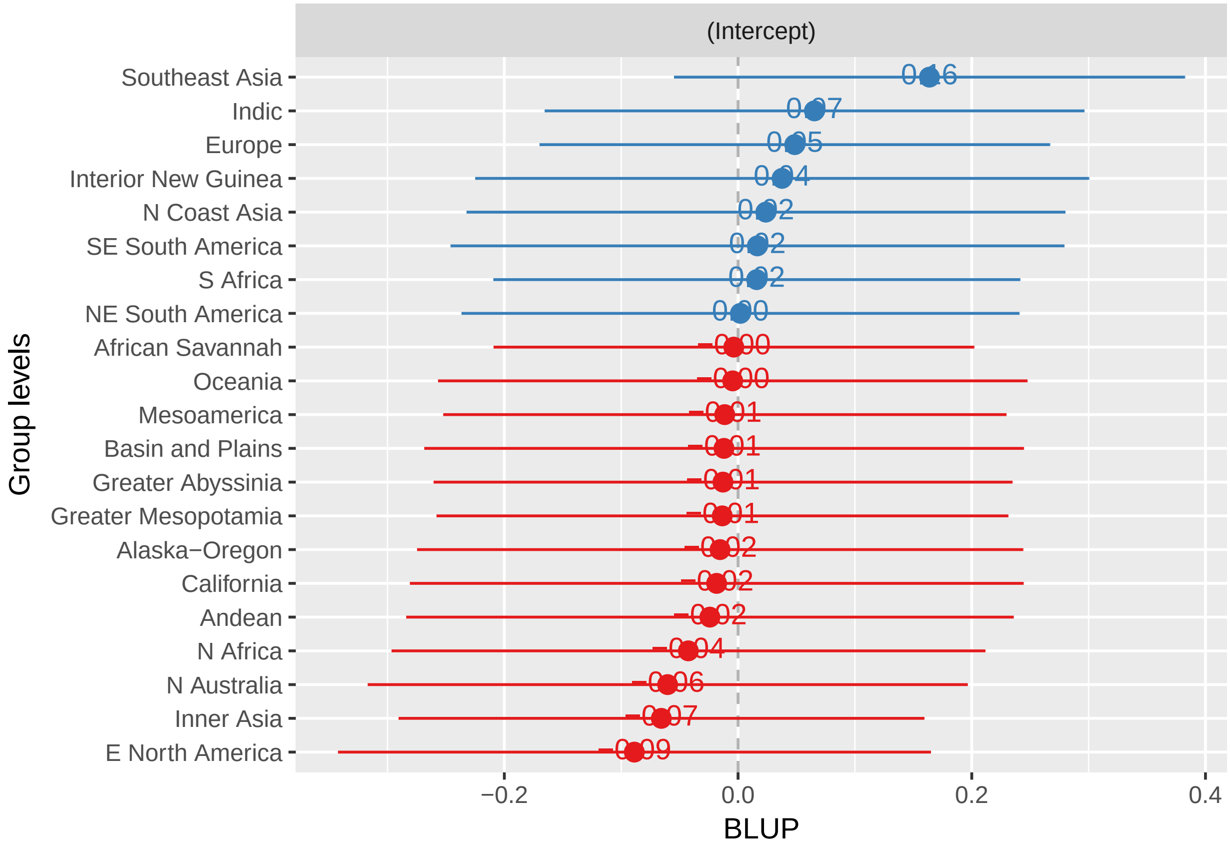
(Intercept)
Group levels
Southeast Asia
Indic
Europe
Interior New Guinea
N Coast Asia
SE South America
S Africa
NE South America
African Savannah
Oceania
Mesoamerica
Basin and Plains
Greater Abyssinia
Greater Mesopotamia
Alaska−Oregon
California
Andean
N Africa
N Australia
Inner Asia
E North America
−0.2
0.0
0.2
0.4
BLUP
