# Supplementary material for: Robust, Causal, and Incremental Approaches to Investigating Linguistic Adaptation
Source: Front Psychol. 2018 Feb 21;9:166. doi: 10.3389/fpsyg.2018.00166 (PMC5826341; doi:10.3389/fpsyg.2018.00166)
Supplement: Supplementary file 5 [file DataSheet5.PDF]

Use of tone in Cantonese throught the year

Load Libraries

```
library(ggplot2)
library(lme4)
```

Load Data

```
d = read.delim("Data/toneCounts.tab",sep='\t',header=T)

d = d[d$corpus!='PaidoCantonese',]

d$date2 = as.Date(d$date,format="%d-%b-%Y")

d$month = as.numeric(format(d$date2, "%m"))
d$summer = d$month %in% c(7:9)
d$season = cut(d$month, c(0,6,9,13))

d = d[!is.na(d$date2),]

d = d[d$language=="yue , eng",]

h = read.delim("Data/SpecificHumidity_HongKong.tsv", skip=1)
head(h)
```

```
##   degree_east degree_north months.since.1960.01.01 unitless
## 1     112.500     21.90444                   360.5  0.00973
## 2     114.375     21.90444                   360.5  0.01162
## 3     112.500     23.80917                   360.5  0.00755
## 4     114.375     23.80917                   360.5  0.00814
## 5     112.500     21.90444                   361.5  0.01104
## 6     114.375     21.90444                   361.5  0.01270
```

```
h$month = ceiling(h$months.since.1960.01.01 %%12)
meanHumidity = tapply(h$unitless, h$month, mean)

# link humidity
d$humidity = meanHumidity[d$month]
```

Look at proportions of each tone:

```
d[,c("T1p","T2p",'T3p','T4p','T5p','T6p')] = t(apply(d[,c("T1","T2",'T3','T4','T5','T6')],1,prop.table))

# proportion of contour tones
d$prop.contour = d$T1p + d$T2p + d$T4p + d$T5p
```

Plot data

```
ggplot(d, aes(x=as.factor(month), y = T1p)) + geom_boxplot()
```

```
## Warning: Removed 25 rows containing non-finite values (stat_boxplot).
```

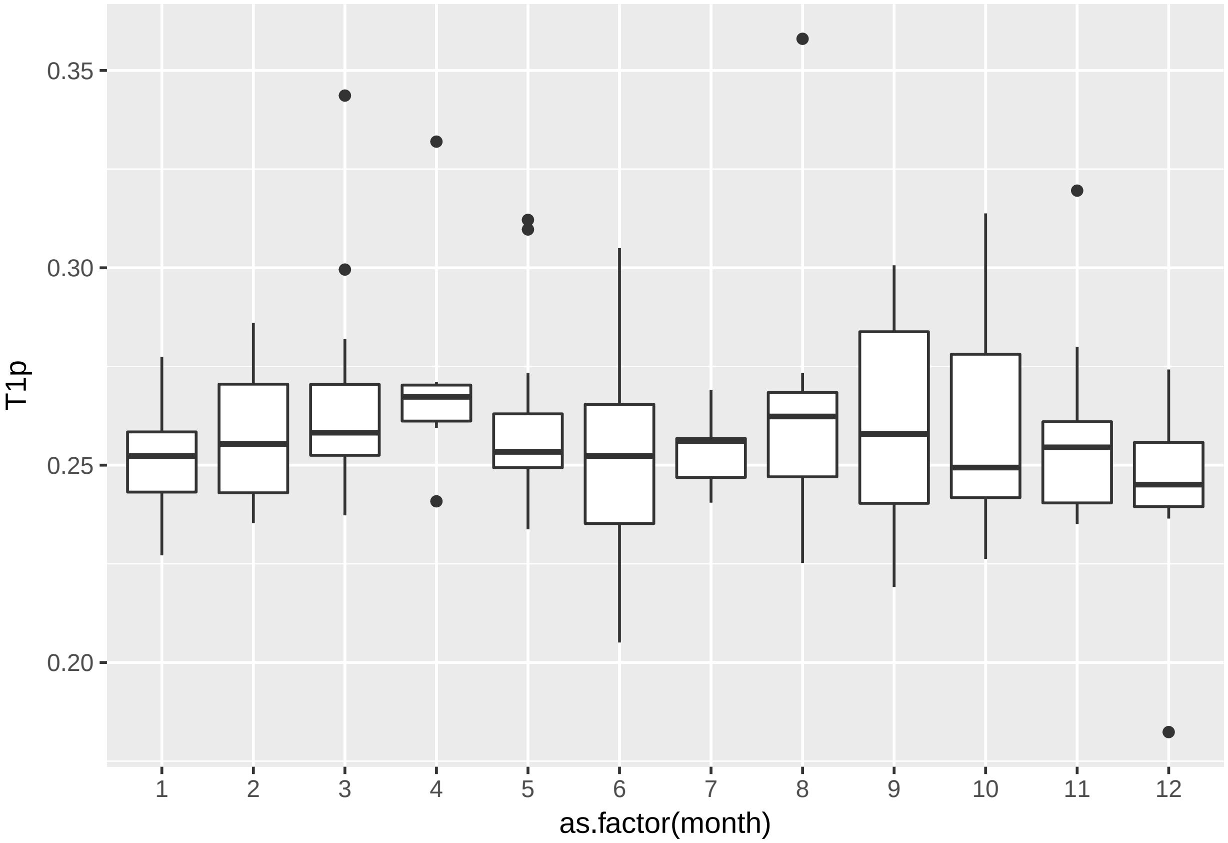


```
ggplot(d, aes(x=humidity, y = prop.contour)) + geom_point() + geom_smooth()
```

```
## `geom_smooth()` using method = 'loess' and formula 'y ~ x'

## Warning: Removed 25 rows containing non-finite values (stat_smooth).

## Warning: Removed 25 rows containing missing values (geom_point).
```

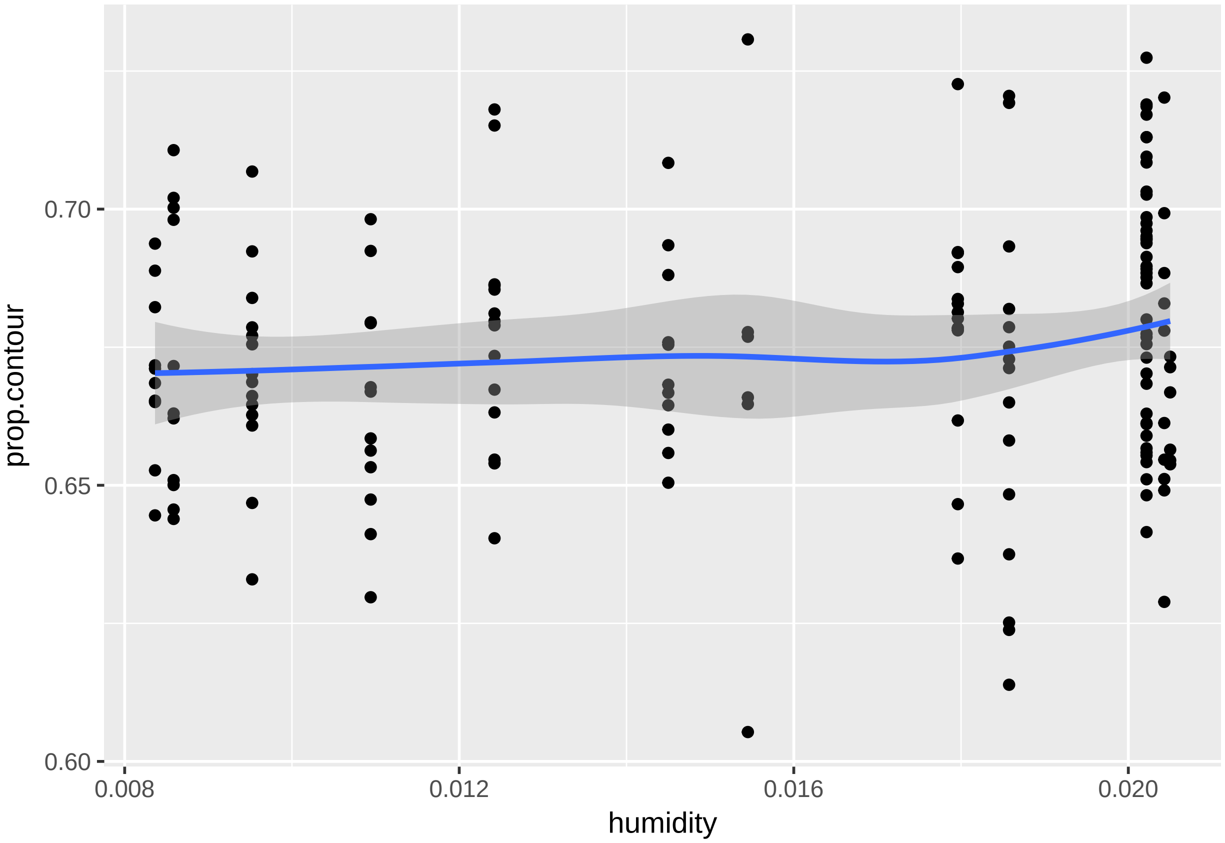


```
plot(1:12,ylim=c(0,0.5), xlab="Month", ylab="Proportion of use")
i = 1
for(x in c("T1p","T2p",'T3p','T4p','T5p','T6p')){
    points(tapply(d[,x],d$month,mean, na.rm=T),col=i, type='l')
    i = i +1

}
legend(1,0.5,legend=paste("Tone",1:6),col=1:6,pch=1, ncol=2)
```

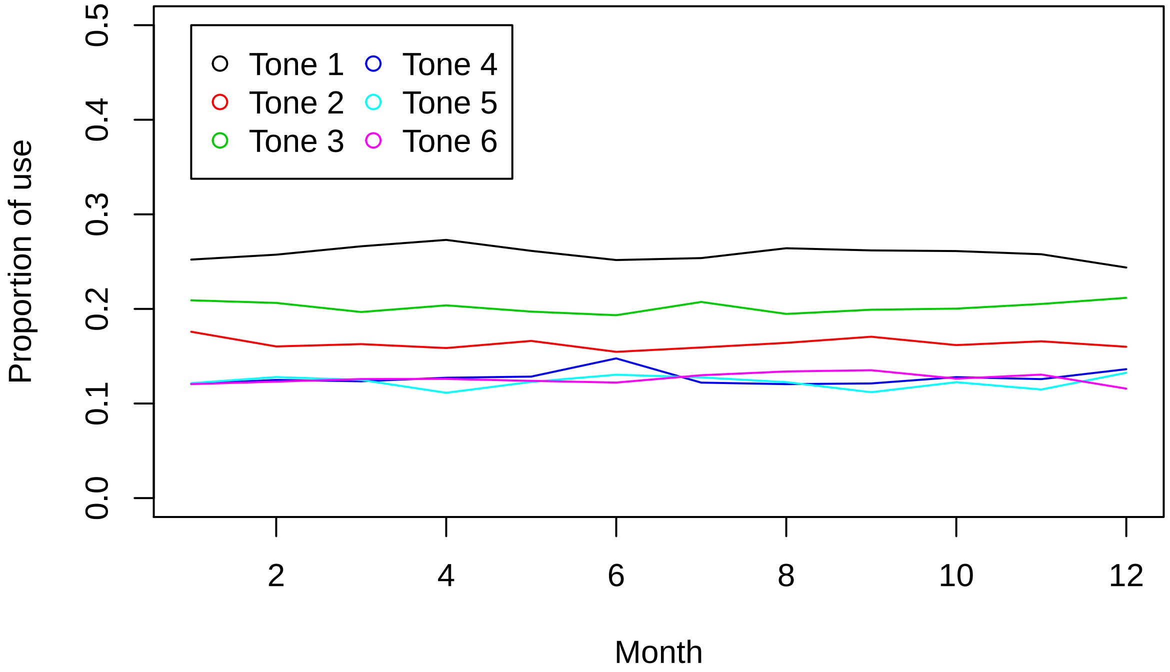

Run tests

Does humidity significantly contribute to the prediction of the proportion of contour tones used?

```
m0 = lmer(log(prop.contour) ~ 1 + (1|corpus), data=d)
m1 = lmer(log(prop.contour) ~ humidity + (1|corpus), data=d)
anova(m0,m1)
```

```
## refitting model(s) with ML (instead of REML)
```

```
## Data: d
## Models:
## m0: log(prop.contour) ~ 1 + (1 | corpus)
## m1: log(prop.contour) ~ humidity + (1 | corpus)
##    Df     AIC     BIC logLik deviance  Chisq Chi Df Pr(>Chisq)
## m0  3 -643.59 -634.29 324.80  -649.59
## m1  4 -641.87 -629.47 324.94  -649.87 0.2824      1     0.5952
```

```
summary(m1)
```

```
## Linear mixed model fit by REML ['lmerMod']
## Formula: log(prop.contour) ~ humidity + (1 | corpus)
##    Data: d
##
## REML criterion at convergence: -643.4
##
## Scaled residuals:
##      Min       1Q   Median       3Q      Max
## -3.05198 -0.65851  0.06731  0.62544  2.65945
##
## Random effects:
##  Groups   Name        Variance  Std.Dev.
```

```
##  corpus   (Intercept) 0.0003362 0.01834
##  Residual             0.0010868 0.03297
## Number of obs: 164, groups:  corpus, 2
##
## Fixed effects:
##             Estimate Std. Error t value
## (Intercept) -0.39297    0.01633 -24.058
## humidity     0.27244    0.58572   0.465
##
## Correlation of Fixed Effects:
##          (Intr)
## humidity -0.582
```

There is no significant effect.
