# Supplementary material for: Robust, Causal, and Incremental Approaches to Investigating Linguistic Adaptation
Source: Front Psychol. 2018 Feb 21;9:166. doi: 10.3389/fpsyg.2018.00166 (PMC5826341; doi:10.3389/fpsyg.2018.00166)

Testing the link between humidity and vowel use in Larry King's speech

Load libraries

```
library(gplots)
library(ggplot2)
library(lubridate)
library(mgcv)
library(grid)
library(gridExtra)
```

Helper functions for plot:

```
ggplot_dual_axis <- function(lhs, rhs, axis.title.y.rhs = "rotate") {
  # 1. Fix the right y-axis label justification
  rhs <- rhs + theme(axis.text.y = element_text(hjust = 0))
  # 2. Rotate the right y-axis label by 270 degrees by default
  if (missing(axis.title.y.rhs) |
      axis.title.y.rhs %in% c("rotate", "rotated")) {
    rhs <- rhs + theme(axis.title.y = element_text(angle = 270))
  }
  # 3a. Use only major grid lines for the left axis
  lhs <- lhs + theme(panel.grid.minor = element_blank())
  # 3b. Use only major grid lines for the right axis
  #     force transparency of the backgrounds to allow grid lines to show
  rhs <- rhs + theme(panel.grid.minor = element_blank(),
                     panel.background = element_rect(fill = "transparent", colour = NA),
                     plot.background = element_rect(fill = "transparent", colour = NA))
  # Process gtable objects
  # 4. Extract gtable
  library("gtable") # loads the grid package
  g1 <- ggplot_gtable(ggplot_build(lhs))
  g2 <- ggplot_gtable(ggplot_build(rhs))
  # 5. Overlap the panel of the rhs plot on that of the lhs plot
  pp <- c(subset(g1$layout, name == "panel", se = t:r))
  g <- gtable_add_grob(g1,
                       g2$grobs[[which(g2$layout$name == "panel")]], pp$t, pp$l, pp$b, pp$l)
  # Tweak axis position and labels
  ia <- which(g2$layout$name == "axis-l")
  ga <- g2$grobs[[ia]]
  ax <- ga$children[["axis"]]  # ga$children[[2]]
  ax$widths <- rev(ax$widths)
  ax$grobs <- rev(ax$grobs)
  ax$grobs[[1]]$x <- ax$grobs[[1]]$x - unit(1, "npc") + unit(0.15, "cm")
  g <- gtable_add_cols(g, g2$widths[g2$layout[ia, ]$l], length(g$widths) - 1)
  g <- gtable_add_grob(g, ax, pp$t, length(g$widths) - 1, pp$b)
  g <- gtable_add_grob(g, g2$grobs[[7]], pp$t, length(g$widths), pp$b)
  # add legend on top
  if ("guide-box" %in% g1$layout$name){
    dimGB1 <- c(subset(g1$layout, name == "guide-box", se = t:r))
```

```
    g <- gtable_add_grob(g,
                         g1$grobs[[which(g1$layout$name == "guide-box")]],
                         dimGB1$t, dimGB1$l, dimGB1$b, dimGB1$l, z=-Inf)
  }
  # Display plot with arrangeGrob wrapper arrangeGrob(g)
  library("gridExtra")
  grid.newpage()
  return(arrangeGrob(g))
}
```

Load data

```
d = read.csv("../data/King_segmentsByDate.csv")

d$vowelIndex = (d$v / (d$v+d$consonants))
```

Plot data

```
p1 = ggplot(d, aes(x=dayOfYear,y=vowelRatio)) +
  geom_smooth(colour='red') + ylab("Vowel Ratio") +
  xlab("Day of the year")

p2 = ggplot(d, aes(x=dayOfYear,y=humidity)) +
  geom_smooth(linetype = "dashed") + ylab("Humidity") +
  coord_cartesian(ylim=c(0.006,0.012))

x = ggplot_dual_axis(p1,p2,axis.title.y.rhs="Humidity")
```

```
## `geom_smooth()` using method = 'gam' and formula 'y ~ s(x, bs = "cs")'
## `geom_smooth()` using method = 'gam' and formula 'y ~ s(x, bs = "cs")'
```

```
plot(x)
```

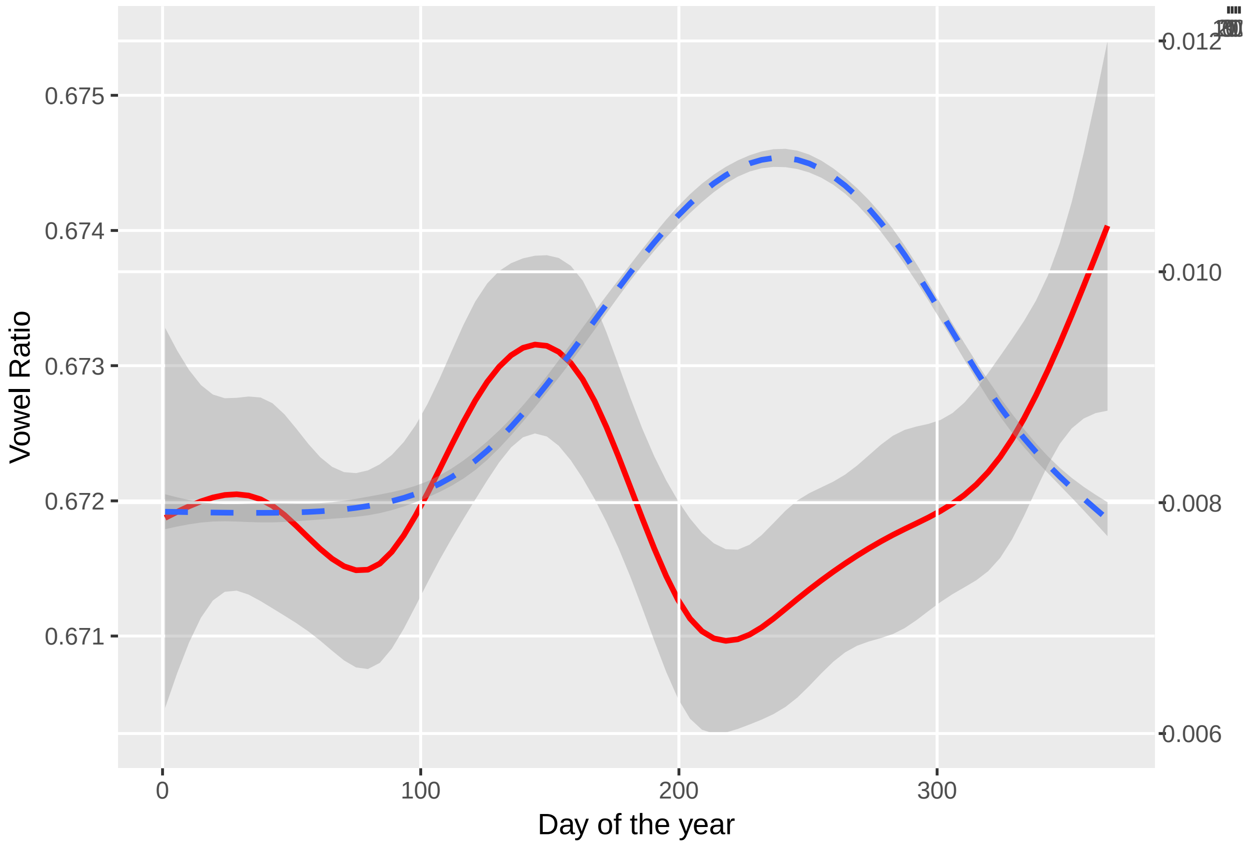


```
pdf("../results/HumidityAndVowelRatio.pdf", height = 4, width = 4)
plot(x)
dev.off()
```

```
## pdf
##   2
```

```
ggplot(d, aes(x=humidity,y=vowelRatio)) +
  geom_smooth() + geom_point(alpha=0.3)
```

```
## `geom_smooth()` using method = 'gam' and formula 'y ~ s(x, bs = "cs")'
```

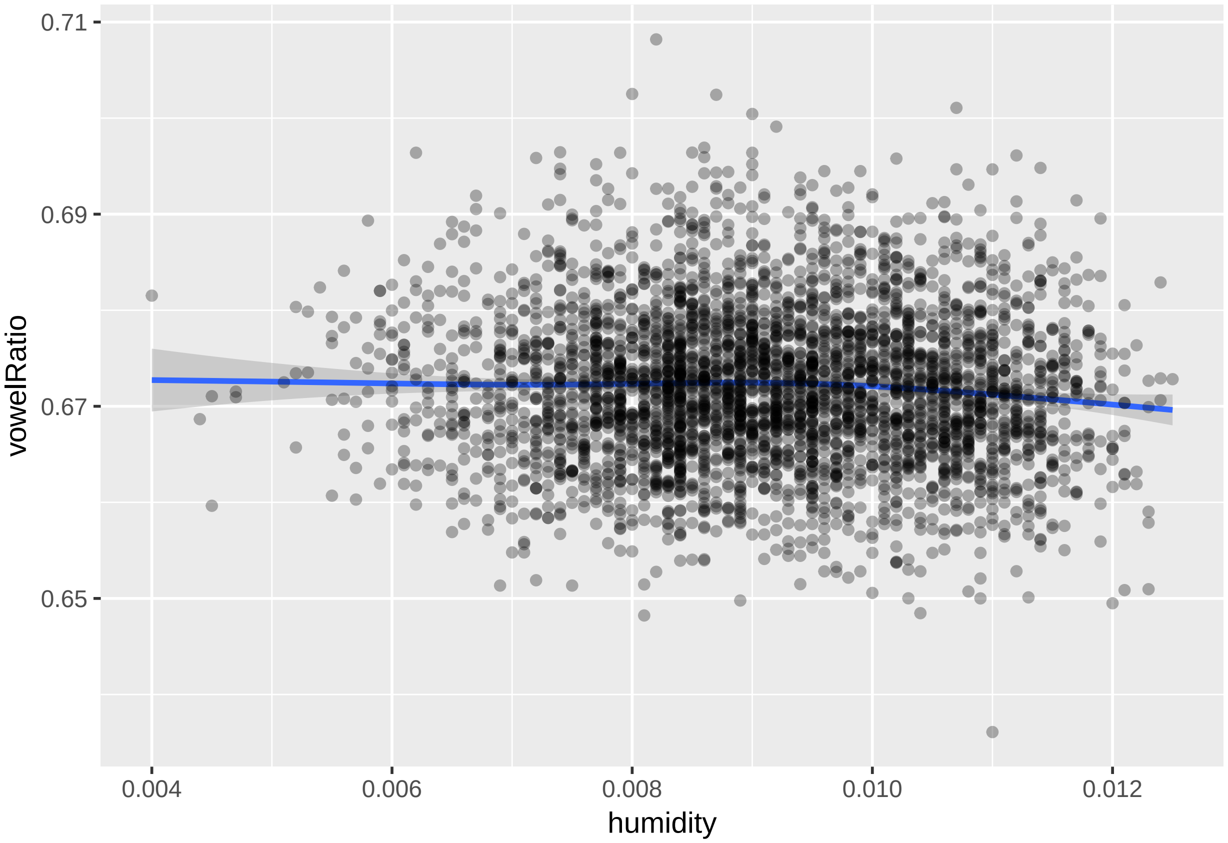

Run tests

```
m0= gam(vowelRatio~ s(humidity),data=d)
summary(m0)
```

```
##
## Family: gaussian
## Link function: identity
##
## Formula:
## vowelRatio ~ s(humidity)
##
## Parametric coefficients:
##              Estimate Std. Error t value Pr(>|t|)
## (Intercept) 0.6720667  0.0001354    4965   <2e-16 ***
## ---
## Signif. codes:  0 '***' 0.001 '**' 0.01 '*' 0.05 '.' 0.1 ' ' 1
##
## Approximate significance of smooth terms:
##               edf Ref.df    F p-value
## s(humidity) 2.851   3.61 4.95  0.0013 **
## ---
## Signif. codes:  0 '***' 0.001 '**' 0.01 '*' 0.05 '.' 0.1 ' ' 1
##
## R-sq.(adj) =  0.00468   Deviance explained = 0.547%
```

```
## GCV = 6.545e-05  Scale est. = 6.5379e-05  n = 3568
```

```r
plot(m0)
```

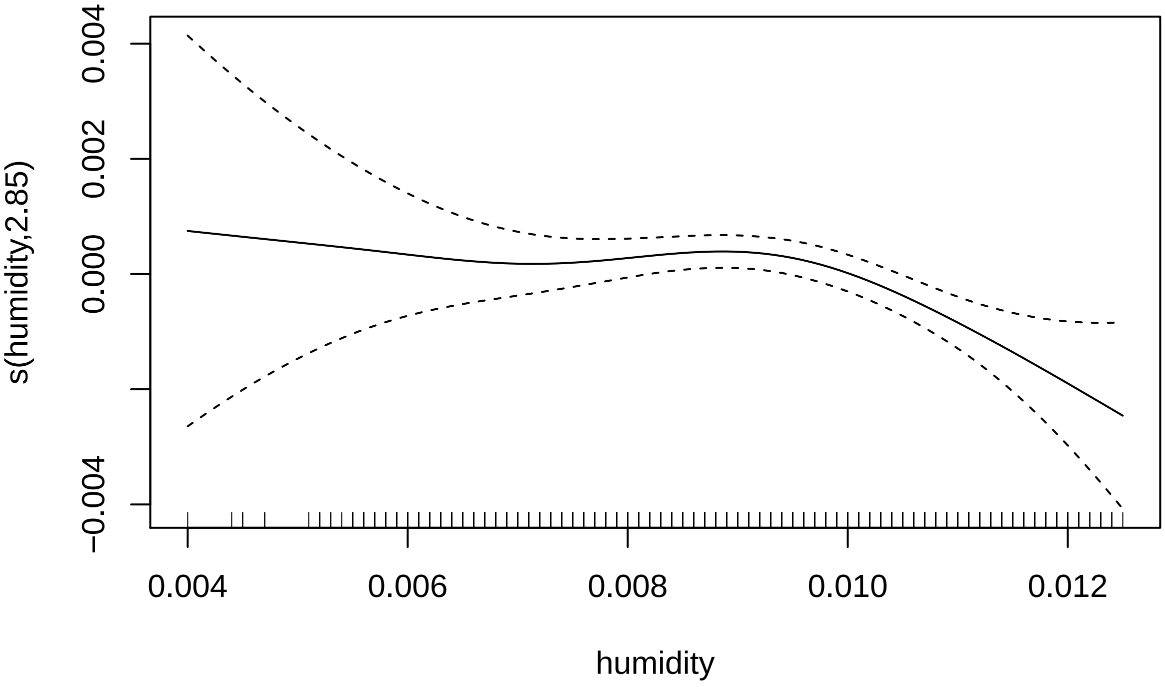

Supplement: Supplementary file 6 [file DataSheet6.PDF]
